# Two new compsognathid-like theropods show diversified predation strategies in theropod dinosaurs

**DOI:** 10.1093/nsr/nwaf068

**Published:** 2025-02-22

**Authors:** Rui Qiu, Xiaolin Wang, Shunxing Jiang, Jin Meng, Zhonghe Zhou

**Affiliations:** Key Laboratory of Vertebrate Evolution and Human Origins of Chinese Academy of Sciences, Institute of Vertebrate Paleontology and Paleoanthropology, Chinese Academy of Sciences, Beijing 100044, China; Natural History Museum of China, Beijing 100050, China; College of Earth and Planetary Sciences, University of Chinese Academy of Sciences, Beijing 100049, China; Key Laboratory of Vertebrate Evolution and Human Origins of Chinese Academy of Sciences, Institute of Vertebrate Paleontology and Paleoanthropology, Chinese Academy of Sciences, Beijing 100044, China; College of Earth and Planetary Sciences, University of Chinese Academy of Sciences, Beijing 100049, China; Key Laboratory of Vertebrate Evolution and Human Origins of Chinese Academy of Sciences, Institute of Vertebrate Paleontology and Paleoanthropology, Chinese Academy of Sciences, Beijing 100044, China; Division of Paleontology, American Museum of Natural History, New York, NY 10024, USA; Earth and Environmental Sciences, Graduate Center, City University of New York, New York, NY 10016, USA; Key Laboratory of Vertebrate Evolution and Human Origins of Chinese Academy of Sciences, Institute of Vertebrate Paleontology and Paleoanthropology, Chinese Academy of Sciences, Beijing 100044, China; College of Earth and Planetary Sciences, University of Chinese Academy of Sciences, Beijing 100049, China

**Keywords:** Early Cretaceous, Jehol Biota, Yixian Formation, Sinosauropterygidae, North China craton

## Abstract

The Compsognathidae was originally considered an early-diverging clade of coelurosaur theropods. However, recent study suggests that Compsognathidae is not monophyletic. Here, we describe two new compsognathid-like species, *Sinosauropteryx lingyuanensis* sp. nov. and *Huadanosaurus sinensis* gen. et sp. nov. from the Lower Cretaceous Yixian Formation of Dawangzhangzi (Lingyuan, Western Liaoning, China). The phylogenetic results indicate that all compsognathid-like theropods from the Early Cretaceous Jehol Biota form a monophyletic group Sinosauropterygidae nested among early-diverging coelurosaurs. Morphological comparison between various species of sinosauropterygids from the Early Cretaceous of Northeast China, combined with the phylogenetic results, suggests that at least three distinct hunting strategies were present among coeval species. The diversification of theropods should be attributed to the landscape caused by the destruction of the North China craton.

## Teaser

The morphological evidences and abdominal contents of two new sinosauropterid species reveal a diversification in hunting styles among the sinosauropterygids, which can be attributed to the landscape caused by the North China craton destruction.

## INTRODUCTION

The Compsognathidae was traditionally regarded as a monophyletic group of early-diverging coelurosaurs characterized by relatively small body size (often less than 1 meter in length) and serval morphological characters, such as fan-shaped mid- to posterior dorsal neural spines. The first compsognathid genus *Compsognathus* was found from the Upper Jurassic of Bavaria in the 1860s [1]. No new unequivocal compsognathid genera were reported until the 1990s. *Sinosauropteryx*, found from the Early Cretaceous Jehol Biota, was the second compsognathid genus reported and the first dinosaur known to be covered with feather-like integumentary structures [2,3]. Up to now, ten genera and species have been classified within this clade, including *Compsognathus* and *Juravenator* [4] from the Upper Jurassic of Western Europe, *Sinosauropteryx, Huaxiagnathus* [5], *Sinocalliopteryx* [6] and *Xunmenglong* [7] from the Lower Cretaceous of China, *Scipionyx* [8] from the Lower Cretaceous of Italy, *Aristosuchus* from the Lower Cretaceous of the UK [9] and *Mirischia* [10] from the Lower Cretaceous of Brazil. However, a recent phylogenetic study suggests that these ‘putative compsognathids’ are classified in various lineages among Tetanurae [11], rather than within a monophyletic group nested among early diverging coelurosaurs in the former phylogenetic studies [12–14]. All phylogenetic results, however, agree that *Sinosauropteryx, Huaxiagnathus, Sinocalliopteryx* and *Mirischia* are closely related and have been placed along the basal branches of Coelurosauria [11–14].

Here we describe two new compsognathid-like theropod species *Sinosauropteryx lingyuanensis* sp. nov. and *Huadanosaurus sinensis* gen. et sp. nov., based on two specimens discovered 20 years ago from the Lower Cretaceous Yixian Formation of Dawangzhangzi (Lingyuan, Western Liaoning, China). Parsimony analysis based on a traditional theropod matrix and an Ontogenetic State Partitioning matrix support that all compsognathid-like theropods from the Early Cretaceous Jehol Biota are classified within Sinosauropterygidae. The sinosauropterygids from the Jehol Biota present three different predation strategies among small theropods from the Early Cretaceous of Northeast China, suggesting that the landscape caused by the dynamic crust movement may have influenced the diversification of theropods.

## RESULTS AND DISCUSSION

### Systematic palaeontology

Dinosauria Owen, 1842

Saurischia Seeley, 1888

Theropoda Marsh, 1881

Coelurosauria Von Huene, 1914

Sinosauropterygidae Ji et Ji, 1996

*Sinosauropteryx* Ji et Ji, 1996

*Sinosauropteryx lingyuanensis* sp. nov.

Species name [urn: lsid: zoobank.org: act:7F6AE3A8-447F-46A4-8E0A-C12A698F9213]

**Etymology.** ‘*lingyuan*’, a Chinese county-level city where the holotype was found.

**Figure 1.**
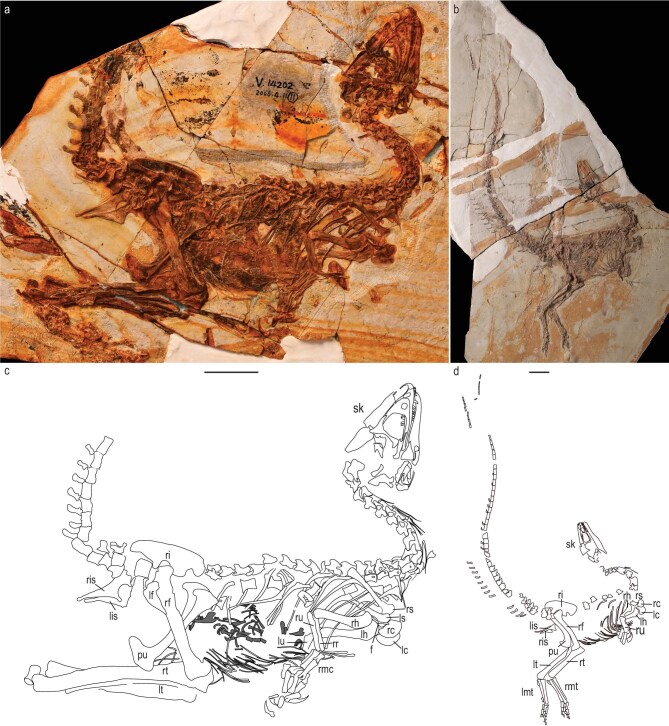
Holotype of *Huadanosaurus sinensis* gen. et sp. nov. (IVPP V 14202) and *Sinosauropteryx lingyuanensis* sp. nov. (IVPP V 12415). (a, b) Photograph of IVPP V 14202 and IVPP V 12415, respectively. (c, d) Line drawing of IVPP V 14202 and IVPP V 12415, respectively. Black shading indicates the impression made by the skeleton. Dark grey shading indicates the mammalian bony stomach content. f, furcula; lc, left coracoid; lf, left femur; lh, left humerus; li, left ilium; lis, left ischium; lmt, left metatarsal; ls, left scapula; lt, left tibia; lu, left ulna; pu, pubis; rc, right coracoid; rd, right dentary; rf, right femur; rh, right humerus; ri, right ilium; ris, right ischium; rmc, right metacarpal; rmt, right metatarsal; rr, right radius; rs, right scapula; rt, right tibia; ru, right ulna. Scale bar, 10 cm.

**Holotype.** IVPP V 12415 (Fig. 1b and d), an almost complete skeleton, missing the feet and the posterior caudal vertebrae. It most likely represents a juvenile according to the neural arch separated from the centrum of the dorsal vertebrae.

**Locality and Horizon.** Dawangzhangzi, Lingyuan, Liaoning Province, China. The Dawangzhangzi bed of Yixian Formation, Lower Cretaceous (125 Ma, Barremian).

**Diagnosis.** Differs from other sinosauropterygids in possessing the following autapomorphies: the jugal ramus of the maxilla subequal to the length of the snout; maxilla relatively low and elongated, maxillary fenestra large and subequal in size to the external naris; absence of the ischiatic boot.

**Figure 2.**
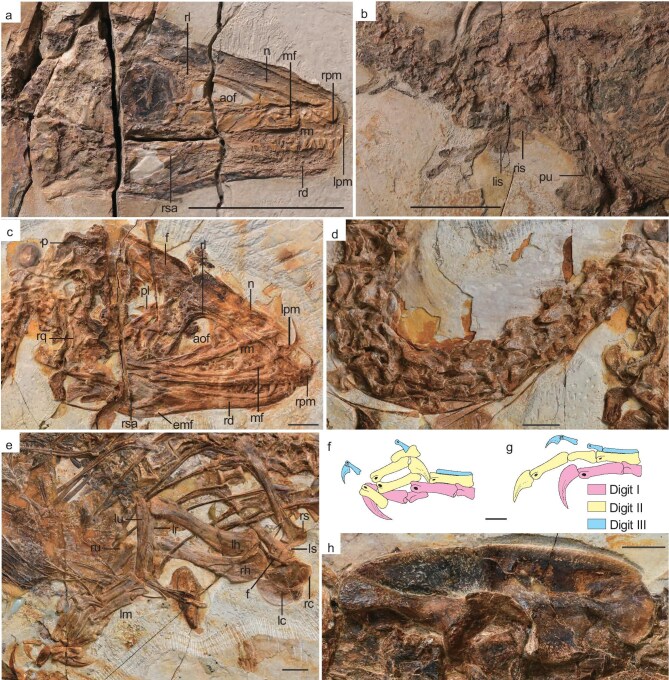
Anatomy of *Sinosauropteryx lingyuanensis* sp. nov. IVPP V 12415 (a, b) and *Huadanosaurus sinensis* gen. et sp. nov. IVPP V 14202 (c–h). (a) Skull and mandible of IVPP V 12415. (b) Pelvic girdle. (c) Skull and mandible of IVPP V 14202. (d) Cervical vertebrae. (e) Shoulder girdle and the forelimb. (f) Line drawing of the manus. (g) Reconstruction of the manus. (h) Ilium. aof, antorbital fenestra; emf, external mandibular fenestra; f, furcula; fr, frontal; lc, left coracoid; lh, left humerus; lpm, left premaxilla; lr, left radius; ls, left scapula; lu, left ulna; mf, maxillary fenestra; n, nasal; p, parietal; pl, plate; pu, pubis; r, ridge; rc, right coracoid; rd, right dentary; rh, right humerus; rl, right lacrimal; rm, right maxilla; rpm, right premaxilla; rq, right quadrate; rs, right scapula; rsa, right surangular; ru, right ulna. Scale bars: 5 cm (a and b), 1 cm (c–h).

**Description.** With a total length of ∼120 cm, the holotype of *Sinosauropteryx lingyuanensis* is the largest known *Sinosauropteryx* specimen. The rostrum is proportionally low and long, approximately half the length of the skull (Fig. 2a; Table S1), consistent with other sinosauropterygids except for *Sinocalliopteryx* [6]. The maxilla is longer than that of *S. prima*, with a length-to-depth ratio of ∼2.75 (2.01 in *S. prima*). The anterior and ascending ramus of the maxilla are separated by a weak concavity, as in *S. prima* [15]. The maxilla bears a large maxillary fenestra, roughly equal in size to the external naris, differing from the smaller openings in other compsognathid-like theropods [5,16–18]. The jugal ramus is extremely elongated, approximately four times of the anterior body of the maxilla. The lacrimal is ‘L’-shaped. The mandible is long and gracile, with its dorsal and ventral margins running parallel. On the anterior cervical vertebrae, the postzygapophysis extends beyond the posterior articular face of the centrum (Fig. S1a). The length of the postzygapophysis equals that of the prezygapophysis, contrasting with the short postzygapophysis in other sinosauropterygids [2,4,6,18]. In the middle cervical vertebrae, the posterior articular surface extends further ventrally than the anterior articular surface, creating an angled shape. This feature is likely due to the presence of a markedly convex rostral facet of the centrum, a typical feature associated with the opisthocoelous condition shared by many tetanuran theropods [19]. Some cervicals of *Sinosauropteryx* are also angled, but not to the degree seen in this new species [15]. The ventral margin of the centrum of the cervical vertebrae is straight and differs from the concave ventral margin observed in the centrum of the cervical vertebrae of *S. prima* [15]. The cervical ribs are roughly twice the length of the corresponding centra, similar to other sinosauropterygids [17,20]. As in *S. prima* [2], the extremely long tail consists of more than 60 caudal vertebrae (Fig. S1b). In the anterior caudal vertebrae, an accessory neural spine is present at the base of the anterior margin of the neural spine, as in *S. prima* [15]. The coracoid is semicircular. The length of the forelimb (humerus + radius + metacarpal II) is less than half of the hindlimb (femur + tibia + metatarsal III). The delpectoral crest extends about half the length of the humerus, as in *S. prima* [2]. The humeral shaft is slightly more slender than in *S. prima* (Fig. S1c). The ulna bears a robust olecranon. The ungual phalanx of digit I is slightly shorter than the humerus and not strongly curved. The flexor tubercle of ungual I-2 is well-developed and subequal in length with the articular facet. The pubis is anteroventrally pointed. The pubis bears a strongly developed pubic boot that possesses a posterior process much stronger than the anterior one [21]. The nearly triangular obturator process locates at the proximal end of ischium, as in *S. prima* and *Mirischia* [15,22]. Unlike other compsognathid-like theropods [5,6,15,16], there is no prominent ischial boot at the distal end of the ischium. The hindlimb is slender, with the femur ∼90% the length of the long and slender tibia. Metatarsal II∼IV are similar in length and width, ∼60% of the tibia length (Fig. S1d). Metatarsal V is short and splint-like, about one quarter of metatarsal III length. The third digit is the longest, followed by the second and fourth. The length of digits decreases gradually from proximal one to distal. All pedal unguals are small and weakly curved.

*Huadanosaurus sinensis* gen. et sp. nov.

Genus name [urn: lsid: zoobank.org: act:9AC095B9-CC01-43C7-8470-B29D14A72E9B]

Species name [urn: lsid: zoobank.org: act: BCB3196D-3204–47E2-8681-38D4CAE9BA9A]

**Etymology.** ‘*Huadan*’, a Chinese word meaning the birthday of a great person or a great institution, commemorating the 75th anniversary of the founding of the People's Republic of China and the Chinese Academy of Sciences, the 95th anniversary of the founding of the Palaeontological Society of China and the Institute of Vertebrate Paleontology and Paleoanthropology, Chinese Academy of Sciences, and the 40th anniversary of the founding of the Chinese Society of Vertebrate Paleontology; ‘*saurus*’, Greek for lizard. ‘*sine*’, Latin referring to China.

**Holotype.** IVPP V 14202 (Fig. 1a and c), an almost complete skeleton, missing the feet and the distal caudal vertebrae. It may represent a juvenile individual owing to the unfused neurocentral sutures on the vertebrae and the scarred surface of its bones [18] and a relatively large skull (∼0.37 of the presacral vertebral column length).

**Locality and Horizon.** The same as *Sinosauropteryx lingyuanensis.*

**Diagnosis.** Differs from other sinosauropterygids in possessing the following autapomorphies: large oval concavity between the anterior margin of the antorbital fossa and the maxilla; large lacrimal recess on the lacrimal, U-shaped bifurcation at the posteroventral margin of the dentary, small and crescent-shaped external mandibular fenestra, fan-shaped neural spine on the axis, strongly anteroposteriorly elongated centrum in the posterior cervical vertebrae, small pleurocoel on centrum of the anterior dorsal vertebrae, the coracoid elongated lateromedially, not oval-shaped, the supracetabular crest well developed on the ilium, the obturator process >70% of the length of the ischial shaft, and the tibia ∼1.3 times of the femoral length.

**Description.** The skull length (100.59 mm) is subequal to that of the femur (105.69 mm). The anteroposterior length of the main body of the premaxilla is greater than the dorsoventral height (Fig. 2a and Fig. S2a). The maxillary and nasal processes are similar in length. A circular subnarial foramen is located at the lower half of the posterior margin of the premaxillary body, contrasting with other theropods that usually have the subnarial foramen at a higher position. The maxilla is much deeper than that of other sinosauropterygids; the ratio of its length to the maximum depth is 1.72. The anterior process is confluent with the ascending process as in *Sinocalliopteryx* [6] and *Scipionyx* [17]. There is no concave area on the anterior margin that divides the anterior and ascending process as in other compsognathid-like theropods [5,15,16,18]. A large and oval fossa is present between the anterior margin of the maxilla and the antorbital fossa (Fig. S2c). The slit-like promaxillary fenestra is located in the anterodorsal corner of the antorbital fossa, similar to *Zuolong* [12] and some tyrannosauroids [23–25]. The promaxillary fenestra is absent in most sinosauropterygids. The ‘L’-shaped lacrimal possesses a triangular pneumatic foramen at the junction of the anterior process and ascending process, suggesting existence of the lacrimal sinus (Fig. S2b), as in *Alioramus* [26]. The dorsal and ventral margins of the dentary are subparallel. A small crescent-shaped external mandibular fenestra is enclosed by the dentary and angular. The crowns of all preserved premaxillary teeth are straight. Both the mesial and distal carinae of the premaxillary teeth are unserrated, as in *Sinosauropteryx* and *Compsognathus* [15,16]. The maxillary teeth are laterally compressed. A labial depression is present on the crown base. The distal carina is strongly labially deflected (Fig. S2d), as in tyrannosauroids [27,28] and dromaeosaurids [29]. The denticles are present on the distal carina starting from the sixth maxillary tooth. In the postcranium, the axial centrum is strongly angled in the lateral view (Fig. S3b), different from a rectangular centrum in other compsognathid-like theropods [15,17,20]. The centrum of the seventh cervical vertebra is the longest, ∼1.9 times the length of the axial centrum (Fig. 2d, Fig. S3a and S3b). The neural spine is prominent on all preserved cervical vertebrae. The lateral surface of the first two dorsal vertebrae bears an oval pleurocoel (Fig. S3c and S3d), which is absent on all dorsal vertebrae in other sinosauropterygids [10,15–17]. The scapula is strap-like, with a slight expansion at its distal end. Unlike the subcircular coracoid of other compsognathid-like theropods [15–18,20], the coracoid is strongly lateromedially elongated and the posteroventral process shows a further extension (Fig. 2e). The small coracoid foramen is present near the scapular articular surface. The angle between the two rami of the furcula is 144 degrees. The combined length of humerus and radius is 52% of that of the femur and tibia. The humerus is ∼1.4 times the length of the ulna. Metacarpal I is less than half of the metacarpal II length. Metacarpal II is the longest and most robust metacarpal, and is ∼37.7% of the humerus length. Metacarpal III is less than half of the width of metacarpal II. All unguals are strongly curved. Phalanx I-2 is the largest ungual, whereas phalanx III-4 is the smallest (Fig. 2f and 2g). The ilium is shorter than the femur. The supracetabular crest is well developed, contrasting with other sinosauropterygids where this crest is extremely reduced or absent [10,15,17,20]. A longitudinal ridge above the supracetabular crest divides the lateral surface of the ilium into two parts (Fig. 2h, Fig. S4), which was regarded as a synapomorphy of tyrannosauroids [30]. The pubis is ∼86% of the femoral length; its shaft is straight and possesses a vertical orientation. The pubic boot possesses a small anterior process and a large posterior process (Fig. S4b). The ischiadic shaft is nearly straight. The base of the obturator process accounts for >70% of the total length of the ischiadic shaft. There is no distinct femoral neck between the femoral head and shaft. The tibia is ∼1.28 times the femur length. This ratio is greater than those in other compsognathid-like theropods [5,6,15,20]. The fibular crest is ∼25% of the way down the shaft of the tibia from the proximal end.

### Diversified predation strategies of sinosauropterygids

**Figure 3.**
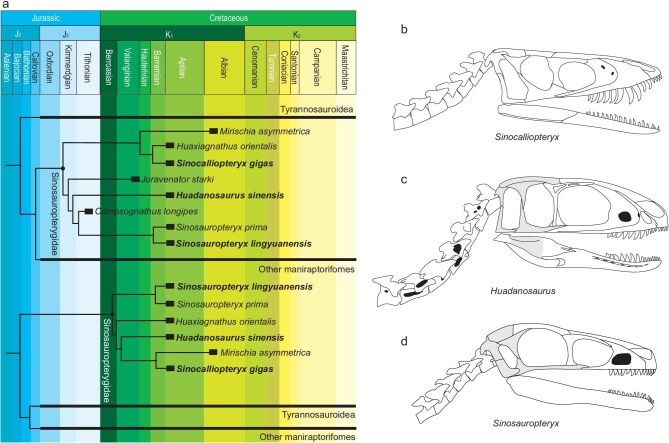
Time-calibrated phylogeny of sinosauropterygids and comparisons of skull and neck morphology among sinosauropterygids from Yixian Formation. (a) Simplified phylogeny showing position of *Sinosauropteryx lingyuanensis* and *Huadanosaurus sinensis* based on the reduced strict consensus of the most parsimonious trees from the TWiG data matrix (upper) and the most parsimonious tree from the OSP matrix (lower), respectively. (b) Lateral reconstruction of skull and neck of *Sinocalliopteryx* based on JMP-V-05–8–01. (c) Lateral reconstruction of skull and neck of *Huadanosaurus* based on IVPP V 12415 and NIGP 127586. (d) Lateral reconstruction of skull and neck of *Sinosauropteryx* based on IVPP V 14202. Light grey shading indicates the unknown parts.

Both the strict consensus of the most parsimonious trees from the phylogenetic analyses based on the traditional theropod matrix [13] and the Ontogenetic State Partitioning matrix [14] suggest that all the compsognathid-like theropods from the Early Cretaceous Jehol Biota, along with *Mirischia*, form a clade of early-diverging coelurosaurs (Fig. 3, Fig. S7). The only difference between the two results is the nesting of *Compsognathus* and *Juravenator*. Sinosauropterygidae Ji et Ji, 1996 is used instead of Compsognathidae for this clade because *Compsognathus* has been excluded from the clade that bears its name in the phylogenetic result based on the Ontogenetic State Partitioning matrix. Although *Huadanosaurus* shares some characteristics with tyrannosauroids, such as the fused nasals at the midline, strongly labially deflected distal carina on lateral teeth, a prominent surangular shelf on the lateral surface of surangular, and a longitudinal ridge above the supracetabular crest on the lateral surface of the ilium [27,28,30], the phylogenetic results suggest that these characteristics can also be found in several theropods from different lineages. Therefore, the similarity between *Huadanosaurus* and tyrannosauroids is likely due to convergence rather than sharing a common ancestor. The phylogenetic results based on the Ontogenetic State Partitioning matrix also support the notion that, although the two new specimens belong to two juvenile individuals, they do not represent juvenile specimens of the mature theropod taxa from the Jehol Biota.

Some skull features of *Huadanosaurus* similar to those of tyrannosauroids suggest that its jaw likely possesses a bite force much greater than other sinosauropterygids. Although the surangular is not completely preserved, a prominent process on its lateral surface suggests the presence of a prominent surangular shelf on the mandible of *Huadanosaurus*, providing an attachment site for the adductor muscle [31,32]. The adductor muscle of the mandible in *Huadanosaurus* is much stronger than that of other sinosauropterygids due to the absence or weak prominence of their surangular shelf. The deep skull and mandible, combined with the fused nasal, optimize the skull for resistance to large biting stress [31]. The neural spines of the cervical vertebrae are relatively higher than other sinosauropterygids, providing a larger attachment site for the muscle on the neck to support the relatively heavy skull [32]. The presence of a labial depression at the crown base is similar to those of Alioramini and subadult tyrannosauroids [27,28]. However, the premaxillary teeth of *Huadanosaurus* are extremely slender, in contrast to the strong premaxillary teeth with a ‘U-shaped’ oval cross-section found in the derived tyrannosauroids [27], which can bear a large bite force in order to control large prey. The weak premaxillary teeth of *Huadanosaurus* indicate the absence of a tyrannosaurid-style puncture-pull feeding mechanism. The presence of small and disarticulated mammal skeletal remains preserved in the abdominal cavity of *Huadanosaurus* suggests that it fed on prey much smaller than itself (Fig. S5), similar to the condition in the juvenile tyrannosauroids [33]. The mammal remains form two patches: the dorsal patch consists of the skeletal elements of the eutriconodonts, which are more robust than those of the eutherian in the ventral patch. Based on the relative positions of the jaw, skull fragments, ribs, caudal vertebrae and other skeletal elements, it can be inferred that the body of the eutriconodon was oriented with its head being posteriorly directed, while the orientation of the eutherian is opposite to that of the eutriconodon. The distribution of the disarticulated, yet well-preserved, skeletal elements suggests that the mammals were swallowed in as a whole. The preserved condition of these mammal remains appear consistent with the relatively weak manus of *Huadanosaurus*, where the proximal articular surface of all manual phalanges is only slightly concave, allowing for a limited range of flexion of the manual digits. This indicates that the grasping ability of the manus of *Huadanosaurus* is insufficient for tearing the prey apart or grasping them for biting. *Huadanosaurus* likely caught the small prey with its mouth, quickly killed its prey using the strong bite force of its maxillary teeth, and swallowed the prey whole during the hunt. The surface of the mammal bones is relatively smooth with some light etching caused by gastric acids, indicating that the mammal remains had not been in the stomach of *Huadanosaurus* for too long. The preserved condition suggests that *Huadanosaurus* and its prey were likely active during the same time of the day. It has long been known that Mesozoic mammals were probably nocturnal [34], and if true, it would indicate that at least some sinosauropterygids were also nocturnal. Furthermore, it can be postulated that sinosauropterygids were already physiologically warm-blooded animals active at night, especially in the relatively cold climate of the Jehol Biota [35]. This is consistent with the fact that feather-like structures, possibly for insulation, had developed in sinosauropterygids. In the Early Cretaceous Jehol Biota, several mammalian lineages had various locomotor modes [36]. The eutriconodont content is more similar to *Meemannodon lujiatunensis* but differs from other eutriconodonts in having relatively long molariform, cusps b and c distinct, first molariform significantly smaller than others, and m3 the largest of lower cheek teeth, suggesting it should be nested within Gobiconodontidae [37], most of which are terrestrial [36]. Although the eutherian content is badly preserved, it likely spent a considerable time in trees, as most Jurassic and Early Cretaceous eutherians were arboreal or scansorial [36,38]. The stomach contents consist of mammals with different locomotor modes rather than from various lineages of animals with the same locomotor mode, indicating that juvenile *Huadanosaurus* had a relatively strong preference for food.

Although the body size of the juvenile *Huadanosaurus* is comparable to that of juvenile *Sinosauropteryx*, no competition appears to have been present between these two sinosauropterygids owing to different ecomorphological specializations and various prey items. This is evidenced by the lizard remains found in the abdominal cavity of one *Sinosauropteryx* specimen [2]. The orientations of the skull and vertebrae indicate that this lizard may have been dismembered into at least two pieces, similar to the condition of the abdominal contents preserved in the rib cage of *Compsognathus* [39]. *Sinosauropteryx* and *Compsognathus* share similar cranial morphologies, including a long and slender skull and lower jaws, the absence of the surangular shelf, and small teeth, suggesting a relatively small bite force. In contrast to *Compsognathus*, the forelimbs of *Sinosauropteryx* are extremely short [6], limiting its ability to tear prey into pieces without assistance from its hindlimbs. However, the hindlimb of *Sinosauropteryx* is relatively slender, and the processes for the attachment of the neck muscle on its cervical vertebrae are weak, suggesting it could only prey on relatively gracile targets such as lizard or insects. In addition to differences in hunting style and prey, the active time of *Sinosauropteryx* may differ from *Huadanosaurus*. The feather filaments covering the body of *Sinosauropteryx* could be used as camouflage under abundant direct sunlight in an open area [40], suggesting that *Sinosauropteryx* may have been diurnal, unlike *Huadanosaurus*, which may have been nocturnal. *Sinocalliopteryx* is the largest known sinosauropterygid [6], and its hunting styles may also differ from that of *Huadanosaurus*. The skull and neck of *Sinocalliopteryx* are more similar to those of *Sinosauropteryx* rather than to *Huadanosaurus*. The slender mandible, indistinct surangular shelf and slim teeth indicate a relatively weak bite force in *Sinocalliopteryx*. The abdominal contents of *Sinocalliopteryx* indicate that it could hunt relatively large prey, such as dromaeosaurids and birds [41]. As in other sinosauropterygids, the forelimb of *Sinocalliopteryx* is short and slender, however its hindlimb is much stronger than that of *Sinosauropteryx*, allowing it to control and kill relatively large prey using its hindlimbs. Morphological evidence combined to abdominal contents reveal that at least three distinct hunting styles were present among sinosauropterygids from the Yixian Formation. Although *Sinocalliopteryx* is found in the Sihetun Bed rather than at Dawangzhangzi, all fossil-bearing horizons in the Yixian Formation share nearly the same age [42]. The differences in hunting style among the sinosauropterygids from the Yixian Formation indicate that these species occupy different ecological niches in the sympatric ecosystem.

**Figure 4.**
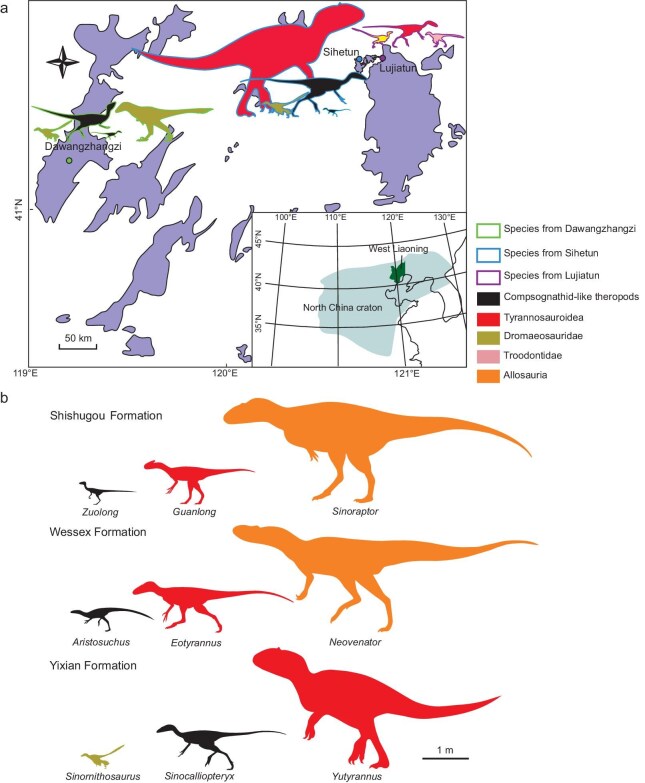
Comparison between the species combination of the theropods from the Early Cretaceous of Northeast China and other localities. (a) Geographic distribution of theropod fossil sites of Yixian Formation in Cretaceous basins (indicated in purple) of Western Liaoning Province. (b) Comparison between the species combination of the theropods from different localities. The theropods from the Upper Jurassic Shishugou Formation of Xinjiang are *Zuolong, Guanlong* and *Sinoraptor*. The theropods from the Lower Cretaceous Wessex Formation of the Isle of Wight are *Aristosuchus, Eotyrannus* and *Neovenator*. The theropods from the Lower Cretaceous Yixian Formation of West Liaoning are *Sinornithosaurus, Sinocalliopteryx* and *Yutyrannus*.

The morphological diversification within the same theropod clades from the same sedimentary unit is exceedingly rare. In the Yixian Formation, the morphological diversification is evident not only in sinosauropterygids, but also in dromaeosaurids and tyrannosauroids. The distinctiveness of the theropods from the Jehol Biota is further exemplified by the gigantism in Proceratosauridae. While carcharodontosaurids were the apex predators in many regions of the world during the Early Cretaceous, including regions around the Jehol Biota such as Japan and Korea [43,44], no evidence of their presence has been uncovered in the Jehol Biota hitherto. Instead, the Early Cretaceous Jehol Biota is characterized by the dominance of the colossal tyrannosauroid (body length >7 m) *Yutyrannus* [45] and *Sinotyrannus* [46]. This period marks the only instance of gigantism within Proceratosauridae and the sole occurrence of gigantism among tyrannosauroids during the Early Cretaceous. During the Late Jurassic and Early Cretaceous, tyrannosauroids were typically mid-sized predators in the ecosystem. However, in the Yixian Formation, the tyrannosauroids were the top predators and some compsognathid-like theropods were the mesopredators. The unique composition of theropod species in the Yixian Formation suggests a lack of species exchange with other regions due to geographic barriers created by crustal contraction and extension, and sporadic magmatism during the Early Cretaceous [47,48]. This isolation prevented the ecosystem on the North China craton from interacting with other areas during that time. Additionally, the destruction of the North China craton resulted in the formation of many small and isolated rift basins during the Early Cretaceous [48], which could shape landscape dynamics, leading to the uneven separation of terrestrial species. The diversity of troodontids in the Yixian Formation was primarily concentrated in Lujiatun [49–53], with only a single specimen outside this locality [54], despite the abundance of theropod specimens from other lineages in the Yixian Formation. Although mammalian bones, which can be classified into different lineages, were also found in the abdominal cavity of a small-sized tyrannosauroid specimen (NGMC 2124) from the Yixian Formation [11], it and *Huadanosaurus* were geographically distributed in different basins [55]. In Northeast China ∼125 Ma, theropods from various lineages occupied similar ecological niches in different areas, while those from the same lineage assumed diverse ecological roles within the same locale (Fig. 4). This indicates the presence of numerous small, isolated rift basins, which likely hindered species intermixing and intensified competition among species. The heightened selection pressure resulting from species competition in these isolated rift basins could have propelled the diversification of theropods. The landscape dynamics are known for playing an important role in biosphere diversification [56] and might have affected the diversification of theropods in the Jehol Biota.

## MATERIALS AND METHODS

### Material and the age of the specimen-bearing horizon

Both the specimens studied in this paper, IVPP V 12415 (*Sinosauropteryx lingyuanensis* sp. nov.) and IVPP V 14202 (*Huadanosaurus sinensis* gen. et sp. nov.) were discovered in Dawangzhangzi Village, Lingyuan County-level City, Chaoyang City, Liaoning Province, China. These sinosauropterygid specimens are deposited in the Institute of Vertebrate Paleontology and Paleoanthropology, Chinese Academy of Sciences.

The two new sinosauropterygid specimens have been recovered from the Dawangzhangzi bed of the Yixian Formation. The Yixian Formation was deposited during the second phase of the Jehol Biota [57]. Previous studies utilizing ^40^Ar/^39^Ar dating on the Yixian Formation suggested that the lowermost fossil-bearing horizon (Lujiatun bed) was older than 128 Ma [58], while the upper part of the Yixian Formation was ∼121 Ma [59,60]. The Jianshangou and Dawangzhangzi beds, which are the most fossil-rich horizons, were dated at ∼125 Ma and ∼123 Ma, respectively [61,62]. However, the high-precision geochronological studies by the U-Pb chemical abrasion-isotope dilution-isotope ratio mass spectrometry (CA-ID-IRMS) dating technique suggest that the entire fossiliferous horizons of the Yixian Formation span <1 Myr maximum [42]. Although U-Pb CA-ID-IRMS dating has not been used on the volcanic tuff layers from the Dawangzhangzi bed, it is inferred that the Dawangzhangzi bed was deposited contemporaneously with the Jianshangou bed ∼125 million years ago, given the remarkably brief duration of the fossil-rich horizons within the Yixian Formation.

### Phylogenetic analyses

In order to investigate the phylogenetic placement of *Sinosauropteryx lingyuanensis* sp. nov. and *Huadanosaurus sinensis* gen. et sp. nov., these two new species were included in the TWiG (Theropod Working Group) data matrix [13] and the Ontogenetic State Partitioning (OSP) matrix [14]. Some modifications to the state of the ‘compsognathids’ were made in the TWiG data matrix based on the observation on the ‘compsognathid’ specimens (Table S4). The datasets were analyzed using the Tree Analysis Using New Technology (TNT) version 1.5 [63]. The ‘maxtree’ was set at 10 000 trees in both analyses. During the analysis of the TWiG data matrix, we used the ‘New Technology’ search options, with ‘sectorial search’, ‘ratchet’, ‘tree drift’ and ‘tree fusion’, recovering a minimum tree length in ten replicates to search the most parsimonious tree. The tree search strategy of the OSP matrix was to perform 100 New Technology replications followed by exploration of the sampled tree island using ‘Traditional Search’. Our phylogenetic analysis produced one reduced strict consensus tree (tree length = 3419) based on 73 most parsimonious trees with the TWiG data matrix (Fig. S5) and one strict consensus tree (tree length = 17 006) based on more than 10 000 most parsimonious trees with the OSP matrix (Fig. S6). The strict consensus tree from the TWiG data matrix is highly resolved among the early diverging coelurosaurs, but poorly resolved among the maniraptorans. The rogue taxa (*Hesperonychus, Pyroraptor, Epidendrosaurus* and *Kinnareemimus*) were identified by ‘prunnelsen’ and ‘pcrprune’ in TNT [64,65] and excluded from the reduced strict consensus tree.

## Supplementary Material

nwaf068_Supplemental_File

## Data Availability

All data analyzed in this paper, including the phylogenetic analyses, are available as part of the Supplementary Information of this paper.

## CODE AVAILABILITY

The phylogenetic datasets are included in the Supplementary Information.
