# Supplementary material for: Two new compsognathid-like theropods show diversified predation strategies in theropod dinosaurs
Source: Natl Sci Rev. 2025 Feb 22;12(5):nwaf068. doi: 10.1093/nsr/nwaf068 (PMC11970238; doi:10.1093/nsr/nwaf068)
Supplement: nwaf068_Supplemental_File [file nwaf068_supplemental_file.pdf]

Supplementary Materials for

Two New Compsognathid-like Theropods Show Diversified Predation Strategies in Theropod dinosaurs

Rui Qiu[1,2,3], Xiaolin Wang[1,3,*], Shunxing Jiang[1,3], Jin Meng[4,5]& Zhonghe Zhou[1, 3, *]

[1] Key Laboratory of Vertebrate Evolution and Human Origins of Chinese Academy of Sciences, Institute of Vertebrate Paleontology and Paleoanthropology, Chinese Academy of Sciences, Beijing 100044, China.

[2] Natural History Museum of China, Beijing, 100050, China.

[3] College of Earth and Planetary Sciences, University of Chinese Academy of Sciences, Beijing 100049, China.

[4] Division of Paleontology, American Museum of Natural History, New York, NY 10024, USA.

[5] Earth and Environmental Sciences, Graduate Center, City University of New York, New York, NY 10016, USA.

Corresponding author: Xiaolin Wang and Zhonghe Zhou

e-mail: wangxiaolin@ivpp.ac.cn, zhouzhonghe@ivpp.ac.cn

The PDF file includes:

Supplementary Text

Figs. S1 to S7

Tables S1 to S4

References

Supplementary Text

Expanded description and systematic paleontology of *Sinosauropteryx lingyuanensis* sp. nov.

The anteroposterior length of the premaxillary body is relatively short, approximately 6% of the length of the ventral side of the skull, and is shorter than that of most theropods. The anterior margin of the premaxillary body is nearly straight and almost perpendicular to the ventral margin. As in other compsognathids, the maxillary process of the premaxilla contacts the nasal and separates the maxilla from the margin of the external naris. The maxillary process extends to the anterior border of the antorbital fossa, differing from that in *Huaxiagnathus*. The dorsal margin of the maxilla contacts the lacrimal, excluding the nasal from the composition of the antorbital fossa. The jugal ramus is long and slender, approximately 3.73 times longer than the length of the ventral margin of the anterior body of the maxilla, a ratio larger than that of any compsognathid-like theropods except *Compsognathus*. The “L”-shaped lacrimal bears a slender and extremely long anterior process that reaches the anterior margin of the antorbital fenestra, forming the dorsal margin of this fenestra. The dorsal margin of the dentary extends further anteriorly than the ventral side, as in *Sinosauropteryx prima* and *Juravenator* (Currie and Chen, 2001; Chiappe and Göhlich, 2010). As in other sinosauropterygids, there is no external mandibular fenestra. All the teeth are straight along two-thirds of their height and slightly curve posteriorly at the tip. There are at least four premaxillary teeth, which are spaced apart, unlike the closely packed teeth from the maxilla and dentary.

The lateral surfaces of the postaxial cervicals are exposed. The postzygapophyses of the anterior cervicals are subequal in length to the prezygapophyses, extending further than the posterior articular faces, which contrasts with the short postzygapophyses in other compsognathid-like theropods, except for *Scipionyx prima* (Dal Sasso and Maganuco, 2011). In the

3rd and 4th cervicals, the posterior articular surfaces extend more ventrally than the anterior articular surfaces, creating an angled shape. This angling is rarely seen in sinosauropterygids, but present in dromaeosaurids (Ostrom, 1969). While some cervicals of *Sinosauropteryx* also display angling, it is not as pronounced as in the new specimen (Currie and Chen, 2001). The ventral margin of the centrum is straight, different from the concave ventral margin in *Sinosauropteryx prima* (Currie and Chen, 2001). As in other compsognathid-like theropods, the cervical ribs are approximately twice the length of the corresponding centra (Ostrom, 1978; Dal Sasso and Maganuco, 2011). Although all dorsals are incomplete, we can observe that the neural spine is fan-shaped dorsally, as in other compsognathid-like theropods (Sale et al., 2014). As in *Sinosauropteryx prima* (Chen et al., 1998), the number of caudal vertebrae exceeds 60. The anterior caudals bear an accessory neural spine at the base of anterior margin of neural spine, as in *Sinosauropteryx prima* (Currie and Chen, 2001). In the middle caudals, the neural spine is located at the posterior half of the centra and lacks an accessory neural spine. The neural spine is absent from the 16th caudal vertebrate. The centrum is longer than its height from the 14th caudal. The chevrons expand at their distal end and curve posteroventrally. Approximately 15 rows of gastralia can be observed between the forelimb and the pubis. The anterior segments are stronger than the posterior segments, with a similar width to the dorsal ribs at their midline. Each segment on one side consists of a straight lateral gastralia and a bowed medial gastralia. The lateral gastralia is shorter than its medial counterpart.

Only the proximal end of the right scapula is preserved. The triangular acromion is not as prominent as in *Huaxiagnathus* and *Sinocalliopteryx*, where the prominent acromion is as wide as the scapula shaft (Hwang et al., 2004; Ji et al., 2007a). The coracoid is semicircular, and its postoventral process is stout. The delpectoral crest is approximately half the length of the humerus, a ratio similar to that of *Sinosauropteryx prima* (Chen et al., 1998), larger than in other compsognathid-like theropods (Ostrom, 1978; Hwang et al., 2004; Peyer, 2006;

Ji et al., 2007a; Chiappe and Göhlich, 2010). The internal tuberosity is small, and the humeral shaft is slightly slender than in *Sinosauropteryx prima*. The ulna bears a robust olecranon. The ungual phalanx of digit I is slightly shorter than the humerus and not strongly curved. The flexor tubercle of ungual I-2 is particularly strong and approximately subequal in length to the articular facet.

The preacetabular process appears squared in lateral view, contrasting with *Sinosauropteryx prima*, where the anteroventral corner forms a prominent hook. The preacetabular process is as wide as postacetabular process. The pubis points anteroventrally, as seen in *Sinosauropteryx prima*, *Huaxiagnathus*, *Compsognathus* and *Mirischia* (Matrill et al., 2000; Currie and Chen, 2001; Hwang et al., 2004; Peyer, 2006). As in other compsognathid-like theropods, the pubis bears a very development pubic boot, with a significantly stronger posterior process compared to the anterior process (Sale et al., 2014). The nearly triangular obturator process is located at the proximal end of ischium, as in *Sinocalliopteryx prima* and *Mirischia* (Currie and Chen, 2001; Matrill et al., 2000). The tip of obturator process slightly curves dorsally. Unlike other compsognathid-like theropods (Currie and Chen, 2001; Hwang et al., 2004; Peyer, 2006; Ji et al., 2007a), no prominent ischial boot is present at the distal end of ischium.

The bowed femur is approximately 90% of the length of the tibia. A popliteal fossa separates the lateral and medial condyles at the distal end. The tibia is long and slender, with the cnemial crest extending only a short distance. The distal tarsals remain fused with the metatarsals. Metatarsal II~IV are subequal in length and width, approximately about 60% of the length of the tibia. Metatarsal V is short and splint-like, approximately one quarter of the length of metatarsal III. The third digit is the longest, followed by the second and forth. The length of digits decreases gradually from proximal to the distal end. All pedal ungulas are small and weakly curved.

Expanded description and systematic paleontology of *Huadanosaurus*

***sinensis* gen. et sp. nov.**

The skull is subequal to the length of femur. The anteroposterior length of premaxilla is greater than its dorsoventral height, similar to other sinosauropterygids and *Ornitholestes* (Osborn, 1916). The prenarial region is significantly shorter than the subnarial region. Both the maxillary and nasal processes exhibit a similar posterior extension. The palatal process points posteroventrally, differing from the posteriorly pointed process in *Sinraptor* (Currie and Zhao, 1993), *Kilekus* (Averianov et al., 2010), *Compsognathus* (Peyer, 2006), or a posterodorsally pointed process in *Allosaurus* (Madasen, 1976) and *Zuolong* (Choiniere et al., 2010). In contrast to the pointed end in other theropods (Madsen et al., 2000; Sampson and Witmer, 2007), the distal end of the palatal process is stout, with a small foramen near the distal margin. The similar condition of palatal process is only possessed by *Sinosauropteryx* (NIGP 127586), although in this specimen, the process is positioned lower and the fenestra is located above the alveolar margin. A circular subnarial foramen is located in the lower half of the posterior margin of the premaxillary body, unlike other theropods where it is typically positioned higher. The ratio between the anteroposterior lengths and the dorsoventral height (at highest point) is approximately 1.72. The anterior process is confluent with the ascending process as *Sinocalliopteryx* (Ji et al., 2007a) and *Scipionyx* (Dal Sasso and Maganuco, 2011). There is no concavity on the anterior margin dividing the anterior and ascending processes. Unlike *Compsognathus, Huaxiagnathus, Juravenator, Sinosauropteryx, Proceratosaurus*, there is no concavity on the anterior margin separating the anterior from the ascending process. A large oval fossa is present between the anterior margin of the maxilla and the antorbital fossa. The antorbital fossa occupying most of the lateral surface of the maxilla. The slit-like promaxillary fenestra is located at the anterodorsal corner of the antorbital fossa, similar to *Zuolong* (Choiniere et al., 2010) and some tyrannosauroids (Rauhut et al., 2010; Brusatte et al., 2009; Sereno et al., 2009; Tsuihiji, 2011). The promaxillary fenestra is absent in other compsognathid-like

theropods except for *Compsognathus*, whose promaxillary fenestra is relatively large and oval. The round maxillary fenestra is more than one-third the height of the antorbital fossa, similar in size and shape to that of *Compsognathus*. The maxillary fenestra is extremely small in *Juravenator*, *Sinocalliopteryx*, *Scipionyx*, *Huaxiagnathus*, while *Sinosauropteryx* possesses a large rectangular maxillary fenestra. The jugal ramus is approximately 1.37 times longer than the anterior body, a ratio lower than *Juravenator* (2.02), *Compsognathus* (1.7) and *Scipionyx* (2.37), but larger than *Sinosauropteryx* (0.53), *Sinocalliopteryx* (0.78), and other basal coelurosaurus. The pneumatic foramen is present on the surface of the nasal. The subnasal process is not preserved, making it difficult to determine whether the maxilla contributes to the margin of the naris. The lacrimal is "L"- shaped, with the anterior process slightly shorter than the ascending process. The distal end of the anterior process is pointed and does not reach the anterior margin of antorbital fossa. A trianglular pneumatic foramen is located at the junction of the anterior process and ascending process. The pneumatic foramen extends up to approximately one third of the anterior process, and leads to a groove anterior to the pneumatic foramen, indicating the presence of lacrimal sinus as in *Alioramus* (Gold et al., 2013). The foramen is absent in other compsognathid-like theropods except *Juravenator* (Currie and Chen, 2001; Peyer, 2006; Dal Sasso and Maganuco, 2011). However, in *Juravenator*, the pneumatic foramen is only a small concavity (Chiappe and Göhlich, 2010). The posterior process is only a quarter of the length of anterior process. The dentary is long and slender as in other sinosauropterygids. The dorsal and ventral margin of dentary run subparallel, although the ventral margin curves dorsally at its anterior end, resembling the condition seen in *Sinocalliopteryx* (Ji et al., 2007a). On the posterior margin of the dentary, three processes are present: a small dorsal process, a large middle process, a ventral process that is less than half the length of the middle process. The middle process and the ventral process are separated by a "U"-shaped concavity, suggesting the presence of a small crescent-shaped external

mandibular fenestra, which is absent in other compsognathid-like theropods (Ostrom, 1969; Currie et al., 2001; Hwang et al., 2004; Ji et al., 2007a; Dal Sasso and Maganuco, 2011). The external mandibular fenestra is only approximately 5.74% of length of the mandible, smaller than in any other theropods possessing this fenestra. On the lateral surface of dentary there is a thin groove with elongated neurovascular foramina extending anteriorly, similar to the condition in the primitive tyrannosaurids and some non-coelosaurid theropods (Currie and Zhao, 1993; Sereno et al., 1998; Schmerge and Rothschild, 2016). Among compsognathid-like theropods, *Compsognathus* has four rows of neurovascular foramina, while *Huaxiagnathus* and *Scipionyx* have only two rows. Only the dentary of *Sinosauropteryx* bears a single groove, but the neurovascular foramina are relatively large and circle in shape. The surangular is nearly half the length of the mandibule, with the anterior surangular foramen positioned near the dorsal margin of anterior end, transitioning into a narrow groove as in *Proceratosaurus* (Rauhut et al., 2010). The angular is strap-like. The crowns of all preserved premaxillary teeth are straight, with both mesial and distal carina lacking serrations, as in *Sinosauropteryx* and *Compsognathus* (Currie and Chen, 2001; Peyer, 2006). Ten maxilliary teeth are preserved, with the estimated total number not exceeding 12. This count is similar to most coelurosaurs (Osborn, 1916; Xu et al., 2004, 2006; Choiniere et al., 2010; Chiappe and Göhlich, 2010; Azuma, 2016) except for *Compsognathus* (Peyer, 2006), *Proceratosaurus* (Rauhut et al., 2010), and *Kileskus* (Averianov et al., 2010). The dentition only extends slightly beyond the anterior margin of the antorbital fenestra over a short distance, contrasting with most theropods where the dentition extends significantly beyond this margin. The cross section of the first two maxillary teeth is incrassate, while the crown of other maxillary teeth are laterally compressed with labial depression sensu on the labial surface near the root. The denticular density per milimetre is 10 in the sixth and seventh teeth. The distal carina is strongly labially deflected, resulting in an asymmetrical crown in

distal view, as in tyrannosauroids (Bakker et al., 1988; Carr and Williamson, 2004; Smith, 2005; Brusatte et al., 2012) and dromaeosaurids (Currie et al., 1990; Hendrickx and Mateus, 2019).

All cervical vertebrae, except for the atlas, are preserved. The postzygapophysis of the axis is longer than the prezygapophysis. The epipophyses is development, extending posteriorly beyond the postzygapophyseal facets. The anteroposterior length of the axial neural spine is greater than its height. The axial centrum is strongly angled in the lateral view, different from a rectangular centrum in other compsognathid-like theropods (Ostrom, 1978; Currie and Chen, 2001; Dal Sasso and Maganuco, 2011). There are no pleurocoels on the lateral surface of the axial centrum as in *Scipionyx* (Dal Sasso and Maganuco, 2011). The middle cervical vertebrae (from the 3rd to 7th cervical) share the similar shape. The prezygapophysis is elongated, surpassing the length of the postzygapophysis. The epipophysis is mound-shaped and does not extends beyond the postzygapophyseal facets. The diapophysis is short. The prezygapophyseal paradiapophyseal fossa is presented between the prezygapophysis and the diapophysis. From the 4th cervical vertebra onwards, the neural spine is taller than its anteroposterior length. In the lateral view, the shape of the centrum of the middle cervical vertebrate is similar to that of the axis. The centrum of the 7th cervical vertebrate is the longest, approximately 1.9 time longer than the axial centrum. The ratio between the longest cervical vertebrate and the axis is less than 1.5 in most coelosaurus, such as 1.13 in *Scipionyx* (Dal Sasso and Maganuco, 2011), 1.46 in *Compsognathus* (Ostrom, 1978), 1.33 in *Albertosaurus* (Russel, 1970), 1.15 in *Bambiraptor* (Burnham, 2004), 1.31 in *Neimongosaurus* (Xu et al., 2001). This ratio is close to *Alioramus* (2.08, Brusatte et al., 2012) and some ornithomimosaurs (1.75 in *Deinocheirus*, Lee et al., 2014; 2 in *Gallimimus*, Osmólska et al., 1972). The posterior cervical vertebrae possess higher neural arch and shortened prezygapophysis. The infradiapophyseal fossa is visible on the 10th cervical vertebrate. The centrum of the posterior cervical vertebrae are

rectangular in the lateral view, with pleurocoels and parapophyses present on all post-axial cervical vertebrate. All 13 dorsal vertebrae are preserved. The neural arch of the first two dorsal vertebrae is as high as the centrum. On the first dorsal vertebrate, a triangular infradiapophyseal fossa is present beneath the diapophysis, separated from a large infraprezygapophyseal fossa by a thick anterior centrodiapophyseal lamina, and from a small infrapostzygapophyseal fossa by a thick posterior centrodiapophyseal lamina respectively. In the subsequent dorsal vertebrae, these two laminae become thinner. As in other compsognathid-like theropods, the neural spine possesses an anteroposterior at its distal end in the lateral view (Currie et al., 2001; Naish et al., 2004; Peyer, 2006). The diapophyses of the middle dorsal vertebrae show a constriction at their base, and the centrum of all dorsal vertebrae are rectangular in the lateral view. An oval pleurocoel are present on the lateral surface of the first two dorsal vertebrae, but absent in the last dorsal vertebrate. This contrasts with other compsognathid-like theropods where pleurocoels are absent on all dorsal vertebrae (Currie and Chen, 2001; Naish et al., 2004; Peyer, 2006; Dal Sasso and Maganuco, 2011). The centra of the preserved caudal vertebrae are similar in length. All preserved neural spines of caudal vertebrae are stout, except for the 7th caudal vertebrae, which has a blade-like neural spine as the neural spines of dorsal vertebrae. The distal end of caudal neural spine possesses a fan-shaped expansion. The long and slender scapula is strap-like, with a ratio of 12.67 between its anteroposterior length and narrowest width. The anteroposterior length of the acromion process is slightly greater than its dorsoventral height. A small concavity is located just behind the posterior margin of the glenoid fossa, with a coarse surface that may serve as an attachment site for the M. triceps brachii caput scapulare (Jasinoski et al., 2006). The distal end of the scapula possesses a slight expansion. Different from a subcircular coracoid in other non-maniraptoran theropods (Currie and Chen, 2001; Brochu, 2003; Peyer, 2006; Dal Sasso and Maganuco, 2011), the coracoid is strongly elongated lateromeidally, with the posteroventral process

showing a further extension. The small coracoid foramen is located near the scapular articular surface. The furcula is “U-shaped”, with the angle between two rami measuring 144°, which is larger than in other compsognathid-like theropods and other primitive coelosaurs (Makovicky and Currie, 1998; Hwang et al., 2004; Dal Sasso and Maganuco, 2011). The ramus of furcula is straight, with blunt epicleideal facet. As in other compsognathid-like theropods (Ostrom, 1978; Currie et al., 2001), the forelimb is relatively short, approximately 36.8% of the length of the hindlimb. The humerus is S-shaped in lateral view. The deltopectoral crest extends more than one-third of the shaft. The internal tuberosity is small and mound-like. A deep groove divides the lateral and medial condyles on both the anterior and posterior surfaces. The ulna is less than 70% of the length of the humerus. The shaft is straight. The olecranon process is development. The coronoid process is relatively small, about half the height of the olecranon process. The lateral tuberosity is very weak. The radius is about 64.2% of the length of the humerus, with a stout distal end showing a slight expansion. Metacarpal I is less than half the length of metacarpal II. Metacarpal II is the longest and strongest metacarpal, approximately 37.7% of the length of the humerus, a ratio smaller than observed in other sinosauropterygids. The ligament fossa is small and oval, dorsally located at the distal end. Metacarpal III is approximately 37.7% of the length of metacarpal II, less than half of its width. Phalanx I-1 is the longest and strongest phalanx, slightly surpassing the length of metacarpal II and exhibiting greater strength than the radius, as in other compsognathid-like theropods (Currie and Chen, 2001; Peyer, 2006; Chiappe and Göhlich, 2010; Dal Sasso and Maganuco, 2011). Phalanx II-1 is about 1.5 times longer than II-2. Only two scattered non-ungual phalanges of the third digit are preserved. A short phalanx is speculated to be either phalanx III-1 or III-2, it being shorter than phalanx III-3. All unguals are strongly curved. Phalanx I-2 is the largest ungula. Phalanx III-4 is the smallest ungula but possesses a development flexor tubercle.

The ilium is shorter than the femur. The preacetabular process is subequal

to the length of the postacetabular process. A deep and long cuppedicus fossa is present on the ventral margin of the preacetabular process. The supracetabular crest is very development, contrasting with the extremely weak or nearly absent supracetabular crest in other compsognathid-like theropods (Currie and Chen, 2001; Ostrom, 1978; Dal Sasso and Maganuco, 2011) except *Mirischia* (Naish et al., 2004). Above the supracetabular crest, there is a longitudinal ridge that divides the lateral surface into two sections. This feature was regarded as a synapomorphy of tyrannosauroids (Holtz, 2004). The pubis is approximately 86% of the femoral length. The pubic shaft is straight and possesses a vertical orientation. The pubic boot possesses a small anterior process and a large posterior process. The angle between the pubic boot and shaft is 75°, different from the right angle in *Sinosauropteryx* and *Mirischia* (Currie and Chen, 2001; Dal Sasso and Maganuco, 2011). The ischiadic shaft is nearly straight, with a weak concave on the dorsal margin. The base of the obturator process occupies more than 70% of the total length of the ischiadic shaft. The distal end of obturator process points anterodorsally. There is no distinct femoral neck between the femoral head and shaft. The lesser trochanter is separated from the great trochanter by a deep groove. The lateral surface of the lesser trochanter is convex, as in other non-pravians coelosaurus (Choiniere et al., 2010; Brusatte et al., 2012; Balanoff and Norell, 2012). No accessory trochanter present distal to the lesser trochanter, different from *Mirischia* (Matrill et al., 2000) and *Juravenator* (Chiappe and Göhlich, 2010). The tibia is about 1.28 times longer than the femur. This ratio is larger than other compsognathid-like theropods (Ostrom, 1978; Currie and Chen, 2001; Hwang et al., 2004; Ji et al., 2007). At the proximal end of the fibula, there is a prominent anteroposterior expansion with a concave medial surface.

Phylogenetic Analysis

Modified Data matrix from Brusatte et al., 2014

Allosaurus_fragilis ?10000?0000000100011001000101200111011001 0??000

000000000100000000001000100000000000101010010000000100101000000
0000001000000000????000000000000000?10000000000010000100101000
000100010000011000000100000000000000000000100000000000?0?000?00
11000010000001000011100021011000000000200000000?0000??0000000
?00000??000000010000[01]0?0????????0000020?00?0000000101000?0?
0010000?00000000000001000010?01000??0000000?000?00000200000000
0000?00000?00?001000000000020000?000100??001100000[01]0?000000001
000000001100000000001000110000000211210010010001 00?1????000000
000100??10?0000?00000000000011000?100?000?0?1000000000????000?
00010010000101000000000000000102000000000000000000001000000000 0
020101[01]0000100000000000000000010000001 0?00010000010201100000
000011000000000100000000000000100100010000000000000000000000000
000000000000000000000??0000000000000000000000?0000010000000000
00000?
Sinraptor_dongi ?10000?000?000100110000000101200001011001 0?00?00
0000000010000?00000100?10?000000000101010010000000100101000000
000001??????0??1?10?0?0??00??0??????000000000?010??01001010000
001?0?100000110000001000000000000000?000?000?00?000000?0?000?001
10000100100010000010000 2101???000000000000000000?0000??0000000?
00000??000000010000??00?00??00?????2????????????1010????????
??????????????????????????1??????0??000??000000002000000000000
0000000?00?00??0???00020000?0001?0??0011000?????????000100000
0000000000000100000110000000210210 0000?010?00?100000000000010
0??01?0000?00000000000?11000?100?010?0?100?00?001000?001000010
0100001010?0?00000000000112000000?00???00000010000?0000002010
1??0??1000000????????????00001001 0?0000?0000102011 01010?000110
00000??1?00000000000001001000??0???00000000100?0000000000000 0
???????00000000??0000000000000000000000000?0010?00000?00000001
Dromaeosaurus_albertensis ?0??0010000000000 10??0???0?01??01110???
?1111????101?1001100??0000001001111000?000010 1001????????????

???1?????????
?????????????????????????????????11?1?0????00??????00?0?00??1?
????????1?00??011000100?0??????????????0???0000?00000??0000??
0000000?00000??00???
?????????????????????????????????00????????????????????????
?????????????0????0?????????010121?000??0???00?0?????????0???
?0??0??0???0??????0?0100?00?00???????????????????????0????000
00100000???????0???????0001001?0?0????00??00?????0?0?10???????
????000?100000001 0????00000000001120100000000?0?00010????????
???
???0??000?0?????????????0??0?010??????????????????????020000
0???????????????????????????????????0?1?0??110???????????????
?000?0?
Deinonychus_antirrhopus ?0010????1???????1??0000?0111000111000111111100?1??00001110100000?0100111?1000?0100101000?110001100?1121011????0011011112 1??????1?1101110010011100000100221110101020220?01111211201?1111000000000100011010010000000001100000?00010?0010002110000011110110000??000001220000000 0?0????000????0???????????00000??000000000000?[01]0?0?????????????20000?000?000111000?0?0010000?0000000000000101?0???00000??0100000?000?00000000000000000000000000?0010000000 0????????0?1????00111?????????0000?????00001?10?0010001?000001010??00?0???????00??0?0??0?????00001000000??01?00?0?00000001??????????????????????0?0?1001000?000?00?????????????????0000000000112010 0?0000?0?????00000000?00001000100?????00000?110101000010 0??1?0000????????????????00000?000000000?00?100?00??0001?1??0?000010?10000000001 0?010??00?00000000000000000000000??0????000000000100??1101?000110???200?10001?0
Velociraptor_mongoliensis ?001001001000012011200001011100011100012111?10?01010000111010000000100111110001010010100001100011001112 1

01111100011011112111101011111111001001110000010022111011102022020
11112212011111?00000000011101101001000000000110000000011?00101
0211000001110011000000000000112000000000000?000000?0000??000000
0??0000??0000?00000000[01]0?00??000?00000020000?000?00011100?0
?001000???00000000000010100???00000??0100000?0000000000000000
0000000000000?0110000000010[12]21?000110110001110000?10000000
0?0100001?10?0010001?0?00010101000000???0??0?01??00?0????00000
1000000??01?0000?0000001001?0?0???000??00110100?0???01000?000
?000001001000?0000000000??00?112010000???????00000??????????
???01001000?000001110101?0?0?00?010000110?01?010000000000000
000000000?000100000?000?101010?00001001000000000010?01002000000
0000000000000000000??00001000000000010000?1?10?00110100200010
00100
Balaur_bondoc ???
??1?2??1??
??0???10?????????0????111[01]11101001?1?00001???2???0111?202?02?
0?1?22122????????????????2110001001000??00??1??????????0?00??
0??????????????????????1?????2?00??????????????????????????????
??????????????00000000?[01]???0????????????20???????0???0111000?
0?001000???010?00?????010?00???21??0?0000020?100??0000??????0
0???0?000000000?00??0?00000????????010?????????1????????00000??
??
??
??
??0100?0?0??0?00?1101010000?00?01000?0110?01??????????????000?0
0??00?????00100000?00??1??????0000100100?0000?????0?0?????????
??0000000000000000000??000????000000210????????000?10???200?1??
????
Tsaagan_mangas ?0010010010000120112000010101001?100012111110
0010110000100??000000100?1111000101001010000110??1??????0????

?????????????1????????11?111??????????????????????????????????
?????????????????????????????????????00??00??00?0000??????????
?211000001 1?00?0000000?00001????0000000????000000?0000??000000
0?00000???000??????????????????????????20000???0?0?0??10??????
??
????????????????0????????0121?00???0??00011?0?????????????00?01
00001?10?0010001?0?00010101000?00???0??0?01??00?0????000001000
000??0??0000?000000001001?000???000??001101?0?0???????????????0
001001000000000000000??00?112010000000????000000???00?00001???
????????????00?110??
??000?000?????????0?10?010?????10??????????????????02?0000000??
????????????????????????????????????000?1?1????110????0???000?0?
Bambiraptor_feinbergorum ?0010??001000012010[02]00?010111000?11000
12111?1000101?00011?0?0?000?0100?1111000?010010100??1110?1100?1
??100?0?1100[01]1011??2????010101101110?001?100000111?211?0101?
202202?111022100???1110000000000010001101?0000000000001100000000 1
1?0010?021100??11?110??0000?000000122000?00000?0??00000??0000?
?0000000?1?000??000000000100??0??0?0?0?0?000002000 0?00000?011?
000?0?0?1000??000000000?00010??????00000??0000000?000?00000000
0000000000000000?00?011?1000000000121?000110??00011101100?10000
00001010000 1?10?0110001?000001000100000 0???0??0??1??00?0????00
0001000000??01?0000?00000001??1?0?????000??001101?????????????
??????0?????????????????0?000??00?1120100??????????????0000000?0
0001000101 1000?000001110101000010?00100000110?01?010000000?000
00?000000000?0001?000??00001?10?0?100010?100????????????????0?
000?????00??00????000000??00001000000000100?0???10000110100200
?10001??
Tianyuraptor_ostromi ??01??????????????????0????111??????????????0??
1?????????????????????????????????0?0?0?1010??0???0??????????0?0?
0?1??01?0112????????1??00?011110?001?110000111 2?1??0?1102223?211

11022?0???1?110??????????00?11????000??0???1??00?0??01???1011?
0???0??????1?0??????????0????2?000?0?????????????????????????
????????????0??????????0?00?00????00000?0?00?000?0?0??????????
?????????????????????????????????????00?1?0????10??????????
????0?0????????20?101?????????????1???????????0000?10??000??1??
?????????????????????????0???????????????????????????????????
???
????????????????0??????????????????????????????0???????????????
????????????01?1?1???00??0?0????000????????????????0?????0?0???
00????0????0?0???0????????0???0?????0??0???????????????????0????
?0???0???????????????????????????0010?????????0?0????????????0?0???
Sinornithosaurus_millenii
0001??????0??????????00???1110????1000111100?1????1??0????????
00?00100??1????010100??100?????????1??1????????0???00?1??1??????
?01?1?11011110?0?????0000?00?201?01?112023?2?111022?2?1???1????
?????110001?1100?0?00010001??0000???01101111002000000111100?00?
00???000???20000?000?????0??000????0????00000??1000??00?00?0??
???0?0??0????????00?0?[12]0000???0?000100?00?0??010?0??00???????
?????????????0000??0000?0000??0?0?00?????000??0?0000?00?0???01
11?11000????????01??10000111?1100?1000000?01010000000?0000000
?0?00000001000200???0??0??1??00?0????0000000000000??00?0000?000
00001??1????0??000??001101??????????0?????????????????????????
001000?010112010000???????100?0???????????????????????????00111
01????00?000??????????0?01??????????????????0?0???00000??0??00000?
000?1??0?0?????10?????0?00?????0?0???0100??????0????00000????0
01?000?????0??000100?011?????0110????0???000???
Microraptor_zhaoianus
0???????????????????100???
?0??010?0?1????0?01000000?????001?1??01?2100?01???0110?1212111
?01?10111111010??111000011012111??0112023?2?110221201?11111??0

0?0001110111110?100??000?11?000????1010111100?0?0???1?110???0?0
0??0?00???20??0?000????????????????????????????0?0???????000010
00?[01]0?00?0000?001000200???0?000?01???10?0???10100?000??1?0?0
00?????????000???????0?00?0000000000????000?00??000000000??121
1111??00?021???011011???1?1?1100?1000000???????????????????????
???????0???
???
?00???1??????????????????????00?????????????00001????????01???1
?10000?000???0000??0?01??10?????0?00000?0???00????0????0?00??0
????1????0000???10??0?00???0?010???????0???0?00??000??0?000000
?000?10?000000010???????????0?1??0?????1???1??
Graciliraptor_lujiatunensis ???
???????????????????????????????????????020???1?????????????????
?????????0???11?12?????????????????01011?1100001???????????????
??????????????????????0??0?010?[01]11?1??0??0??00?????????????0
???????????????????00????????????????200???????????????????????
???
?0??????10?0???000??0????????????10?0000??00?000???????????????
????????????????????????????10????????????????????????????????0
00???
???
??0????????
?????????????????????????1????00?00????????????????????????????
0000????00??????100??0??0????????0?????????00?0?00?????01??????
????????????0???00?????????????????0?000?10????????0?0?????????
????????
Hesperonychus_elizabethae ???????????????????????????????????????
???
???21??010???
??????????221211??????????????????????1???????????1???????????0

??1??
???
???
???????????????????????????????????????0???????????????????????
???
???
???
???????????????????????????????????01000?0110?01???????????????
???????????????????0???0?????1?????????????????????????????????
????????????????????000??
????????
Pyroraptor_olympius ??
???0?
???????1????????????????????????001????????????????????????????
???????????????????????????01??1?????????0??????????????0??????
0??
???
????????????????????010??0?????????????????????????????????????
????????????????????0??
???
???
???
?????????0?0??????????0??
??0???????????????0?????????????????0??????????????????????????
???
??
Rahonavis_ostromi ???
?????????????????????????????????????0???????????????011121???1
?01?011112?12???????????0??11??011??????0?01111?10111120?3?2012
101?121??21110001000?011101101?0100????0??1?????0??10001011111?

??
?????????????0011000[01]0???????????????2????????????1100??????
???????????????????010100??????????????000000?000000000100000
000?00000000?001??01???0????????0?1?11?????1????????00????????
??
??
???1
00?1??0?0??0011??????0?????0?100000????01??10???0?0????00?0???
00???????????00??0?00?1????0000??0?0???????0???010???????????0
00?????????0000000?000?10??00?0001????????00?0?11?0??0?????????
Buitreraptor_gonzalozorum ?0010???????????????00001011?00?????????
?100?10??01?0?????????00??001??????????0210??100?0010111100111
?110???100011012[012]121???????0110111010?1?1??0??????11?101??1
2[02]23??01?1?1?120???11?0??0??1?011?0?1110000?????0??1????????
1101000110011000???0??????0000??0?00?10200?00?0???????????0???
????0?000101???0?????00000000000[01]0??????????01000100?0?0?00?
?0?10000?0?001100??00000100000001?100???????????????000????00?
??0?00??????????0?00?000?0010?00??????????????1????0010??1100?
0000???00?0?????????1?11?00?0???010?0101?0??0????00000??1100???
?????0000???0?0??0??0??????????????100?????000??00?0??????????
???????????0?????????????????0?000???0?1?2??????????????????000??
?????0001001000??0??00000111010100???0?001??000???????01000???
00??0?????????0?0?000???????????00????0?1000100?0????????01000??
???0?0001000000000????000000??0??01000?0??001????????10????111
00?00010001??
Neuquenraptor ??
???112111111
11???1????1?????????????0??1001????????????01111?10111202202011
?121202011?10000??0?010??111100000????0??11????0??110110?110???
??????????????????0?????220????????????????????????????????????

?????????0?00000?0????????????????1???????????1100?0?0?0011
000?00000???????????0??????????????00000?00000000000000000
?0000000?00??
???
???
???1101
1?0?????0011???01???????0010000110?01?010000000?00?0100???00?
?????????000?00?00?1????10????01???????00???010??????????????00
???0????000000??000010??0?000010?????????0??11001000???????
Austroraptor ?0000???????????????????2?1?0????????12?0001???
?????????????????0?011???????????210??10?????0011?001????11???
???????????????????????????00????????0???????????????????????
????????????1??????0?0?0?0??[12]1??????????0????0??????????????
??11?00??01??00??????????????1[12]20??0?0??????????????????????
???????????????0??00?00?????????????????????????????????????0?
0?001100???00??0000000??
???00?0
?????????0?10100?0000010??1???????1?????????????????????????
?????0??01?01????????????????????0?10?00????????????00000?000?
00?????????????????0??0010000?????????????????000???????0001
001101???????????????101???????????????????????????????000?????
???00?00????????????0?????0?0???????????????????????????0?0001
000?????0???????0000???????00??????0?????01?1????1?????1?0??00
01??
Shanag_ashile ???????????????????1??1010110????????????????
???????????????00??011??????????0100101l1???????????????????
???
??0????0??????????????
?21100001???00???????????????????00??0???????????????????????
???

??
??
???????1?10???00?0?000000?????????????????????????????????????
??
????????????????0??00??00????????????????????10???????????????
??
??0?0????????????????????0?????????????????????????????00?????
???????????????????????????????????????1?????????????????00????
Mahakala_omnogovae ???????????????101??????????????????????1?0
0???????0?0011??????????????????????02????1???00??0?11?011?211
??1?100011012210????????????????00??1110000?100?211101?2??????
????????????0111100????0?011001101?00000??00??11?0?????0000????
?00????????0????????????0????1[01]2?0??????0?????????????????0?
?1?00???????????0000010000[01]0????????????????????????????10??
[01]0???0?0000???0?????????????100????0??0???000000??0??001??0?
??000000000?00000000?001??00?000????1???0??????011?0????????00
0000???????????????????0???0??????????????????????????????????
?????????????????????????????????0?????000?00????????0????????
???????00010?????????????????????0????????????????????0000????
?0?01?000???????000??????????00????00100000???????0100000?0?00
0?100?0?000?0????0?0?0???0????1?10???00???????????0????010?20?
???0?0??0???????000000000??0??0100000000010?0????1?000???10???
?01????0?
Atrociraptor_marshalli ???????????????????[02]00?0??1010????????????
????????????????????00??010????????0?000101001????????????????
??
???0????0???????????
?????21?00??0??111?????????1???????????0??????????????????????
????????0????0??
??

??
????00001?1????1???0?0?000?01?????????????????????????????????
??
????????????????????0?????0?00????????????????????0???????????
??
??????0?0?????????????0??????0????????????????????????????000?
???????????????????????????????????????0??1??????????????????0
0????

Utahraptor ???????????????????[02]00?0??????????0?001??????
????????????????????????????????0????0101??1????0?1100?1??1???
?????0??011?????????????101?1???????????0?????????0?01????????
????11?????0111?00??0?001000100???10????????11??0???????0?????
0???????????1???0???????0???10???0???0????????????????????????
????????????????00000???????????????????20000?000000??????????
???00???????0??000000000
0000?0000?????0?10??????????????????????????????????????00????
?000001??????????????????????????1???0??00010?0???????????????
??
??
???01???????
??0?0?????????????0?????0????????????????00????????????0??????
?????????????????????????0?0???????????????????1??????????????
??

Adasaurus_mongoliensis ?0010?????0????2??????????????0?????0?1??
11?1?0?101??001??0?????0????0?1???0?????????????01100?11?0111?
10?111[01]100??011?1?1??????1?111111??????????????1022111010102?
2202?1?11221001?111000000?000?11010010010??00??00110?0?0?0001?
?00100?????????11????????00?0????1??????????0?????????00??00??
?000?0???????????000?0010000[01]0???????????????200?0???00??011
10???00?0000000000

0000000000000000?002??11?00???00???21?000??0??0001?1?????????0
???0??0??????????????????????????????????0?????????1??00??????0
000010??000??0??00?0?0?00????????0??????????0????????????????
???????0??????????????????0???????11201??????????????????????
???????????????????????????????????100000??0?01?????????????
??0?0???00?0????????000?00????10??????????????????????00???
???0??00????????????000000??000010000??00010??????????01???????
?????????
Achillobator_giganticus ????????????????????????01?1?????????????
???????????????????????????????????0000101??????0?01100?11210
???????0??011?11??????????101????????????00??10220??1101010210
2?011011?001?21110?0????00?000?101????0???????1???0????001??00
100021100??0??100????????0??????????????????????????????????
?????????????????0000???????????????????20000?0?0?0?0??10???
???00???0?00000??000??
?????0?0000????????0???????????????0??????????0???????????00??
?????????00?001???0?0?000??1????????????????????????????????
??
??10????????????
??0100??????????????????????????100000??0101?0100000?1000?????
?????0??????????0??01??????1??0??0??????????????01??0??????????0?
?00??????????0000000???0000100????????1????????????????1?1??????0?
00??
Saurornitholestes_langsto ???
?111?????????????11????0??????????????????100101?00?1100011001112
1011011100?1011?1?1?????????????111?????????000111221?1?1?1?2?0?
????????????????11??0????00??001101??00???00?0?1??00?000010?0
01000?????????1110011??????0????1220????????0???????????????????
?0000?????????????0??????????????????????????????????0???00??1????
???0?????????????????

???????????????????0???00??????????0??????00111????????00?00?
??????????00?01??0?0?0?000?00????????????????????????????????
??????????????????????????????????000??00?1???0?000?????????
???????????????????0??0000000??????????????????0??????????
????1?0???
????????0??????0???????????????0???????????????1??01?????????
???0?????????00???????????????????????????11??????????????????
0????
Saurornithoides_mongolien ?00??1?1??1101???0?110001?1000?????????2?
????????????????1?010?100?0010??1????0001110101??????????1???0?
?1?????100??1???020
12020?010??10[01]1?11110????????????0???10?01??????0??1?0???????
????010??11000??0?1?01?0?????0?000??????0??0000????0?1000?0??0
???????????0000???0????00100?0?????????????????????????????????
???00?????????0?0?
???0000000000000?20?0210000000000201001?10???1110?1?????????
???00?0100000??2??11?0?0?1?0100010100??00???0??0?????0??0?????
????????00???0????????1???
?????000?????????????????0??????????????????0???????00000???????
???????0?????????0?0????????????????????????????1?010?????????
??00????00000????????00??00????0?0???00????????????1??????0??00
0?0?????0???????????????????0?00???????0?101?0?????????1???0???
???0?0?00
Zanabazar_junior ?00101?12?110100?001?000??100000????2022000?21?
?0??11100??????100?001???1????000111010100??????????????????1?
1000?1020?1???2????????
?????????????????????011????2????????????00??00???11????????0??
110000000100000????1??00???????00?000?????001??0?00???????????
??0000??0?00?????????0[01]0?????????????????????????????????????
??0001

0000??000??2????1??0???0000201001?11???1110?1?????????????00??
100000??2??11?0?0?1?0100010100?000???0??0?????0??0?????????10?
?000??00?0??????????????????????000?00011010??????????????????
000?00000????0000???000?001???????????0????00000??????????????
?????0??????????????????????????????????0?????????????0?0?????
???000?????????????0??????00??0?????????????????01?00?0000????
????????????????????????????0?00????100100???????????1????000?
0?

Xixiasaurus ???????????????????01000101110??????2?22??????
?????????????0?00??001????????000201??01?0????????????????????
?????????????????????????????????????00?1?????????????????????
???????????????????????????????????0???00?????????????????????
1?000000???01?0000????00???????000000???00????????????????????
????0???
????????????????????????????0??????0??????????????????????????
??????????????????0?0?????????????????????????????????00????11
0100??2?01100?0?1?01000101000000???0??0???????????????????????
??????????????????????????????000??0??????????????????????????
??????????????????010?000????????????????00000????????????????
??
?000??????0??????0????0?0??????????????0????????????0100??????
?????????0????????????????????????1?010??????1??????????000???

Byronosaurus_jaffei ?????101???101?1100110001011?0??????20220?????
?????1?100??????0000001??11????000211??01?0?0???????010121????
????0??02???
???????????1????0?0??0???????21??????????0????0????1??????????
?11000000?1?00????0??0?000??0???000000????????????0???????????
???0?0????0????0010???
??000?????
????????????????????????000200011??0????????1????????????00??1

00000??2?011?0?0?1?0100010100??00???0??0?????0??0????????????
00??????????????????????????0???????????0?????????????????0
0?000000????????0??00??00?????????0???????0?0000??????????1???
??
??000?????????????0????000????????????????????????00?0100?????
???????????????????????00?????????1?0?0?1????1??????????000?0?
Sinornithoides_youngi ?0??01?????????????1?000??1??0????00???2????
???1???????????????00??0010???????0001110?01??????11??001??????
?????0011?102121011???1?00?1?101?0???100000????21??01??02??30?
??11?0??001??1110?????0???1?000110001?0??0?0?1??00?0???0???001
0?0??00000????0??0??0???00???0?200000?0????????????????????00
?????1?00??000??????????[01]0?0?????????0??0?20??0?00000?0??1000
???0?1000???00?0??????????0??010?0000???000000??00??0??000????0
00000000000000020?0210000000????????101??????1?010100??1??000?
???????????????????????????????????0????????????????????????????
?????????????????????????????????????000??0????????????????????
??0?0?00?????????0
???????????????00?110101?000?000????????????????1??????0?????00
0???00?0???0???0?00??0??1????0?????100????0?0?????????0???000?0
00???00??10?000?????0??00???0000000010????0?1?0?0101????0????
0????
Mei_long ?0?????????0?????????11010?????0?11?002012100001?
1010??100??????00010010??1?0000?021???01?001101100011?2110?01
???0110222111?1?????111?11100?0?1?1000001?012111?1??1200302?111
0221?[01]?0111100?0101001000??110011?0?0000???00000??000100?1??
0?1?000000?001?0?0000?000001020000?000?????????00?0??0??00??01
01?????????0?0000010000[01]0?0?????????0100020?00?00000?0111000?0
?0000000?0000000?000?01?1?0???00000????00000?00000010000??0000
0000000?000021?01100000??0???20???1010??101100?1100?10?0000?11
0??0??0???????????0?1?01????0?????00????????????????0???????????

??00??????????????????1???0??????0????0????0?????????????????
??????????????????0?000?????????????0???????0?0000?????????0??
???????????????????1?1?0????????????????????????????0??????00?
??00?00?0??????0????0????1?0?000?1001??00?0001?0?010???0???0?0
000000000?000????00???0???00?000000100?????????01??????????0?0
???
Jinfengopteryx_elegans ?0??????????????????1?01???2000?????????1??0
0?0????1???100??????00?00??0??1??0?000?1???0???1??????????????
????????02????2?200???????1101110??????100000?????1???????????
???????2??0???????????0?????????????????0?0??00????00??????????
????01?000001???0??0?0??????0????200?0?000???????????????????
???????0??????0?000???0?0??0?0???????????????00?0???0?0?01???0?
???
???????????????????10?0000???????????0??11?01??????????????000?
110?????0?01??1????0?????????0?????00????????????????????????
????0????????????????????????????????0????0????0????????????????
????????????????????0??00??????????????????????????0??????????
???0?????????0????1??0????00????0??????????????????????0???????
????????0?0??00??????00????1?0???0?????????0?????????????????000
??????????????0?0???????00?????????001?0?????????????????????0
00???
Anchiornis_huxleyi
000100?????????????11010??11100?????0?2?000??1???11???????????
000??01??1?????00020???01??0?101???00?0??010?01???01101220??01
?????01111110000??100000010?21?10011020?202?1?1122121???1?1?00
0??1?01?000121100010?1000?1??00?0????????0?11?011000001???0??00
?????000???030000?000??????????0??????0????0??????0??????00000
?0000[01]0?0?????????0000020000?0?00??0?10000?????10?0???0??????
??????????10?01000??0?00?00??00?00000????????????00000?0?00??0
100000000????????00??1110?1?1?1100010??000?110??0??0?01?010?0?

0?1??101?00??0??00??????????????0?????????1???000??0???2?????
?????1?????????00????0????0?????????????????????????????????
?0?000??01?????????0???????0?0000?????????0???000??????000????
??1???00??0000100000??0?01?????????0?????0?0???00?00?0??100?00
??00???11?0?0000???????0??????????0????1000????0????0??0?00000
00???00?0???????00100???0???0?01??????????000???
Xiaotingia ?00??????????????????1?1???111000????0?2?????????
?11????????????001001??1???00??020???01??0??0???1?0?????10?01?
????1??????10??????01?01111?10???1000001011?1110?1102122?????10
2?12[01]??????????10????????1?010001010000?1??0?10??00???0010?0
11000??1???00??0?????000???22000??000????????????????????0000
?????????0??000?10000???0????????010002?????????????11?000?0?00
???0???0?0?0????0???????????0100??1?00000???0?0000????????????
???????0??0?02????10000????????00??1??????1?1100?10??000?110??
0??0?01??1????0?????0???0?????0???????????????0?????????1???0
0????????????????????????????0????0????0?????????????????????
???????????????0??00???1?????????0???????????00?????????0?????
??????????0011??????0???000010?000???????????????0???????0???0
0000?0??100?00??00?1??1?0???????0????0??????????0?????000?????
0???0??000?000?????0??????????0?100???0?????0101????0???000???
Aurornis ?0010???????????????0101???11100???000?1?0??????
???????????????00?001????????0?020????1??0????????0????????0?
???0??0?221?00??????01?01111??0???10000??001?011????0212202O??
10????????????????????????0??100000?000000?1??0??0??00????111?0
11?0000????0???000???0000???20000?000?????????????????????????
??0??????000??0???000?0??????????00???2??0????????0?0?010?????
???0?????????????????????10?0??00??0?00?00?0000000????????????
???0???0??????1??010000????????0???1?1?1??1??1000100?000?110??
0??0?01??10???0?1??101?001???000???????????????0?????????10??0
00??0??02???????00????????????00????0???????????????????????????

???????????????0???0???1?????????0??????????00???????????????
???????0???01???1???00??0000100000??????????????0???????0???0
0??0?0??100?00??00????1?0???0????????0??????????0?????00??????
0??????0?000000???????????????00100???????????0?????????00????
Eosinopteryx
00010???????????????1?01??????000????0?1?0?????0??1????????????
?00?001????????00020????1??0????????0?????????????0??0?231?10?
??????110111??0???100000?001?1?1??1?02002?20??10?????????????
??????????00?1??0001000000?1??0??0??00????011?0???00??????00?0
?00???0000???20000?0000???????????????????????????0??????0000?0
?10000?0?0????????00???2??0????0???0?1?010????????0???????????
?????????????0??00??0?00?00?000?000????????????????0?????00??01
???000??????????0???11??1??1??1??0??0?000?11???0??0???????????
?????????0?????00???????????????0?????????10??0???????????????
???????????????0????0???
0???0???1?????????0???????????0??????????????000??????0??0011?
?1???0???000?100000???????????????0?????0?0???00??0?0??100??0?
?0??1????0?0?0????????0??????????0????100??????0??????00000000
???????????????00100?????????0?01????0????0????
Troodon_formosus ???1?1112?1101000001???0?011?0??????20220000210
?0??01100????0?10??001??????????0111010100???1111001012111111?1
000?1020??11??????????????1?010????000010??????????0?0?2?20?11
001?0??01111000???00010000021??01????000??1?0??010???1??0??0?1
?00000?0?0????????00??????????????????????001??0?000?????0??1?1
?????????00???
??
??????????????0?????00000201001??0???1110?1?????????0000??????
??
??????????????????????0?????0000??00110????????????????????000
0001000??000000??0000000????????????????????00???????0?0??00??

???????????????????0?????????????????????????????????00000?000
??0????????00??0???0??0????00??01??0000?0?1??01?01?00?0???????
?????0?0?????????????????0?000?10?0?10?0?0?0?????????????0??0?
Sinovenator_changii ?0???0002?000011110010101?1110?011?02??2??0011
???1?10100????0?000?001??????0000011110?1?????11?1010001211 0?0
01000110222?1?????????110111???????100?0???0?211?010112[02]0302?
1110221201?11110000??10011000111??110?000000???000110001000110
011000??1??011??0000?00000??0??00000000000?000000?00?0??000001
0100[12]00??00000?0010000?????????????????20000??00?0101?10????
??00?1000000100000000
00000?0?00?020?0110?0000000002 0?010?10??1011000???????000000??
???0000?02?011??00?1?0100010?????0????????????????????????????
??????????????????????????0????????0?00????1??????????????????
?0????????0???00000??00???0????????????????????00?????????????
00????0??????00111?????????????0000?0??0?01???????1??0????0?0?
0???0?0???????????0?0?1?1??0?????100????????0????0??01?01?0?00
00?00??????0?00??0???0000?0000000001?1???0?????0??1??1?????000
?0?
EK_troodontid_IGM_100_44 ?????0012????????????????????????????
??????????????0???????????????1??11??????????1??????0?????????
???1?00000????????????
???????????????????????????????????01?1?001???00??????0?0??1??
??????0???
???????1?1??
??
??0?????????
000???
????????????????????????????????0?????????????????????????????
??
??

??0??????????1??
??0????????????0000????????????????????0?1????????????????????
?????????
IGM_100_1126 ?0????002?0000?11[12]01?010??2000?011?02012100
0110?01010100??0??000000000?11?000000211??00?0????????????????
??0????01102???????????????????????0??1000001???211?010?020020
200010131012??111000????0??2?001210001?00000011?00?0?020000?00
100?1?00000111?01?0?00?000003??????000000?????00??00?00?0??0??
0000?00?00???0?0?????????0????????????????????????????????????
????????????????????????0?00???0000???0100000?00??0???0?00?00?
????00?1?000?20?02????0000001020?111??0??101?001???????0?000??
??
??
??
??
??
??
????
IGM_100_1323 ?00???????0???11???1?000??20000?1??020121?00?
1??0?0??????00?0?000?0????1??0000021???0000???????????????????
?????011???????????????????????????????????00??1??????020?2020
0[12]102?1011???????00???0????0??2???01??00??0????0?0???00?0?00
100?1?000000?1?01?0?00?00?003??????0000000????00?000?00?0?????
0000?0?000??0?00??
???00?????00?00?0?00??
???????1000??2????????????????????????????01??????????????????
??
??
??
??

??
??
????

Archaeopteryx_lithographi

100?0000??000??112010010??1110?011000012100?10?0000??100111?0?0
00001000002?00000200??00100?1?1???00?0??1?0?0???0021012311000?
???1011111111000?11000000112111010?12003020121022?2?2?111100000
?0001000000030?000000001100000??00?00011001110000100 1?0100000
0???0000?030000?0000000?00110??00?0??000?00??000???00?00000000
0[01]000?0????????00000?0000?000000010000000?0011000?000??000?0
000100000??00000?000000000000?0?00000000?00?0?[01]1000000?00???
000001000010 20?0000101110 1?0101100?1000000110?00000?01?01?000
0?0?0100?10100?000???0000?01??00?0????00???10??000??????????00
0000?0?0????????000??0011000?????0??????????000000011??????000
00?000000000??00???0???0???0?0100???????000?0000????????0?0011
101?10000?000?100000??0?01?01??????0????00?0???00?00?00?100000
?000?1?1000?0?0010010??000000?0?010020?1000?000000??0?000000?0
000?000?????0?0000100001001???0111????00?100010?

Confuciusornis_sanctus

10010???????????????1?000?00??0001???0??2??0??0??00??01???????0
00010000?10?0001?1??????????0???????????1021?0?2???0?2????4??111
?11010??13111000?1111000000 1121?1?1??12000?2?111023?2??12??10??
11??12021101 0030?0000000001102000??00?0101??11?10001100 1??????
0?????000?123001[01]00120??????????10??00??011200??01[12]01111100
000011000010010001 20000?00?0000?0??0??010000000?000211??001000
000001010210101 1[01]?10?00000010100000000010101001000110000 1100
0?0000000[01]000???200000110 1110?00??1100?10?0000?110?00000??1?
00?0000?0?01?0?10100?00???????????1??00?0????0000000??000??01?0
2???00100000?0?????????000??00???0?????????????????????????????
???????00010???0?0??00??10??????????????0????????0001?000???0????

0?00111?0?1?000?100??????0??0??1?????????0????00?0???00?00?0??
100000??0??1??0?????0?????0??00???????010???????0?????0?0000?0
?0??????0??0??????0?0000100?0?0?????01????????????1????
Jeholornis_prima
10????0????????1???0???0?????001??????????????????0???????????
0000?1???00?00?1?1?????????0????????0????1?0?11???02111031??01
?0???01103111000 1??1000001011?1??0??01?0???2???1?221?10???????
01????01011010030000000 00??0??1000??00?010????1???000?1?????0?
??0??0??0??12300?10010??????????????????0????00?00000??00?0000
00100000?0??001???0000?00000?0?10001200010 00?0010000?000?0000?
0000100001011000 0?000000000000?00000?010000000001000001000?000
00001100????????011?1110???1?1100?10?0000?110?00000??????????0?
0???????0??????????????????????0??????????0??00???????????????
??????????????000??00???0
??00???0??????????????????????????????0?0??0000???????0?001110
1?1?0???000???0000??0?01?????????0????00?0???00?0??0??100000?0
0??1?10???0?00???????00???????010???????0?????0???0??0?000?00?
??000?????0?0000100???0?????0??????????1?1?1??
Jixiangornis_orientalis
100??????????????????0010?????001????0?0??00000??????0100??????
000001????0?0??1??0????????00?0???1?00????1??20???02??1031??11
?1?00011031110001101000001111210101021200002010 1?221?10?2?11?00
1??1?01[12]1101003000?00?000?0??1000??00?0100??111?001?1???????
??00?000000?12300?00010??????????????????????0?????000??00?000
0?11?0?10?11?0?????0000000000?001001121001 0?0?0000000?000???0?
?0????0??010?00100??0000?001000000?00??0000??00000100000?00?00
00?001100????????00??11????01?1100?10?0000?11??00000??????????
0?0???????0??????????????????????0??????????0??0??????????????
?????????????????????0??
?00010???0???????????????????????0?????????????????????????????

??0?1?00??00???????????????????????0?????0?0???00?0??0??100?00
??0?????0??????????????00?????????0?????????????????0??0?0????
????????????????00100???0?????0????????????1????
Yanornis_martini ?00????????????????1?010?????0?11???0??2??0?????
?00???????????0000011???0????000?1???00??0????1???0????????4?1
???????04??????110?011031110?011?130010???????????????????????
?23?010?2??10??1??0?0121[23]0000300000??0001???00?0???????0???2
1?1?0??????????????????0?????300100012??????????10???????1????
?????????????0?0011?00???0210?[12]??1010000101 0?1?11?112100111?
?0101?0101?10000?0?????021[01]101311[01]1100[01]100?0?1?0??0?00??
????0??00?1110[01]?1?00??1?0??01100????????01??1???????1100?11
??001?11??????0??????????0?0???????0?????0????????????????0???
???????0??0???
??????????????????????????0???0???0??????????????????????0????
???0?0??0?0??????????00111?0?1?00??100???0000??0?01?????????0?
????0?0???00?0??0??100000??00?1??0??????????????00?????????0??
???000?????0????????000?00???????????????00100?????????0??1???
??????0?1??
Apsaravis_ukhaana ???????????????????????0?????????????????????
?????????????????0002?1?0??0????????????????1??11??10200??1???
5?100021??24??????1?010110311000011113003??000?1???1?201000?2?
021?23?03?12?010?????1??12130100?00000??20???1?20?0?????0?00??
21????????????????????????????300?10??????????????????????0?1[0
1]?????1?0????????20010?[01]001??21??1?????????00000?0?100012101
1111?01011010[12]0111110000101021110?[23]120100?11??20?1[01]0010?1
1?110?101211211111?0000?1?0??01101????????011??????0?1????????
0001??
??
???0????
?????01???0?????0??0?0011100010001?1?0?1?0000???01?010????????

???0000??000?????????????0??0??1????????00?00100?????0??????0??
?????0????0000000????00000????000????0?000?10????????0?0?11???
?0?????????
Yixianornis
100?????2?021??1?????0?01????0?1?1?0?????????????????0100??????
000?010???0?00?00120???10?01???1???0000??1?0?40??0???0024???11
?110001103111 0?00101300001000021?101?21200302000 1023?23?0211?0?
?1??????2130100100000??000?0??1000?20??0?000001???????????0??0
??0???0??0?1230010001???????01??1?????????11[01]?????1?0?????10?
20011000?11021??[12]??1010000101 0?1111011210011 1100?0110???01?00
0???0?01021[01]10?31201100[01]100?00100?00?002?101??????11110[01]
?1?00??1?0?001100??????00011?11?01????0100?10??000??1?????????
???0?????????
?????????????????????????0??????????????????????????????????????
?????????????????0??????????????????????0??????????????00?0????
????00111?0???000?100?1??000????01??????????0?????0?0???00????0
??100?00??0??1?????????0???0????00?????0???0????????0?????0???0?
???000?00????00??????0??00100??????????0?11????0??1??????
Sapeornis ?001?????????????????0?010??11000????00??21?0?00?
???0??????0????000010??10?00000020??????00????1???000????0?21
1??0210004??100?????2110111101001110000010112 1??00?212000?201[0
1]00221210????1??01??1001210?10030000000000????2000??0??0100??
0111?000???1???00??0???0000??2300000000????????????????????????
????0?00???000000000100001 00????????0000020000?00000?010001000?
0?01010?0010000?0???010?1[01]10?11100??0[01]00?0000?0000?00?000?
?00?00??000001?00??0?00001000????????001?1?1011?1?110001000000
??1?000000???????????0?0???????010??00?????????01??00?0????????
?00??0???????????0?????000??0?????????000??00???????????????????
?????????????????????00000???0??????0???0????????0?0100???????00
0??000?0??????0?00111?1?1?000?000???0000??0?01?0????????0?????0

?0???00?0??0??100000?000?1?10?0?0?0??0?1???000000?0???0????1??
0???0?0??????0?000?000??000?????000000100?0?0?1???00?1????????
?1?1??
Neuquenornis_volans ???????12????????20????????????????????????
00?????00??00?????
?????????????????1?11011?103110?01111000????????????????????
?????????????010????????????[01]010030??0????0??????0?00????????
???1????????????????????0???????0200?????????????????????????
??????????????????0110???????21?????10110??0100?001?001?0??[12]
??1???01?0???0?????[01]?????????2???20?1??01?????????????????10?
1????????1?0???1011???????0?000???200000110??????????????????0
??0???
?????????????????????????????????????00???0?????????????????
??????000??0100???
???????????????????0111?????0??????????????????????????????????
?0?0???00????????????00??0??1???0???????????????????????????2???
????????????0?????????????????????????0010?0?????????0?????????
????????
Patagopteryx_deferrariisi ?0??????????????????????????????????????
?0010???2?????
??????????????????????????????????0?1????????????211101??????
???????????????2?010001??100121301001?000???????1????????00?0?
????1???????????????????????0?????0???????????????00?1??????00??
111110000?????????010000000????????0????????000????0101?1210[01
]00???[01]?10?0???01000001[01]0001?[12]00???30???????00?200000?0
10120????0?000[12]11001100000?[01]?0??0?100?1?200??01?0???0?001?
???????00???0?1???
???0??????0?0??0?110????
?????????????????0??????????????????????????0020000??00????????
??0?0?00??0001?00000????????00111?00100???????1?0000???????010

0??0?0???0?000???00????0??????00??0??1??????0?10???????????00?
??010???????00000000??0?????000000???00?1???00000010??????1???
0????0???0?1???10?

Cathayornis ?0?????????????????1?010?????0?1??????????0????
???????????????00?????????????00001???????0????????[02]????????
3?????2????4???0??11?02?1031110?011?1300?0?01111?0?00?12000?201
?1022?23?????????1???0??[12]1[01]1?0?3000?0???00????0??1??0??010
0???111?????????0??0???????0????2300[01]0?012??????????????????
?????????0??????0????0111??0010?20??1??10110?101????010?012000
10010110[01]?01020111110?[01]?1?????210120?11?01?0101011?0?000001
???1?0?0?1[01]1?0???????????0??01110????????01????????01???????
???0???110?00000??????????0?0?0100?10100?000???????????????0??
????????0??00?????????????????????????????000??00?0?0?????????
???????????????????????????0??00??00?????????????????0?0100???
????0?????????????????00111?0???0?????????0000???????????????0
?????0?0???00?0??0??????00??00?1?1??0???????????????????????0?
???100??????0???0?????00?00???????????????00100?????????0?????
??????000???

Concornis ??
??00??????
??????????????0??11?02?1031110?0?1???0030????????????120?102??
11?23??3?0???1???1???0?1[012]11??0?3000?0???0????????????????00
1??1???????????????????????????2300???????????????????????????
??????????????????0111???10?20??1?0101100101?0?0010001?0??[01]
001?110??0102?1???10???1[01]1?[12]??????????????[01]????????????0
0?????0?100??1?00??1?[01]1??0??????110?????????????????????1100
110??01???
??
??
?????????????0??????????00111?0?1?0???????????????????????????

?0?????0?0???00???????100?00??0??1????????????????????????????
0?????????????0????????0?????????????????0??0010??????????0???
??????????????

Gobipteryx ?0?????????????????1?01010?0?0?????????????????
????????????11?000?0??????????1?1?????????????????????????????
????????????????????2???
???????????????????????11???????????????????????????????????1?
???0??????????????????0????????1100100?00110?10?????00?????0??
?0??0???1?????????????10??????????11??10000?0?100012000[12]001?
1?0110???0????????????1?121??[012]0?????1?0??1????????000?0???1
0?001111000?1?11?0???
????00000??????????0?1?01????0???0?0??????????????????????????
??0???????????????
?0??????????????????0?01000?000??00???000?????????????????????
??
????????0??0????
???????????????????????????????????????0???0??????????????????
0????

Vorona ??
??
??
?????????12?01001110100112110???00????????????????????0?????2
??
??
???10101
11100001?000000?????????????????????????????????????00????????
??
??
??
???1???????00000000?00

??0?10001?????????????
???????????????????????110?00000010??????????0????0?????1???1??
Songlingornis ?????????????????????0?0???????????????????????
???????????????00?????????????000?0????0???????????????????????
????????????????11??[01]???3???????????????????????????????????
??????????????????????????????0????0?????0?????00?????????????
????????????????0???????0?????????0?01????????????????????????
???????????0???????????????21??[12]?000100?010???1?11011???????
??
???????????????????0??0?????????????1??1??????????????????????
???????0??
??
???????????????????0??00??00?????????????????0?0?0????????????
????????????????1?1?????0?????????????????????????????????????
???????????????????????1??????????????????????????????????00??
??????????????????????????????????0?1?????????????????????????0
????
Pengornis_houi ?0010??????????????10010??0?00??????0?12??0????
??0?????0??????000?001????????0?0?00??00???01??????20???0????2?
?????????4????1?????2?103111?0??????03??001?1????????????????
??2??2??????????1??????211???0?000??0??000???0??0??00???0???11
?1?000??????????00????00001230000?000??????????????????????????
????0?????0???0011??00100????????01[01]00?00??????0??1??012111[0
1]0?011010001[01][01]1?????1??1???10[12][12]0??1??1?11??????0????0
0?0????0??0[01]?1?00??1?11??0?0??0?100????????01????00?01?1001
011???1??110?00000????0??0??0?0?0100??0?????00???????????????0
??????????0??000??????2?????????????????????0?0??0????????????
?????????????????????????????0?0?0???0?0??00???0???????0?0100?
?0?0??0??1?0?????????????00111?0?1000????0???0000??????????????
?0?????0?0???00?0??0??????00??00?1??0?0???0????1??????????????

0????1?00?????????0??????0?0?????00?1???????00100??????????1?1
????????000???
Hesperornis ?0?????????????????00010100?00??????00021?001??
??00???00????1?000?001???1?0001?021???00??10101110?2100??11?50
100?210?24??11??10??00103111??????????3??0202100?1??01000???02
1023?03?12???0001??11?121311001 0??0?00??01?1?0000?000???00??2?
?1?1111????0???????10000??10?1?10?1121200110?1010??1110011100??
00?0???10101210110000 12?0?000100100?0???10?0???00011??0???????
??0???0????0??????????????????????????20?11000011121102111100 21
11112212200 2?0?????00??????0001?0???0?000?0100?11???1??01??000
00??????????0?1?0100??0?????00????????0???0??0??????????0??00?
??????????????00??0?????????0?0??0????0???????????????????????
?????????????0?000???0????????????????????000???????0?0??000??
?????0???01??????????????201??000????01?????????0????00?0???0??
00?0??????00?????1??0?0?0?0???0???????????????0?????000???????
??0?????0000?????00?????????001?0??????0???1??????????000???
Baptornis ???
??2?????????5?
????????24??
??????????????????????212???????????????????????????????????2?
??????????????????????????????[12]????????????????????11001??0
0??1??????????1210110000 12????012?0?????01?10?0???000????0????
?????0???0??????????01?[01]1[01]???????????????20?11000011121102
11110021111 1?21020?2??
??
??
??0???????0?0
??000???????0???01???????0?????201??000????01?????????0????000
0???0?????????????00?????1??????0?0???0???????????????0???????0
?????????0?????0000?????00?????????0210???????0???????????????

?????

Ichthyornis ?????00????????1220????????????????????00011??
??0????????????000??1?????1000??0210??10???1?01111?2100?1???5?0
00?21??34???01?100?001031110?011113003??0102?1??10212000????21
023??3?12??10011??11?1213110???00?0??20?????00????0??00????21?
??????????0?????????????012300[12]0?11???????????10[01]1?1110111
01001020???101?1200111?101[12]?211022010100001010?11110112110111
100101101110100001 01[01]1011211123120110011?12001[01]0010?112110
211110021111[12]2?100002?0??011?100?20???0110??1??00?00100?11??0
1??01?????????????????????????????0?00?00??????????????????????
??????????????????????????????1?0?????00????????????????????
??????0????0100?????0?00?0000?00?????????000?0???????00??000??
0?01?0000??????0??00111?00100?0?10201??000????01?0????????0????
0000?0?0???0?0??1???00??0??1??????000??00100?0???001??01???2?0?
00000?00000000????0000?????00?111?0?00001????10?1?0?0?11?0??0?
???0??0?

Iaceornis_marshii ??
??
????????????????1101?010311??????113?03???????????????????????
?????????2??1??1???????21?????????????20???1??0??????????????
??
?????????????????????????21?02[23]11???1101010?1111011210 1??????
??????????????????????????1113140111011102 0010??1?1?121102121100
21??0100?11???1???????
??
??
??
??????????00111????????0?10????????????????0??0??0???????0???????
????????1???????????1????????????00????0???00???01??????????????
0??????1??????????????1???0?00??????????????????11?0??0?????????

Limenavis_patagonica ??
???
???????????????????????????????????011?13??????????????????????
???20??????????????????
???
???
????????????10000[01]010101011?11[12]313011?011?????????????????
???
???
???
???
???????????????????????????0?0?????????????????????????????????
???
??????????0000???
????????

Lithornis
100????????????????01010100?00?111??2?02?00?0???????????????10
000?00????02?101?1?????????1????????2????????[67]?1????????????
[12]??110000103111110011?13003??000210101?20200102??2102?????12?
01000???11?12130100?????0??200?01?2010???0?0?0???21???12??????
?????00???00000??300?11212100?00011111010110?11[01]1101?1111?01?
10121012?0111[12]12111221101001?010110111101121011111001100011[12]11
100001010101011111131301101[01]11102[01]0120110?11211020110002111112
2210010[01]?000011?1????????011?111??00???????????01???1???????
???
??????????????????????????????????????0????????????????0???????
???0???????0?0?????????
????????????????????????1??000?????????????????????????????????
?0???0?????????
??????0000??

Hongshanornis_longicrest

101?????????????????11010?????0?11???0????00??????????????????

000?001??00????1?1?10???0??0??01??1?0????????[234]??????????4??

?1??11?01110311100011113002 0?0112???01?20????????2??23?23??2??1

00?1??????21[23]0100000000?000?01?1????????0?0????1?1?0???????

?????0????0000?1?300???012???????????????????????????00??????1

?????0?????110????????01?0??11???0?10???2100011???110???2????

0?????????????1??2000??000100?0??0?0??000????????1???1100??[01]?

00??2?0???1????????????????????????0100?11??001?11??????0?????

?????0?????????0??????0???????????????0??????????0??0?????????

?????????????????????????0???????????????????????????????????

??????0?0?0???0?????????0????????????0???????0????????????????

??01?1?0?000?0?10??????????????????????0?????0?0???00????0??1

00?00??0?????0??????????1???00?????????0?????????????????0??0?

0?????????0?????????00100?????????0????????????1????

Liaoningornis_longidigitu ???????????????????????????????????????

???

?????????????????????????????3??1??0?????????????????????????

?????????????????100?1????0?21211003000?????0????????????????

?????????????????????0???????????????????????????????????????

?????????????????????????????011?01??????????0???0???????????

?????????????????0?????01???2??????????????????????????01?1?0

??0011111102010 00?00??

???

???

???

?????????????????????????0?????????????????????????????0?????0

?0???00???????????00??0???????????????????????????????0???????

???????????????????????????????????0010??????????0???????????

???1??

Crypturellus_undulatus

10????012?020002?2001010100?00?111?12002100001011100010???1?10

000?000??102?001?1?????????1110111122100?01107?110??12?24??121?

11010010311011011113003??001210001?202201120021023?23?12?010001

00110121301?0???01000200001?20000200?0?001021???120??01??????0

????00000103002112121[01]01000111101011001101111111100101012101

011211212111112?101000210110101111210011110110001201100001110 10

1011111031401011111020?120110?112110202110021111222100101110??011

?10102000001111110000??1100?10??01??11?????????????????????????

???

?0??0??????

???

????2??????????????????????0???????????????????????????????????

??0????

?????????0???????????????????????????????

Gallus_gallus

100???102?021002?2000010100?00?111?120021?0001?1000001????1?10

0100000000021101?1?????????101[01]1111221?0?01007?120??12?24??1[

12]1?110120103110200111130 03??00121111?2?2001120021023?23?12?01

00011011??2130100100010002 00100?20100200?0?0???21???121??01???

0??00???00000103001102121211111221101111111111110110001101011121 0

101120111211112110100021011111111112100111101100012011000010111 0

1011111131401001211021?121111?112110202110021111322200111?00?011

0001020?000111111?000??0010110??01??11?????????????????????????

???

?0??0??????

???

????2??????????????????????0???????????????????????????????????

??0????

?????????0???????????????????????????????

Crax_pauxi

1001011?2?021000??001010100?00?111?120021000011100000100??1?10

0000000000021101?1?????????1000111122100?111?7?120??12?24??121?

1100201031112101111300 3??0002100?1?2?20011200210 23?03?12?010001

1011?1213010010001000200 1?1?20100200?0?0???21???121??01??????0

0???000001030021021212111112211011111111111011000110101112101011

20111211112110100021011111111121001111011000120110000101110101 1

11113140101121102 1?111111?112110202110021111322200110100001100 0?

020?000111110000?11100011??01??11??????????????????????????????

??0??

??0??????????

???

2??????????????????????0???????????????????????????????????????

??0????????

?????0??????????????????????????????

Anas_platyrhynchus

100???102?021002?2101001100?00?111?10002??00?1?100010?0???1?10

200000100?021101?1?????????101?1111221?0?011?7?11???10?2410121?

11011110311111011113003??0012000?1?2?2001120021023?13?12?010001

1011?121311?0???01000210 1?1?20100?00?0?0?1021???121??????????0

0???000001130021021211111112211021112111111101100011110111210 1010

2011121111312010010 1010?1[01]111112100011100110001201100001011101

1111112314010101110 21?111110?1111101021100211 11[23]2210010210?001

1?10?020?000111111?000??0100?11??01??11????????????????????????

???

??0??0?????

???

?????2??????????????????????0??????????????????????????????????

???0???

??????????0??????????????????????????????

Chauna_torquata

000??1012?0210002?101010100?00?111?100021?0001010001010 0??1?10
0000000??0021101?1?????????1011101112100?111?7?11???1012412111?
100000103111110111131 03??0012101?1?2?200102?021023?23?12?010001
1011??213010010000000200101?20100000?0?0?1021???121??01??????0
0???00000123001102121[12]1111022110111121111111011000111101112101
210201112111140101001010111110111211021110011000120110000101110
1011111231401011111021?111111?1121102021200211112222001001000011
0001020001011111110000?11101?11??01??11?????????????????????????
???
?0??0??????
???
????2??????????????????????0???????????????????????????????????
???
?????????0???????????????????????????????

Pedopenna ??
???
???
???????????????????????0?0?0??0010?????????????????????????????
???
???
???
????00?000?????0??0????00????????0???11????????????????0???????
???
???
???
??00????0??
?00???????????00??0????????????????????????????????0???????????
????????????????????????????????0?10???????????????????????????
??

Epidendrosaurus ???0??1
????????????????10??????????01?????0???????0??????1??????????
???????????????????????001??1??0??????001????????????????????
?????????????????0????????0000030?0?0???0?????1??????????????
1???????????????????????????3?0??????????????????????????????
????????????0???????0??????????????????0??0?0??????1???0?????
????0???0???0????????????????0??????000??????????????????????
???100?00000??0?0??0?010????????00??????1???????????0??00?????
???
???????????????????????????????0????0????????????????????????
???
???????????0011??????0???????????????????????????0?????0?0???
00??????????0?00??0??????????????????????????????0???????????
?0????????0????????????????????0010??????????0????????????????
Epidexipteryx ?01????????????????????1??????00?????0??2??0011?
????????????????100?0?0????00001020???10000???????????????0?[0
1]????1?1?123??10??0????0101?010?0??????00?1???????????022????
?011?03?0??????????0????011?0??0?10?000???00??1?1?0???00?0?00?
??0?0?????????1??2?????0???????2?0?????0??????????0???????????
????????????000?0????00??0?0??????????????0??0?0??????1???0???
??????????00?[01]0???
???????10?0?0?0????10?00???0?????????1??11??1????????????0?00?
100?????0??????????0?????????0?????0?????????????????????????
????????????????????????????????????0????0???????????????????
????????????????????00200??02?????????0???????0?1?????????????
???????????????0011??1???0?????00??????????01?????????0?????0?
0?????????0????0?0????0????????0??????????????????0???????00?
?????0???0????0?????????0??????????001?00????????????????????
0????
Incisivosaurus_gauthieri ?00?00101?001??1??10001110100100 1?010?1200

00110001010110101111210100001001?1?000210??00?0??????????????
???
??00??00??01?0??0?0????
?????100000001000102?00010?100?????0000000000100001??0000???0
000101?1100??0000??
???
????????????????????????????01020?00???0??000?0?0???????????1
1?1100000????1000000?0?0000?000?0?000???1000??1??00?0????000?1
10??010??01?0000?00000??00?00????0100??0111000???????????????
?0010000000010000????00200??02?1?000000000?????00110?????????
???
??????100?????????????0??0?001???????????????????????0201100?
??????????????????????????????????????0?0?0??????0??????????0
00?0?
Citipati_osmolskae ?001001001001??2210001011111?01011?000102100011 0
00010001000110121120?0100010111?1?????????1011101100101211001??
201??002200111?0111200110100100?11000001000211001??02012020??10
111111?2101000000000?0000000000000000000110200000000?0?001000?00
0002011???0???[01]00002000122000100000000 1102?1??0000??00000100
01010??0000020011000[01]0?00?0010000101020??????0????111000?0?0
010000?000000000000?00100?0??0?0???0000000????00000?2000000000
00000000002?000?00000000020?000110??00[01]10?0110110000000 11?
1100001????1000001?0?0001?11000?001???1002??0???0?0????000?000
??000??01?0000?000000000100?????100??0111000????????????????1
0100000000 10000????10201???2?1?0000000???0???????????????????
???????????????????10???00?00??????????????????????????0?0?0?
??00100???000?00??0???0?00?????????0?0?0?00?0???0?00211??????
????0??00?000?0???????0??????0?0000100?0100???10?????????1?00
?0?
Oviraptor_philoceratops ?00?0??????01?1????0??1??111?1011???0???1?0

?11???01?0???0?11?121120?01?00?01?1?1?????????????????????????
??????0????????????????12??1?????00???10??00?00??1??0?????????
???????????????????0????0????0??0?????00?00000???2?00?0???????
?????10000?0?0???????0??0???0????00???0?00????????10?0?00??0??
00?00?1?1????00???00?100????0????????01010?0?00?0?0????1?100??
????1100???0????????00?????????000?0??00000??????????????????
???????????????????0??000?????20????1?0???02?0??1101100???0011
?1?????1????10????1???0?01????????01???????????????0?????0???0
0??0?0??0??00?????00???????????????????0?????????????????????
???????????????????1020????2?1?0000000???????????????????????
????????????????1???1???0?????????????????????????????????????
?????100????000???????????0????????????????0??0???????211?????
????00??????0??0??????????????????????00??0??????0???????????0
0???
Microvenator_celer ???
????????????????21?20?0????????????????????011?0?11000121100?
??0?1?1002????????????00?000?11000?????0010002???0000?????????
????1110??0101100?0000010???????0??0???00??11?20?0??00???0?1??
0?????????????????????0???0?2200000???????????????????????????
?????????????0?0001????????????????????20000?000?01?????00?0??
010?00?0000000000000010000?????00????????0???0??000002000000000
000?????????????10??0???????????????1????????0????????????00?????
???
???
???0???00??0001?01
001?????????????010000?0?????100???????01?01??0000????00??????
???????0?????0?????????1????000?10?000???0010?0001????1??????00
??00?10??00000000??000?0??00000??????????1?01??11100??001??????
Caudipteryx_zoui
00010??????????????0?111??10?0001?10???21000???0?0????????????

21120?0????????0?1??0?????00????0??00????1???0???01?????30????
?0???0??0???01?0???100000?000?1??????0201202???1????101?11????
?0????01000??1?00?0?000000?1102000??00?0?001000100000?00????0?
??00?00000?1[01]2000000000???????????0??????0000????1000???0000
?00?0000?0?0?????????????20??0?0?000?11??00???????0?0???00?00?
???????????1??0000???0000000?000000000??0?0?00????0000000??0??
2000000??0????????01101100??01?110???0??000?10???0000?????0???
?0?0?0001?00?????0????????????????0????00???00??0?0??0??0?????
????000?0????????1????0???????????????????????????????????????
??00201???2??????????0?????????0??0???????????????????????????1
???????00??000???0000???????????????0?00??0?0???001?0?0???00?0
???0????10?????0???000??0?00?0???0?0???11??0????00????0?000?00
00???0?0?????0?0?00100???0??????????????????1?10???
Ingenia_yanshani ?00?0??????????????????1?1?????1?????????????????
???????????????21120?01000?0111?1???????????????????????????1?
??01????2?00???1011120011??00000??100000100021??????0201202001
1011?11101101?0000?00011?000000000??0000??11?2000?000?0?001000?
?????????????????0002?0??2200?1000000??1?????0?0??0??0000?00?
?1?10???000??0??1??0[01]0??02??????0101??0??0???0?0?0101000????
?1100???00?0?????????????????00????0?00?00?000?0?000??0?0?000?
0?0?0??00?0????00?000000????????0110??00?1?0?1101000??000?1???
??
??
??
???????????0011000?001?0?01??101000??0101??11??0??????00000???
00?????1?000000010??1?1?0??????10???0???0??0?000????11???0???0
1000000010000000??000100?000000010???????????0??1?0??00?1???1??
Rinchenia_mongoliensis ?00?0????0??????????0111?1???1?11?00010??0
0???0000?????00??1?12112??01000??111?1??????????????????1???????
???????0?????220??????????2??1???00?0???1??0001000?1?????1?????

??????????1?????????0????0??0?0???????0??00??001??2?00????????
??????0??????00????????00??210??????2??0120????????10?0?10??0?
??0?????????????0??
??00???0?00?????????
???????????????????????????????????????002?0?????????????00?
1?1?00001????1??0001?0?0001?11??0??01???1????????????0????000?0
00??000??01?00???0?000000?0????????100??0????0???????????????
????????????????0??
?????????????????????????????????1?1001??????????????????????
???????0????????????????????0????????????????0????????????11????
????????????????0??????????????????????0????????????0????????????
?0???
Conchoraptor_gracilis ?0010???????????1???00111?1???1?11?000??21000
110?00????1?0????121120?010?0?0111?1??????????0101011001 0??1?0
12?110??10????01?1?????20011000010???????00100021000101020020 2
0??101101[01]1?11010000??00010000010?000??000?001102?0?0000?0?0
010001000002001????????0?0?0200012??????000000000????????10???0
100100????????000??0?010???????????????????????????????????????
??00?00??00000200000
0000000?0000?002?01??????????0?020?000??0??002?00????????????001
1?1?00001????1??00?1?0?0?01?11??0??01????0?2?????????0????0?0??
00??0?0??01?00???0?00?000?0?0???????100??0???00???????????????
???10???0???????0??
????????????????????????????0?0????????????????????????????????0?
0????010?????010?????0?????0????????10????????00?0????????21?????
??????????????0?000000??????????????????0?1?00?????????00????????????
?0?0?
Chirostenotes_pergracilis ?????1??01?01101??0???1?110?0?????????????
?????????01010??????21120?00000201???1????????????????????1101?12
????1?12???0??????????????????101?1????????????00?100021?001010221

20201110?111[01]??01??100???00?100000200000??000?0?1??20?0?000?
0?00100????????????????????000?????[12]????10????????001????00?
???????????01?10???0????0011??00????????????????20000?00000???
???00000?00??00000?
00?0????????000000?0???0000?????001020?00???0????????1???????
0?001???????????????????1?0?00???0?????????????????????????????
???
????????1000001000???????00200???2?1?000??0000??????????0?????
??0?0??00???1000?0??????????????0??0??101000?????1?0100?0??0?0
0??00000?001?0??1?1?000?01?????1?0??????10?????000??0??????02?
1???0110?1???????000000000??00????00??00010?0??0?????1?0??0??
?????0?1??
Avimimus_portentosus ?00?0???10011?00??0??1?1???????1???1?????00
?11??00100110??????2?1???00?0??0111?????????????01101011010?1?0
0??100?????????????????????????0100??????????00?211?01??0201202
010?011?0?1000100?0???00011110020???00??0?0?01?02?000000?0?001
000????????0?????????00?01??0?02001????????????????????0??0???
00??0??????????02001000??????????????????????????????0?????0?0
?0010000?000000000????????????2???????????[012]0?000?0000020?01
0000000000000??00?01??0??????0???????????????????????????????
1?0??
???????????????????????????????????100??0?1100???????????????
?????000001?0?00?????????????????????????????????????0???????00
01000001???????????????00001???????101001??0101?0100?0?000??001
000??00???????0??0?1001??101????0000100000?????1001101102?????
101010000000???00?000???0010000000000010?0????1??01????0??00?1
???1??
Falcarius ??01?1110000111010?????1????00??????????00?????
?0000000??????100?000??0???????001010001?1??00011101012101000 1
2000100??0000??????00000100010001 0?0000100?0110010102203010101

0010001000000000000011000000000000??000?10?00?0?000?0?001000?
???????0?001???00?0?0?0??11?0??000???????000??00???0??00?00100
?0?00??0000??0010000[01]0??????????010002000?00000001110?1?0?0
010000?0010000000000010000???0100???0000000?00000000020000000000
000?00000000?0000??00000?0?200000??0???00000?1100?00000000????
???????????????000?00?00100????0??????????????????????????????
??0?????0??????????????01?00????00?00????????????????????????0
000001010010????0??0000001???0???????????????0?0???????0001000
001000??1000??10100000001000000000000?0101?010000000001?0000001
000?0??0010100001 0?1?01?11?100010010000000001110100 2?0?10111100
0000101000100000??000001000?000000?0?10?10000?011011002?00?10?
Beipiaosaurus
0??1??
?10?10?????????????1001001?100????1???????????????000??0220????
?????0000??0????0?1000000?0000??001???20220??0??0????0??00?0??
00??????0000????0???0??00111???0?0??0????0010?0??????????0????
??????0???0??????0???????????????????????????????????00????0
01000?????????????00?00???????????????????????????????10000??
?????????1??0100???000000???0??0??0?????0???????000000000???0
?0?00000?????????????10?????1?1100??0??000?1?0?????0??????????
?????????0??
??
???0??0?00????????????????????????0???????0????????????????????
1???0?00??00???00?1?????????10?????0?0??????????00????????1?1?0??
??????1??1?????10???000?0?00????????????????????0??0?01000?0101
0???0?11??10??0?1?0???1????????????????????1????
Segnosaurus_galbinensis ???
??????????????????????21??10?0000?000???0100?001?????????1??????
????1?0?0????????????????????0?10?1?00000????0???200111001020202
21??0110201101?11001?00?00?1100000021?0??00?01?1??0?0???00?0?

010?00??????????0?????????00???????0??????????????????????????
???????0?0????0??????????????????????????????0??????0????1????
1???0010000?00100000?????????????????????????00?00000??10?????
?00?????000000000??21?0???00????????????????????2??????????0??
??
??
??
????????10????????????0?00???????21020010?0101??02???????????0
?00??????????????0??01??0??1??1????????2???????0??110????????1
???????10100??1?1112221011111112?111201??????????0???1????????
?????
Erlikosaurus_andrewsi ?0012???2?0?1?1??1010011100??0001?100000100
0010000000000??11112100100000020001?001001001????????????????
????????????????????????????????0?????????????????????????????
????????????????????????????0000?021?0??00??11??00?0?00???????
?????0?00000010010??00000?000????0?000002000001???10?0100???00
00?0?10000??0000??1?
0?0010000?0010000000??
???????00000000????????0000?0????000?????000?0?????????0????01
?0001100????1?10000?0?00?0?100000000???0??0?00??00?0????000001
000000??00?0200?00000101000?00001?000??00?0?00????????????????
00000001101????0???01001000200000000000000??????0?????????????
????????????????????0?00??????????????????????????????????????
?????0000???????00??0???0?011????????2?????????????11210?11??
?????1?0?1????????????????????????1?01001000????01??????????01
0?0?
Alxasaurus_elesitaiensis ??
????????????????????210?100????????????1001001??????????0?0101
0000?1?0101002?0?1???????????0?000??0?10?0000120?11??0??1?20?2
1???11?????????1????0?????????00000?10000??001?1??00?0??00?0?0

???00??????????0??????????0?????0????0????????????????????????
??????????0??00????0??00??[01]0????????????????0????????0??11??
01??????0?0???010000???????????10?0100???000000???0??0????????
?????????????????????1000000?0????????0?????????????????????00
0???
??
?????????????????????????0??0020?????????????????????0????????
????11101?????1??0?11??0?00??010?0?????01?????????2??????????
?????200??0????1?1??0??0????1??1?100???????0000000??1??0???0?0
0???11?0????01100??11?1?????1111?????1?00???10?1???0??????????
?1????

Neimongosaurus ???
?????????????????21????0?????????????0??001?1??000?110101?1?0?
10?2?1?1?02200????????0001010000??????????????111001?2????????
?????????????0??00????0??000000021?00????0??1??00?0??00?0?????
0???????????0???????????????1[12]200?0?????????????????????????
????????????0?????00?0000?0??????????00000200?0?00000?0?11001?
0?0?10?0???010000?0?00?????????????????????0???????????20?00??
?????0?00000000?0210??????0????????0??????????2?1100?000??????
??
??
??????????????????????0????2??????????????????????0???????0??1
??11???????0??01?10?0?00???????21?2????????????2?1???1?00??000
00?1???0??00????00????00??????100010?2????????00?????1???0?0111
?111001011????1?1121?????11111????10????1??11??0?0??0?100???1?
1??

Erliansaurus ???
??10??1?????
?????1???????????????0?0???10?0?0???00000????1?0?1????????????
??2??1???10?00?0?0001?0??00???????????00?????????????????????0

?????????????????????0??????200???????????????????????????????
??????????????0????0???????????????????????????????????01?0?0?
10?0???010000???00010???????100???000000???????????20?00??????
?0????????????????0??????????????????????????????????000??????
??
??
??0???????????????
????????????????0?0?1?0?01????????????????02?0???1?000????????
??????00100???????0?????????00???100?????00?11????????????????
011?11111??11?1???0?1?1111011?0?????????1??????????0?1??????
Suzhousaurus ??
??0101??0100
0??0??1????0??????????001010000???????????200111001?2020?2?100
10020110101100?00??????????????????0???????1????????00???010??
??????????????????????0?????0?0???????????????????????????????
??????????????20010000[01]???????????????20000?00000?01010?1?0
?0010?0???0100000??00?????????????????????00?000?00?1020000???
??????????????????0??????????????????????????2????????????????
??
??
??
10111100100000?11010?00??????021020010?0101?00201010??????????
??????????0???0??01??00?1????1000?0?2???????00????????????????
11?100011????111221211110??????????????????1?0??01102?0?2?????
??
Nothronychus ?????1112??11?1?0?0???????????????????????????
????00000????????????????????????????1????????????10101?10?01?
1?00?100220?001?????00000?100?00????00000200??10?1?2020221001
00201101?1100100?100011000000021?00???00??????0?0?000?0?010?00
?????????????????????0????1[12]200???????????1?????????????????

????????????????0010000[01]0??????????00000200?0?00000?0211001?
0?0?10?0???010000???00010???????000???00???00?000000?1020??000
000000?00000000?0210??0?000?0????000????????0???1100?000?000??
???
???
??000?01100??????????????????????????????????????0???????0?0?
?1010110???0000?110?00001?0?0?021?20010?1101??02???1?10000?0?0
00?10?????0?1?0000010?0??1?11?1?0010020000?00001110?1?2???????1
?1110?0001???1111222101111 1?12101120??0????1?1?0???10?????1???
10?
Enigmosaurus ???
???
??111?01?2020221100
??0201101?????????????????????????????????1????????00???010??
???
???
???????????????????????????????????????00?000000?10??????????
??2??????????????????
???
???
???
???1??1????????????????????2?020?10?1101?????????????????????
????????????0??01?????1??????????????????????????????????????
???????????1?122121100??
Nanshiungosaurus_brevispinus ???????????????????????????????????
??1???????1
01?121???0???
???
???????????????????????????????0?????1???????????????????????
???????????????????????200??0????????????????????????????????

???0???????????
???2????????
???
???
??0????
??????1???1??0??????????????????????21?2??10???01??????????
????????????????????????01??0??1???????????????????????????
???????1?????????????11222??11????????????????????????????
???????????
Therizinosaurus ???
???
????????????????????0010?0000????0?00200???????????????????
???????????????0??0?1100000021?0????00????????????????????
????????????????????????????00?????????????????????????????
??????????????????0?????????????????2000??00000?01?1001?0?0?
10?0???010000?????010???????100??00000??????????????????????
??????????????0??0100???????????????????????????????00??????
???
???
???
??????????00?1010?00100101?????????????????????????????????
??????001?0????????0??????????10??0000???????????????????????
111011111????????????????2?111?0??????????1???01????0?????????
Haplocheirus ?0010???00010?0?12011011101112001?000020100?0?1
010??0001?0010000010110110?1000001101010100??10??0?0111201??0?
00001?0???0?00??????100210001210?0?00010???001?00?10100201001
0?011000?00000000?0000?0000000??00?111000110001 0001?0???0010?0
1000000000??0000001?00000?10200000000???00??00???0???000000
??0??0??0000?000?000????0?????????????20000?000000010101??????
100???000?00???????????0???00000??0000000?00?00010020000?????

??000?0??0???0100?000???0?0??????????00?0??????????0?000001?0
0001??0?0000000?0?01000000?0??11???0??0?01??01?0????000010000
00??00??100?00000000000?000???00??0??0?0??????001????????0?0?
?0?0000????0???00000??00?00000??00?????????000???????0??1????
???????00?1010100??0100?00?0????????0?01??0???00?0?????0?????0
0000?00?101000??00?0??010?1100100100010000110??00??0?101???00
000???0000?????????000????????00101???0?1?1?010?????0??0000??A
lvarezsaurus_calvoi ??
???01000???0???
0?20?0?2012?????????????000?00????????0??1?0000101?00?2????????
??????????01?000??0??0??1100000??00????????1???0?0??00?0?????
0??????????????????????0????10????????????????????????????????
???????????????00100???0???????????????2?000?????0?0?0???????
???0??000?00???2????0000
0000?000???00?02???0??????????????0????????0??????????00???????
??
??
??0???????0001?0
0????????00?10?0?0????????????000?000????????????????0??00????
?00?????????????0??0??0????????1000???????10?????101???????1???
?00?????????00000???????1???0?000?10??????1???0?0??0??0???????
??
Patagonykus_puertai ??
???1?01?112???
?012010?2?2??????????????00210???210?????1?0???1????1010?????2
0????21202??1100000000001210??0????001??0???????0?????????0??
??0?????????????????????0?????01??????????????????????????????
???????????????0?00100?????????????????????20000?000?00??????0?0
?0000??0?0010000???0???????????1???????????0???0????10020000000
0000??00?0???????10??0?????????????????????????????????????0????

??
??
??
00001?01100?0?????000?1100??????11???0?0?01?01?1?000?10??1????
0?00??????0????00?000?0??1???0??1001?101??1?0?111111??????????
?0?00000??0?????00??0000?00?0?00??1?????????10??1???0???0????1
??
Achillesaurus ???
???0
20???2??????????????????????????????????????10??01?2??????????
?????????1??????0??1?01000??0???00????????1???????????0?????0?
?????????????????????0??
??
???????????????????????????????????????0???????????2????000000
00?0??0??0??
??
??
??
????????????????????????????00?0??????????0????????00?0????0??
????????????????????????????11????????????????11?????????????0
????????????000???????1???0?00?????????????0?????0????????????
Mononykus_olecranus ??????00???????112?????????????????????????
??????????100???????????????????????2????00????????1?1?1110200
1???2?10?2??????1???1000?000200030210?02201100??1????1?201000?
2?????3??0??0210100111011212000030000001??00???1??0?0??00?0?0?
??00??????????????????????????00011???????????????????????????
????????????????0?0010?0?????210???00?????2?010?0?0?0?010?011?
0?0001000?001000000000??0000???30000???0???0??00???????2000000
000000??0000??0?00?0??0??000???????01?0?????0?0????????11102??
??

??
???0???????1001
001000????????1010100000100?????011???0???01?010000010101?1000
00100????0??????00??1??0???021?1110010111111111101011???????010
0000000000?000?0000??000010000?000?10????????100?10100?0?01???
1??
Shuvuuia_deserti ?00101002?0101112011000101l?01111?100201000010l
0010010???1110?00101001002100??0211??000??0111111011102100?0?20
1012012110100?1000?00020003021??02201100000101001?201000?200220
3??03?02101001110112120000300000011000000110000000010000?00??0010
00000001?010??000000000?000100000000?101000?10000110?0111000??
0[12]0????0100?0010000[01]0?0210?0?00?????20010?000?01010101l?0?
0001000?00100000?000?????????30000??000??00?000?00?10200000000
00000?0000000?00100000?0000020?0001?01?1010001???????1110200?0
00?000??0?1??0000?0?00000?00000100???0??0?00??01?0????0?????0?
?000??00?0200?000000??0?0?0??00?000?001?0?0??????????????????
000000000????????00000?000?0??000000??00??????0?0???????000100
10001011?000101010000100??0?01110000???01??1??????????0???0??
??00001????1??00??1??00110210?110010?111111??0?01002?0??00??0?
0000000?000000000??000?????0000001010l?0??0?00110????00?1101?0?
Parvicursor ??
??10???????
??1??2??????????????????????????????????????1????2?1000???02
203??03??2??100011?11211000030??00?????????1?????????0?00??0?
?????????????????????0?????0??????????????????????????????????
????????????0?00?00???
?????????????????????????????????????0????????1?2000000000
00??000?000?00?0????????????????0??????????????????11?????????
??
??

???10
00?????00??????????????????????????0???01??10??00?0?01??000??10
0???????????00??1????????1?111???????????11100010????????????0
????????????0?????000??00000000010???????????0???1?0????1???1??
Albertonykus ??
???
??????????????????????????????211?????1?0??????????????????????
???????????????????????????030?????????0???????????????????????
???
???
???
??0??02??????
???
???
???
????????????????????1???????????????????????????????0???00????0
???????????????????????????????????11???11??1??????????????????
?????????????????????????0????0????????????1???????????????????
Albinykus ??
???
??0????????????????
????????????????1101??121100300000??????????????????0000?????0?
???
???
???0??????0????????000000
000?0000000?00??0????00????????0????????????????????11?????????
???
???
???
??0?010000??0

0???????????00??1???
??????????????????????????00000?10??????????0??????????1??????
Bonapartenykus ??
??112??0??
??????????????????????000210???????????????????????01?????????
???212?2??1????????????????????????0???????????0?0???????0????
????????????????????????????0?????????????????????????????????
???????????????00100???????????????????20000?000?000??10??????
???100??????????
????????????????0???
??
??
??0?1000
??1?????0?010?010??????????????????0?0?01?????????????????????
00?????00???????????0??????1???011????????????????????????????
?0??????????????????0?????0????????????????????10????0?????????
Ceratonykus ?0010???2??10?????????????????11???10?201?0?0?
0100??0?????????????01?0??????????????????????????1?1?????????
?????????????????10????0?2???0????????????????????????????????
?????????????1????????212?0003???00????????????????????0?????0
???????????????????0???????????????????????00???????1????111??
??????????????????????????21?????0??????0000?0???01???????????
????????0??00000
000??000?000?00?0????????????????1????10?0???????????????0??0?
????????????????????????????????0?????????????1?0????0?????0??
000??0??02???0?0000??0?0?0??00?000??0????0???????????????????0
??0?00??????????0?0????????????????????????????0???????0001???
??0?0??1000???
????0??????????????????1?????1?00?0?????????????1?????????????
??????????????????????????00?00001???????????01????????????????

Linhenykus ??
??111?101?1??1?0??
?2010020?2???????10?0???1??????????0?21110??????????2?????????
?????????0?1010001????2?2?0?030?0000???0????1??????????0?????0
???????????????????????????01?????????????????????????????????
?????????????0?0012?0?????210????0?????20??0???0?0?0??????????
?????????10000???0??????????31??????00??0?????????1?2000000000
000??000?000?001?????0?0?????????1???????0?????????????02?????
??
??
???0???????0001?01
000?????0??????1??????????????????????????0?0??00?0?0???000???
00?0??????1??0????????????1011????????1111?1???0??????????????
0????????00???????????01??00?00001???????????0?1?110?0?????????
Xixianykus ???
???102?00?21
201??0????????????????????????????????????0?01?1??1?20000?2002
2?3??03?02?010011101121211??3???000???????11???????00?0?00??0?
?????????????????????0??
????????????0?0010000???
???????????????????????????????????????00?000000?1?20000000000
00??000?000?0?????????????????????????????0???????????????????
??
??
??10
00?0??????????????????????000110000??01?010??00?0?00010?0????
??????????????????0??1???1011????????????111??11?????????????0
??????????0?0000??000010000000001??????????0????100????1???1??
Nqwebasaurus ?0????????????????02????2010????????0100?00??
1?????0?00????????????????????????02????1??????00?11001???1???

???????????????????????0102?000??0?00?10110??????????????????
???????00?????0?00001000100000000?000?1??00??????0?0?0?????0???
?01?00???00?????????????????10200????????????????????????????
00???????????????????????0?????????????20????0?0???0101?0????
??10?0????????????????????????00000??00000??????????00?????????
???00?0????00??0?0??00000????????0??????0?0???????????0?001????
?????????0?0??????0???0???????????0???0??????0?????????????????
?????????1??????????????????????000??0??0?0???????????????????
???00??0?????????????
????????????000??01?0?1?0?0????????????????????????0????00?0??
?00?????0?111?0???1??0??012?????100??00010000100001???????0???
??????????0?????????????????0?000010??????????0???????????1?1?
??
Shenzhousaurus_orientalis ???0????????????????21000??10?0??1???000?
?00?000?0????????????0?00000000??0??0?1?12?0??1?????????????000?
?1???0???00000???0????????????????????????????010000000??1100010
0101?000001100??00001?0???????????????????0??1??1?0011?????00??
?0010??10000010????????00??0?00???0???00?000????????????????????
??00?????0??????0?00?0?100?0?0?0?????????????????????????????
???????????????????????????????????0?????0?00000?00000?0002?00
0??????????????????????????00????????????????????00000???????????0
1?00??00000??0?0000?00?0?0000000000??00???0??0??1???1?????????
??10????
???0?????????????????????00?00??00?00?0????0?????????????????????
????000???????00?????????????????010100000?0001?00??0?????????
????????01?????0?10??00??1??00?2?0000????????100?0????????00?1
0????00?????????00000000???00???????????????0???0??0???1?????????
??1???0
Ornithomimus_edmonticus ?00010?1101101?101021000?010101011000000
000000000100000??????0000000010?1001?1????????0?001?101100001

110001010000000000100100??????0112001200000?2020010000001100011
0101100000110010000011000100010000020??00?01111110121110000?0?
0010001000000001?????0001?000000002100000000?????????0?0?10???
000010110??????000000010000[01]0?0???????????????0??0?0?0?0?01?1
001?0?00?000???0000000??00??????1??00000??0000020?000?00000200
0000000000?0??0?????0??0??000?1???????0???0??0000000????????00
010000?00000?00?0000?01?0?0100000??0??00???0??0001??01?0????00
0001000000??01?00????0000101?0000??000000??0????0?????????1????
??????1????????????????00210???0?00000?010?????????????010?00?0
0001000011????????????????????10?00???1?????????????????????????
?1???????011110011111111?11000???????100??0???00?01????101?0??
?1??0?0000?10000000000????0000000000000100?0?????????1?????????
??10?01
Archaeornithomimus_asiati ??
??00?101100001
110001?100000000?0??????????010200?20000?0110[12]0???000?011000
1101011000001020100000l100??000?0000020??0000?10??10??1?1??00?
?0?001000???
?????????????????????0?001000?[01]0????????????????20?00?00000?0
?0??01?0?0010000?000000000000010000???00000??000??00???0000000
2000000000000?0000??00?0?10?000??????????????0???????0?0????????
00001???
???
???0????
???0??1?00001?00??00000?00020010?010?010100000?01011?000000000
00?000001000????001?101?001?11?0????00001000000110000l1??011??
?????110?00000110000000000??000000000?000?10??????101?0?1?000
?000????1??
Anserimimus_planinychus ??
???

1??????????????????0???????????1?200?2????00?202001000000110001 1
0101????001?001????????????????00002???00????11??10??1?1??00?0
?001000??
???0????????0??1???
??????????????????????????????????01000??00000????????????????
?????????10000??00??0??0??00?1????????????????????????????00
1??
???
???
????????????????????????????10?0??1?1??????????????????????????
1?0??????????11011011001??1?0????????100???01?00??????????????
???????000?10?000?????????????????????010?????????????????????
???????

Struthiomimus_altus ?00010?110??0??101021000201110 1?110000000000
000010??0001?01??00010000010200 01?1?????????001?1011000011100 0
1010000000001001 00??????0112001200000012010010000001100011010 11
000001?001000001100010001000002 0??000011111101211 10000?0?00100
0100000001??????00?1?0000??2210????0????????????0?0?1????000??
???0?00???00?0000?0000[01]0?0??????????????200?0?0?000?010100 1?0
?0010000??00000000000?100000??00000??0000000?000000000200?0000
0000000000??00?021000000?100220 00000?0??0?00?00?????????001000
0000000?00?0000?01?0?0100000000000 00???0??0?01??01?0????0000010
?0000??01?0000??0000101?00?0??000000??00?0?0??????????????????
?1000011010?00????00210???0?000000000????????????00??00?000010
00011?????00000000200?110101?10100100?01011?00000000000001000
1000001110011111 1?1?110000??0000100000011000010001101 00???1??0
?00001100000000000???000?000000000100?0?0?101?1110000?0000?101
0?

Gallimimus_bullatus ?00010?110110101010210002011?01011000000000000
0001000000 1?0100000000000102000 1?1?????????001110110000 1110001

0100000000100100??????0112001200000??2010010000001100011010110
0000110010000011000100010000020??0000011111101211110000?0?001000
1000000001?????00011000000221000?0000000100000?0000??00000?0
?10200??100000010000[01]0?0?????????????20010?0000000101001?0?
0010000?0000000000000100001??00000??0000020?000000000200000000
000000000?000?021000000?1002??0000?00??0000000???????000010000
000000??0?0000?01?0?0100000000000???0??0?01??01?0????00000100
0000??01?0000?00000101000?000000000?000?0?000???????????????00
10000111100000???00210?0?000000000000000????????000000?0000100
0011[12]000000000000020011?0101010100100?0101100000000000000100
?10001110111111011001?110001??00001000000110000100011010 0???110
000000110000000000??000000000000000010000?0010101110000010000?10
10?
Garudimimus_brevipes ?000?????01101????02?00020101000??000000000
00000000?00001?010000000000002?001?1???????0??0111????0001?10
001?100??00?????10????????????????????????????0000001100??????
??????0??001000001?000?000?000001000000?01??1110?2?1?0?00?0?0?
?000100000000??????0000100000010??00?0000?001000000?0000??000
0010110000??000000010000???0?????????????????????????????????
??00?000000002000000
000000000000000?021?0????0000????00???0??001000????????00???00
00000000002?0000?00?0?0100000000000???0??0?01??01?0????000001
000000??01?0000??00000000000000000000??00?0?000??00?010???????
001000000010???0???00210???000000000000000????????0?0?00?0000?
?00011100000000???????????????010100100?0101?0000000?000000100
?10001111??????001000?1?0001??0000???????????000010110100?????
?000???????????000000??000000000000000010000?0010??01??0011??00?1
11?0
Pelecanimimus_polydon ?00????????1????????2100?2?1??0000??000000?
0000???????????????0?000?0000???????000211??0001??000???0?0?111

?00????????????????0??0?00???120????00??0?2010????????????????
?????????????????????????????????????1001010????0?????0?????
0????10000??0?1?0????00????0000?0?10???0?0??????????0?0???????
?????????????00???0?10??????0??????????????0??0?0?0???0??????
?????????????????????????????1??00000??00000?????????????????
????????????????????0??000???????????1?????0?00???????????001?
00??00010??0?0000?00?000000000000??00???0??0?01??01?00?0?00000
11??000??????????????????????????00??0?000??????????0010000???
?0??????????????????0??00??00????????????????00000000?10?????
??0?0???????????????????????0?01???????????????????????????????
??????010?1??111??????0????0?2?0?????00001100??????????0?1101
??????????0?000?????????????????????????1???0??????1??????????1
11??1
Harpymimus_okladnikovi ?0??????????????????2100??????0??????00000
0000000??????????????000000?0000????1?1200??1???0???1???0?00??
10001?100000?00?001????????0?1?00120000000010?10??00???????1?
???????????00?00??????0??0?010000010??000001011????1?0???00?0?
0???001??0000??1?0????00??0000??11?10000000????????????0??????
????????0000??00?00000?0000[01]0?????????????????????????????10?
?01?0?0010000?000000000000???0?010?00000??0000000?0?0000?00???
??00000000?00000?00??11??0000?0?????????0???????000?????????0?0
01?000000000????0?????0??0?0?????0000?000???0??0?01??0?????????
?????????0???1?00??????0?????????????00000??00?0???????????????
???????????????????????00210??000?????????0?????????????0???????
000??0000?1??0????0000??2001?00?01?1??0010?????????0??????????
00?0??00011001000011??0??0?11?00????000???000001000????011???0?
?01???00000110?000000000???0?00???0?000?100?01??1?1?01??00??00
???1????
Beishanlong ??
??

??????????????????????01020012000????0100?????????????10???1?
?00??????000001100?10001000?01??000?0???1???0??1?1????????0100
0????????????????????????????210??????????????????????????????
????????????????????0??0???????????????20010?0000000101001?0?0
010?00?0000000000000010???????????????????0??????????200000000
000?00000?00????0??0?0?0????????0?0?????????????????00?01?????
??
??
??
???????????00000020011?????????????????????00?????0?00000?0???
00?????00??1?0???0??1??????????1000000?1??00?0?011????????????
?000100??00????????????0000000001?????????1???10?0??0??0???1??
Sinornithomimus ?0001?????1?0????1?21000??1010001?0000000000000
00000?000??0???00000000?00????1?1?????????0001?0?10?0???100?1?
1??0?00?0?0?100??????01020012000?0110100100000011000110101100
0001?00?0000011000100 0?000?010??00000101110012 1?1??00?0?0010?0
1000000001?????00001000000?02100000000????????????00??000001?
1?0???????00000001?000?0?0?????????????20010?00?00?0101001?0?00
10000?0000000000??01000010?00000??0000000?00000000020000000000
0000000??00??1100?0000?????????000???000000????????0?001000000
0100?00?0000?01?000100000??0?000???0??0?01??01?0????000??10??0
00??01?0000??000??????00000000000??00?0?0??????????????????10
000?????????????00210???0?000000000????????????0??00?00001?000
?????0?0??000000200?100?01010100100?0?010000?000?00?0??1?0?1?0
0111?1001110?1001?11?00???00001000000110000100?11??00???1100000
00110?000000000???00000000000000001000??0?101?011?000?0?00?101??
Qiupalong ??
??
???00??1100?????????
???01100?????????0??0001000002???00????????1?????????00?0?0???0

??
??????????????????0???
??20?0?0?00000?????00000
000?0000?000?02????????1????????0??????????0????????00????????
??
??
??
????????????????????????????10100?00?0001??????????000?010001?
?0??????????1??110????0?????????????????????1??01?????????????
?????????????0000??000??0000?00001??????????01??????1???????1??
Kinnareemimus ??
??
??
?????????????????????0???00001???00????????????????????0?????0
??
???????????????????????0??????????????????????????????????????
??0?????????0??????00000
0000000?000?02??????????????????????????????????????00????????
??
??
??
???????????????????????????????????0???????????????000??00000?
00??00???1????????????
????????????????????0???0000???001???????????0??????????????1??
Huadanosaurus_sinensis ?0??????????????????001??0210
??????00100??????????0??00???????00010100?00????0001001010?000
111?0?0000??1?00???????00???0?2010????00000?000010????00000003
00?01100010020201?1011?00?000??0?0?00?00????????????110?000???
?0000??00???0010?01?000000???00?0000??0100?0112000?000????????
???????????????0????0??????0??000?10?0????0????????0000?20000?

000?0?0100000?0?0?10000?00000000?0?00?????????1000??00?0?00?00
??000002??????????0???????????0?0??000?0????????000?10???1?0?
0?00?????000?11??0?000011?0000??001?0?0??000????00???0??0?0???
00???????????00???
?????????????????????????????????01??0???0?1???0????????????1?0
1?0???????0?0??0?0???????0?0001?1000111?0?0?0?0000000?010???00
0????0??0??????????000000?1110??10?1?1?10?0?0?0?10?00000???0??
??????0??110?0?0?0000?1???00?00000??000????0????????0???0?10??
?101????0???00????
Huaxiagnathus_orientalis ?00?0??????????????0001???1???0?????00??0
?????????????????????00?0010????????00010?1010?00??0???1??????
??0??????00???0?002010???000000000?00??01000000020?0??000100
201?000012?0???0??????0??0?010000000?0?011??000????00?0??00?0?
0010?0100000?0?1?00????0????0000??200???0000??????????????????
????????????????00?00?1??0?[01]0?0????????0000020?00?0?0?0?0101
00??????10?0???0???????????????????010????000??00?00??00000???
???000?0??00??0??0????100000000????????000????00000?0?00??0000
00?01??00000??0?00?0?00?1000000000???1???????????????????????
???10?00????????????????????????????????0?????????????????
???????????????????????02?00???0?????????????????1?0?00???????
0?0??0000??????0??0001100???1?0?000??00000??0?????????????0????
?0?0???0?0?0?00?101000??01?0?00?0?00001000?000??0???????0?????
10?????00????0?0?0?0000????0???0????0000100???????????????????
??000???
Sinosauropteryx_prima
000?0??????????????0001???1???0?????0000??????00??0???????????
00??01???????1?0001001010?000?01??100????1?0?0????00?110000201
??????00000000?10??010000?00010???0??0100201?000?11?0?1?00?0??
000?0?0100000000?011??0000??000?0??00?0?000??010000000?1?00?0
0?????000?0??20000?000???????????????0???????0101????????0000?0

??0?00[01]0?0??????????????0?00?0?00??010?000?????10?0???00?00?
???????????1??0100???0000000?00000??0?2?????00??0??00??00?0???
010000000????????000?10???000?????????0000?01???0000??1??1???
?0?1?0?00??0??????0????????????????????????00??0????????????
??????????????????????0???????????????????????????????????????
??02?00???0?1?2?0???????????000100???????0?0??0010??????0??001
11?0???110?000??00000??0?0???0??0???0??0?00?0???0?000000?11000
???0000?00?0?0?001000?0?0?0??????010????1100???0000???0?000?00
000??000???????0000100?????100?????????????000???
Compsognathus_longipes ?00?0???????????????00101?11100???00000??
??000????000???????0?0000010??1?00100001001010100?01??1010???
1?0?0???000?0120002010?????0?000000?10????0?0000001????00?0100
2010000012?0??????????00?0001000000000001100?00????0?0???00?0?
001000110000001?00000000?00000??020000?0000??0?0??0??????0???
0000????01?0??000000010000[01]0?0????????00?0?20000?0?0?0?0101
00????0?1000???0???00??????????????010???00?00?00?00?000?00???
???00??0?0000?00000?00100000000????????00????000000????????000
00?001?001000?1?0100?000100000000000??0???0??0?000?00?0????00
00000?00?0??01?12?????????000????????11?000????????????????????
????0????????????????????02000?0000102000?0???????0?000000?????
000??0000?0????0????????01???1????00?10?0??0?0?0???0??????0??00
00?0???0?000000?101000??00?0??0?000?0010???0?0????0????10?2??1
100??0000???10??10?00??0???00???????0000100?0010100??1?0??????
??001???
Juravenator_starki
0000???????????????00010??11100?1???01000?0000?0?????00?????0?
0000010???0????000100101??00??0???1???????0???????00??10000201
0????000000000000????000000000?01100???????????????????0?1???
00??????0?01?000000?10?000?0??0??0??00?0??????010000000?1?00?0
0000??0000???20000?000??????????????0????????????0??????000?00

???00??0?0????????[01]00002??00???????0101000?0?0010000??000?00
0?000???0?0????100???0000?0???00?000??2??001??1??000??0?0000?0
0100000000????????00???00?0?00???00??0?0000?011000?00??1?0000?
00?1?0000000000?00???0??0?00??00?0????0000000??000??01?10????
000??00???????11?000??00?0????????00?????????0????????????????
??02000???0?????0???0???????0?010?????????????????????????001
01?001?1?0?0001?01000????????0??????0?????0?0???00000?0??101?0
0??00?1??0?0???0????0?0?0??0?0?0???0????0?0?????000???0?000?0?
?00???0??????????00100?0?????00?1??????0??000????
Sinocalliopteryx
0000????????????????0001??0???000???001000?000??0??????????0?0?
000001???????1?0001001010100?01??10?????1?0??????00??1000020?
0?????0000000??00??010000000310?0??000110201?000012?0?????????
00???0??000??0000?011??000?1??0000??00????010?01?000000???00?0
000???00000??20000?0000?00?????0????00?????0????????????000000
01000?[01]0?0????????00?0?20000?0?000?010100??????10?0????0??00
???????????1??010????0000000?0000000002??00?0????00?0??00?00??
0100000000????????00??10?00000???00??00?000?01???0?010?0?0000?
000??0000000000???0???0??0?00?????0????0????10?00?0??0???2????
?00????????????11?0????0????????????0?????????0????????????????
???2?00??00?00????????0?????1???0000??0??000?00010??????0??000
010????1?0?000??00000??0?0???0??????0??0??0?0???0?000?00?10000
0??01?0?00?0?0?0010?0?0?0????0?0???0??0?0?00??0??0???00?000?00
00????00????????000101?????10???1??????????000???
Mirischia ???
???00?0
?0???????????????????????????????????????0??0?????0001?02000?0
?01210010001011 0????0??????????????1????????1????????????0010??
??
??????????????0?1?0???

?????????????????????????????????????0???00?0?000??????????
??
??
??
??0?
??000??????????????????????1?1????0?0?00?000?1??0?0???????????
???????????0??????0??0????0????0???????????1?????????????????
??????????000?0???000??00??????????????????0??????????0????1??
Sinosauropteryx_lingyuan ?0??????????????????00?1???11??????????00?
?????????????????????00???1?????????000?00?0?0??00??1????0????
0????????00?112000201 0?????0000?0?0?1??????00?0????0???????0100
201?0?1??1?0????0????0?????0??000000?0?01?????0???00?0??00???
000??01?0??00???00?000????????00?20000?000??????????????????
????????0??????0?????????0??0?0??????????????200?0?0?0???0???00
0?????1000???0???0????????????????????????????00????????0??????
????????00??0??0???01000??????????????00??10???0??????????00?00
?01??????0??2????????0??????????0??????0???????????????????????
?00???
?????????????????????0???0???0???????????????????0?0??0???????0?
0??0?????????0???0???10?1??????????????0???0?????????????0??0??0
?0???0???0?0????000???00?0??0?0?0??????0?????????????????0?????10
?????0?0????????0??0000???00?????????00100?????10??0????????????
00????
Ornitholestes_hermanni ?0002???0?0?00?1???0?010?01110001?100101?0
??1000001?01011????0000001000?0?000010?00101001?????011?010121
0000??000?0010??1?????????????????01?00??????????00001??1?0?01001
01000001??0?1???????0?????????00?00????00000?0010?00?0?0000?0?0
000?0100000001?01?00001000002?112000000010?0??000000?0?00???0
000?0?0000???000000010000[01]0?????????????????????????????????
?0?0?0010000?000000000000??0??0????1000??0000000?000?00000?000

0?????????0000?000?001??00?0????????00??0???0001?0?????????00?
?00?1?0000000000000000?000000000??00??0???0000?000?00?0????000
0000?0000??01?0000?00?0000100??00001?000?0001100?00000?01000??
???000000000000???0???0200000000000200000?00000?00000000???????0
001000002?10?00?0??????10010?01??000000000???0100????000?????0?
00?0000?0000???????????001??1010?0000???0???0????0?00??0010000
00?00???000000???000000??000?????????0?1000?1001?0001????0??0?
1010?01
Coelurus_fragilis ???
???0??0100100001210000???
??0?0002?????????????0?0??0?10001????0?????????????????????????
?01100?000001?0?0000110?0?0????0?00??00???0??????????0?0????0?
?????????????????????????11200?????????????????????????????????
?????????????001000??????????????????2??????????????01000?0?001
0000?00000000000000010000???0????????0000?????????00200000000000
0??000?????????????0000????????????0??????0?????????????0?????????
???
???
??0???????000010000
2?????0?000?0??100100?????????????0?0001?000000000?0000?????0??
???????????1??00???????????0000??000???0??00?0100????????1100000
00000?00??????????00000??00000001??????????0?1?0?11221111??????
Tanycolagreus ?000???????????????????00?0???????0???00110????????
0?00?????????????????????????10?00??????1??????????????0011?000??
???0????0?0??0????????000000000011?1000000????????????????????
???1100100000110000000100000000000000??00???0??000?????0?0???00
???????????????????1???1????[01]2000???0?????????????????????00??0
?0????????????0?00001000??0?0???????????????20000?0000000101000?0?
0010000?000000000000000100000??01000??00000???????????00200000000
000000000000?00100?00000????????0?0???????????????????00000????

?001000???????????????????0??????021000000?000?0?????????????
???0??0??00???????0?0?000??00???????????????????????????????
????????????????????????????????????0?????????????????????0
0001????????00110010010000000?????????00???0000000000000000000?
000?????00100000000??0??0?0?100?10000000000000101000???????????
??0000000000?????????0?000000000010?????0??0?10010122101 1???1
??
Tugulusaurus ???
???
????0???????????????????????????????????0?0???????????????????
?????????000?0?100??00110?0?1????0?????0?????????????????????
???
???
???2000000000
000???01?????
???
???
???
???????????????????????00?????????????????0000000000000?0??????
?0?????????0??????????????????????????000001???1?????????????
???????????????????????0?????00??????????????????0001??1????1??
Zuolong ?0000???????????????000001011 1?0???000?000000110
0000?????1000??????????????????00010?101??1?010001000????1???00
0000?0000??????????????????000?0??????0?????00?0100???????????
??0110011000 0110?00?0???00010?0?00?????0?0100??????00???0???00
11000000??0???00??1???0??0002000??0000?????????????00??0000000
??????????010?0?10??0?0??????????????????????????????0??????
10000?0000000000000010000????????????????00?0?0?00?0020000??????
???0000?000?0?1???0???????????????0???000100?????????0??00?????0
00000000000?00000001000????????1???0??00?0000????????????????

?0?????0000?00000?0?000??10??0010?0001101?00000???????????????
?????????????????????????????????0??????????100???????00000??0
???000???0??????00000?????0?00000?0?0?0??0000000000????000?000
?00??0????0?????000??00?0???0???????????000011?????0?0??0000?0
?00000??00???0?0???0000000???00??0??????1?0?10??000??01?0?01??
Bicentenaria ???????????00?1??????0?0??????00??00???????????
??00????????????????????????0?000000001????????????100????????
?0?????????????????????0?00???????????0?1????0??10????????????
????0???00000?10???0?110???????0??????????1????0??????????????
???00??0???0???????????????1???0??????????00????????0??????00?
??????????0???0010????0???????????????2???0???0???0?????0?0?00
1??????00000000?0??????????????????????0??0???0????2000?000000
00?????????????0?????????????????????0????????????????00??????
??0???????????0???00??0???????????????????????????????0?000000
0???????????????00000000?1????????????????????????????????????
?????????????????????????1?2?00??000??0??????0????????????????
???????1?00??0????00????????0??0?????????0000?02???00?0???????
00????????0?????00??????0???00???0???????00???00?????0??????00
??00?0??????0??0?????00?????00???????????0?????0?0??0?1?0?????
Kileskus ???????????????????0?01120111??????????????????
??????????????????????????????00000?101???????????????????????
??
????????????????????????00?00??0??????????????????????????????
1?0000?0???0??00??????1????????0??001?????????????????????????
??
??
???1
011201???000?0000?0000?0??????????????????????????????????????
??
?????????????????????????100010?????????????00????????????????

??00?????
00??????????????1??????0???????????????????????????0????????
?????????????????????????????????1?????????????????????0?????
Guanlong ?000201000000?010[01]10001120111200 0?0011000000
1000000000?10????0000010?00??20000001010?100?0000000011100
000000000?0???00??????000000100000?01000000030000100001 00200
000001110010000011000?0011000010000000000000000000000?0?001
00011000000001100?00?1110110 2?1120000?0010????0000?000?00??00000
00??0?0????00000001000 0?0????????????????20000?0?????0101?00?0?
0010000?00000000000000 10000???01000??0000000?000000000200000000
0000000000000?00100?0000020?20?000?00??0001000???????000000000
000112010?1000?00?1000001010000?003??0000??0??00?1000100000110
0010??01?1000?00000000000000?1000011?000000?00000????????????
020?010001000000000000????0?000000010???????010100???????000010
000210000?0?00000100010000000000000001000000?0110000000100000?0
000000?0001100000100?010010?100010000000000001000000000 0?00100
10000000000000000??000000000000000010100???1?0011?1001??1000001
??
Sinotyrannus ???????????????????0?01???101?????????????????
??????????????00??0?1????????0?0000101???????????????0????????
?????????????????????????????????????0??00??0?0?000??????????
???0??0?????00?????????
1?0000?0??100??0?????11????????00?001?????????????????????????
????0??
??????????????????????????????????????00?000?0?0?????????????
???0????????????00??????
01?2?1??10???0??1?00????1?????????????????????????????????????
??
??????????????????0???????????????????????????0????????????????
??????????????????????????????0001?????????????????????????????

?0?0??????0??????????????0?1????????????????????????????0??????
??00????
Proceratosaurus_bradleyi ?00?2??????????????00011?01112000?00?????
?????00?00???????000?00000010?00?1200000001010 11??????????????
???
???????????????????????????????????????00??0????0?0??0?????
??????110000?0?1100?00?11?0?1?2??????00000100000?????????00???
0??00??10?0????00???
???
??????????????????????????????????0???????0??????????????????
00?0010112?10?1000?00?1?0000101???0?????????????????0?100010000
0110001???????0???0???00000000??0?0????????????0000??000?????0
??0?0??????00100?????0001???10?00000001000?????11000??????????
???
???????0?00?????????????1????0?0??????????????????????????0??0010
???1?0?1??????????????????
000???
Dilong_paradoxus ?0002?1000000???0?100011??1112000?00110?0000100
00000100???????00000000?00??20000001010?1?0000?0001?0??1000??
???0??0?0?0??0???????0000001100?????0000000?00??1??00110201?0?
0001?00?000?0??00??00010000100?0000?00000?0?00000?000?0?001000
1000000001100?001111?110201?20?000000????00000???0?0???0000010
110000???000??00??00????0??????????????20000?0?0?0?0101000?0?00
10000?0000000000000?0??0????1000??0000000???0?000002000000000
0000000??00?0?1000?0000?0?20?00???010000100????????0000000001
0002?11?1000100?0?00000000000103??0100?00??00010111000011000
10??00?1100??000000000010????00000?000100???????????????????02
0?010?010?0????01001??10?10002?00?11?????0111000000010000 01000
01???????0000011000 1????10000?0001??000???0110000000000000??010
000000??11000????1?01?01001?????00??00???00???00000?00100?00?0

000010?0?000000???00??0?000?0001?100???1??10112????0??0000???

Eotyrannus_lengi ???????????????????000120??12??0???110???????

???0?????????0?000?0?0??1???2000000101012?00?????01?0??1?????

????????????????????00000010????????0?0????????????????????

???????????????????0???00000?0?00???00?0????00?0?????0?????0?

1?000?00??10??0121???111?0?211?0000?0???????????????0??00?0010

0???00??0???0?0010?0??0???????????????200?0?0?000?0?0?0?0?0?00

10000?0000000?0000???????????00??????????????0??????????????

??????????????0???????????????????????1???????????0?00?????1

?00101???????00?0?01????01000?1010??1??0?00?00???????????????

0????????????????????01?1??0?????????????????001??????0?00??

??????????????????000?1002110100??????????2??00???00???00????0

?????????00??011011?000??????????????????????????01??00?0010

0?000?0???0??????1?0??0?00????1000??????0?00????????0000?00???

0000100?00??0??????????0000???0?1????0??????011??????1???0?1?1

Juratyrant ??

???????????????????????????????????????0?00?0000001?10??00

000??0?????????????????????????????????00?00?010000110101100

00011?01???0??10????0?????????????????????1????????00???0010??

????????????????????2????11??????????????????????????????????

????????????0?00100?0?0???????????????????????????????????????

??????????????????????????????????????00?000000000?0?00?000??

00?????????????1??????????????????????????1?0?????????????????

??

??

???0????????00?1000

11?00100?0????????????????10000001100100000?????110000 1?????????

???????????0??01??1??1?????000?????????????0??????????????0000?0

?????????000000??000??0000?????????????1??1??????11??0????1??

Xiongguanlong ?0002???00000?????1?000220???0000?001101000?21

000000?0001??000???????????????00000?101??20000000000000111000?
??0?12?01000?????????
??????????0000110??????????????????0??????0?00?????000????????
?1?000000???00?0?1121?2102011???0??0000?00?000??????00???0??00
0????????????00?00100??
?????????????????????????????????????00?0000000??20000?????
??0????????????????????????????0???????000100??????????????0010?
10001????1?00?01?0?01?0000100??0010?0100??0??0??0?????0???0100
0?0???0?0100???001?111110010????100?1?11??00?100?????????????00
1001100010?1??????????????????????????????2?000??000000011000
011???????????????????????????111??001???????0111012??????????????
??00??0?????????????1??1?010?10??????????????00??????000?0??00001
???????????000000?????000000???????10????1??????????1???0?0?01??
Dryptosaurus ??????????????????????20????????????????????
????????????????0?0?0??2???????0000101?1?????????????????????
???0???00???????????????????10???????10?0????????????[01]?0?0?2
???????101?01000100010001000??2???0???????????0?0?????????0?0???
???????????00???????????????11????0?????????????????????????
??????????0?????????????0?????????????????????????????????0?0?
0010000?00??????????????????????????????0????????00200000000
0000??000??0???00?01????
???????????????????0??
???
??????????????????????????212?1?1????????????????????????????
??????????????????10?????????????????????1?0???012?1?0111?????
1????0??????0??????????????0???????????????000000?????????????
???00?1???00?????????00??0000?00001????01??????????0?1??00?0?0
??
Appalachiosaurus ????????????????????0?0?201110??????120????????
?????????????00000?000?0?????????0001010?2????????????????????

???0?0?00???000010120?
10???10??0100010001000100000 2?0?00??????0????0????????0?00100?
11000000?1???1?021??????????????000??0??????000?????0?????????
??0000???0????????????0???????????????????????????????????????
???????????????????????????????????????00????????0020000000000
00?0000?000?00?????????????????????????????????????00?????1???
?????000?000?01?010111?101??1?0020?01?10??0?0?????????????????
??000000010100010?00??
???????????????11?110??1?????????0??1?????2?0?????????????????
????????0??????????????????????????????1?01110???11111111021?0
000????????????0?????0?0??00????????????0000000???00??0?????0?
??????????????????00?0??00000001???0???????01?????1??00000??B
istahieversor ?1102?????????10?2200002?01110000?001201000?2110
0000??????????00000000?20??2?000000101012??0??????????????????
??
????????????0??????????????2???????10??00??00?0??0???????????1
20000011?00?00213?0?112??????0000000?????????10??0????00001??
?0?0????00???????????????????????????????????????00???????????
??
????????????????????????11??????????0???????????????????0?1011
001000?1010101?01011101010101002010001001???10000??00000020000
211000010000??111111?1?1001011001211111?01000?01001111??????11
?111101?1011111100?11212101100112?10??2?00???111??????????????
???0111?1???????????????????????????011?????0110111?120010000?
???????????1??1?0?0???????????????????????????0??0????????????
????????????????????????????11001??????1??????????000?11
Albertosaurus_sacrophagus ?1102??000000?1002200002201110000000120
1000021100000?0001000000000000000020??2000000001010120????0?10?00
1?100?0???000??0????0???????00000001 0000???0110010312001 10000
00101200100011 0??0?00?1000??001000002000000010000000000 0?000?

0?0010?0120000000011000?00213101112102011000000000???00000100?00?
?00000100100000??0000000010000?0?0????????000002000?00000?0001
001?0?0010000?000000000000010??0?????000???00??00?000000000200
000000000000000?00??0100000??020100?000??0??001100?0110?00000
01011101100100000[01]1001[01]100101110102011100211[01]1[01]1101?0010
0000010001020100200110010001111011111111010110011111111000[01]00
0111[01][01]11110000101101111111101111111001112121111100?12?10?12101
1???????1?????????????????11111020111111?10211111102111101??1??1??
?111111111122010000000??00000101?011010?100010?000??00?00000010
0?000000??0?00??110??00000000??0000?0000000001011001111010111??
??0100000011
Gorgosaurus_libratus ?11020?0000001100220000220111000000001201000002
110000010001000000000000002001200000001010120?000001000010100
000?000000000000?00001000000000100000010110010312001100000001012
0010001101001000100?00010000020000000100000000000000000?0?0010
0012000000110001002131011121020110000000000000000100000??00000
10?10000??0000000010000[01]0?0????????0000020000?00000?0001001?
0?0010000?000000000000001000010??1000??0000000?000000000200000
0000000000000000?0010000000020100?000100??00110000110?000000101
110110010000[01]1001010010111010201120021101[02]1?0100010000000100
010201002001100100011111011111111101011001111111000100011111111??
00110110111111110111111100111212111100112?10?121011???????1????11
01????1?0?01111020101111102111101021101?011?1?2?11111111?22010
00000010000001011011010?100010?00001000000000001000000000000?000
0110?000000000??0000?0000000001011001111010111110??0?00000011
Alioramus ?01020100000011002200?0?201110000000012010000211
0000010001000000000000000200?20??000010101?000000001000011100000
0?00?00?0??000?????????????????????????????120011000[01]001012
?010??????001000100??0?01000?02??000??10??000000?0?0000????010
??1100000011000 1?0213101?12102????000?000000000000100000??00000

100?0000??00000?00100?0?0????????????????????????????????????

??00?0???000??200000000

0000000000?0??0?1?0????0020110?00???0??0011000???????0????0011

1?2????001110111110?0111010100120121001021110101110001000010200

0010000001011011120111111100101100111111?1011100110111010001101

1011110110111011110011121211110??10000???000001000011110?101221

011111?????????????????111010??????1?1111121??00?111?12?010000

0??????0???0?1?1?010??000???????????0????01?0000?000000?0?????

???????0000??0??00???0?000?1??101111?1?1??1011??0?000?11

Teratophoneus ?1?0???0???????0??2??????0111?00????1201???????

0000????????????00??000????????????000010101??1?????????????????

??0??????????????????

???????????????0??????????????????????????0????0????????????????

1?0000?01?????????3?????????0???000?????????????10??0???00?001?

??0?????0???1?????

???

?????????????????????????100????????????????????????????????????

??????0????0?01???????????????2?021?0001??10???????????0???01?1?

?????0???01??1?1??21?111?10011101211??????????????????????????0

11?1?120?1????1??1100?11??????????11?????????10????????????1?????

?????????????1?01???1?????????????????????011?1?1???????????????

000???????????????????0?0?????????????????????????????0??0???????

???1??????1??????????00??1?

Daspletosaurus ?110201000000?100220000220101000000012010000211

0000010001000000000000002001200000001010120100000100001?100?0?

???000??0????0???????000000010000???11100103120011000000101200

1000110??0100?1000??0010000020000000100000000000000000?0?0010?0

020000001100010121310111210201100000000???00000100?00???000010

010000??000000000010000?0?0????????0000020000?00000?0001001?0?00

10000?000000000000010??0????1000??000??00?00000000020000000000

00000000?00??01000000?020110?000??0??0011000110?0000001011101
20011020110021110211101021012012101010[12]11111111111110110102000
0211001011[01]1121121211111100?111012211111101110011011111[01]1001
111101111011111111111001112221111011112?100121011000111011110211111?
??????111111?211?111?112111??1??111?1??1????2?11????11?12???0000
0??11000????1??1?010?1000???000?1000?0??????0?000000000???0011
000?0????????????????????????111011110??1??????????000?11
Tyrannosaurus_rex ?1202010110001100220000220101000000001201000021
100000100010000000000000002001200000001010120100000100001010000
0010000000000000?0????00000000010000???11100103120011000000010120
010001101001000100010000020000000100000000000000000?0?00100
00200000002100010121310121210201100000000000000000100000??000001
0010000??000000000010000[01]0?0????????0000020000?0000000001001?0
?0010000?00000000000000010000????1000??000??00?0000000002000000
00000000000000?0010000000020100?000?00??0011000110?00000011111
112001102101[01]0[123]0110211101021112010??1012101111011[01]11011
110210102110010111111211[12]1211111110011112123011111111111011011111
1111111011020111111111111121122212110111211111211211111110111110211
11211111111111102110111111211111102111111112211211111111111122110000
0001000000101101101 0?1000100000??000000000110000000000001000011
00?10000000??000000000000000101110111101011110110100000011
Tarbosaurus_baatar ??2020?011000?1002200002201010000000120100002
110000010001000000000000002001200000001010120100000100001010000
00?1000000000000???????00000000010000?0111100103120011000000001012
0010001101001000100010000020000000100000000000000000?0?0010
0002000000021000101213101212102011000000000000000000100000??00000
10010000??000000000010000?0?0?????????????20000?00000?0001001?0?
0010000?00000000000000010000????1000??0000000?000000000200000000?0
0000000000000?0010000000020100?000?00??00110000??????0000111111
12001102101113011021110102101201 0??10121111111011111011110 21010

21100101111121121211111100011121230111111111101101111111111111011
02011111111111112112221 2?1011121111211211111 1?111?1021112??1??1?1
11111021101 11?1121111110211111111221121111111111122110000000100000
01011011010?1000?0000001000000000110000000000001000011 0??100000
00??0000000000000010111011110101111011??00000011
Timurlengia ?????01001??????021?????20111??????????10000???
????0??????????????????????1200?0000101?12????0?0?01001110????
???0??000????????????????????????????0?0????????????????????
???????????????????????????????????????0???????0?0?00??????????
1?0??????1??0???0???1????????????????????????????00?????0??00?
???????????0??00100???
??
?????????????????????????00???00???0???0010????????????010?????
?????00??00??0100?01?100??????????????????????????????????????
????????????????????????????0?01100?1????????????????????????02
??01000?0???100??????????21?11?1??11??????21000????????01??00?
11?????0?0??
00????????0?????0??????10?1000?????????00????????0???0??000?00
????????????????????????????????????0????1????????????????0???0?
Zuni ?????????????????????012??111???????????000????
????????????????00??000??1??12?0?000?101???????0?010000111?????
???0????0??????????????0?00???????????????????????????????????
?????10?000001100000001000002???00???0?????0???0??????0?0???0?
1?000??0?1000?????????1????????00?0?0?????????????????????????
?1?????????00?001000??0???????????????2??????????????0?????????
???002000000?0?0
00?0000?000?0???????????????????????????0?1??????????00?????????
0001??1??00???0?0?0110?0??????????????????????????????????????
??????????????????????????????0010010?????????????0?????????????
???????????????????1??????21??1????11?????1?1000????????0111?00

11?????0?????????????????????????????????0011?11??101?1110?21?
?0?0?????????????1??????0??000???????????00000?1????0??0000000
0??????????????????00000000?000?1?1?????1???0???011?0?1000?1??

The scores for *Sinosauropteryx lingyuanensis* and *Huadanosaurus sinensis* in the phylogenetic analysis of Cau, 2024.

Sinosauropteryx_lingyuanensis ????????????????????????????????????
??
??
??
??
??
??
??
??
??
??
??
??
??
??
??
??
??
??
??
??
??
??
??

???
???
???
???
???
???
???
???100000?10000?0
1?0?00?10???00???010?1???0????0????01????1?????????????0?????
???????1??????????0?1??
??????????00?0?00000000??0??0??0??????????????????????0??????
????????????????0?10011???????????10???00?????0???????????100
??0?100000101??0??????0?1?0101???????????????????????????????
???????????????????????????0000?????0?0?00001?0010???????????
????????????????????011?0?0?0???10000?11?00110???01110????????
???????????????????????????0110?010?000011?011001011100?0110010
00?000???0????????1110???0?1?00?10?10??00??????????0??????0??
???0??????0?0?0???????0??0?0??0???001??????0????00?011????????
???1?00???0???1?0??1??0??0??101??????00????1????0???0??1??00?1
?????0????????????????????????0??001??1???????????0??????????
???1??????????0????????0??0????????????000???000???????0??00?
?????0????00?????0?????????0??????????????????0??????????????
?001??????????0???????0??00?????0??????000???????????????????
0????????0???????????0???????????00??????000????????????0????
????????????0?????????11???0?????0????0?101???????????????0??
????????????????????????????????0?????????1000?????????????0?
???0??????????0??????0???????0??????????????????????????????1
???????????0???????00?????????00?0???????????????????????????
?????????????????????????0??????0??0???????????0?????????????
0?????????0????????????????????????????????????0??0??????????

0????????0????1????????0???????1????0?????????00???????0??0?00
???0011?????????????????00?????????????????0??????0??????0?00?
0??0000000???0??????0???
?????????0????????1???????????????0?????????????0?????????????
??????????????1?0??????????????1????????????????????????????0?
???????????????0???????????00??????0?0???0??????0?????????????
???????1???1??????????0?????00?????????0???????0??????????????
???0???00????????1??????0?????0?????????01??0?0??0??????????00
0???0???????????????????0??????01????????????????????????????0
??1?0??

Huadanosaurus ???
??
??
??
??
??
??
??
??
??
??
??
??
??
??
??
??
??
??
??

??
??
??
??
??
??
??
??
??
??
??
???????????????????????????????????????100?0001?0?01?1?0?0?110
???000??010?0???0?????0000??0?0?111001????????0????????1?0????
???????????????????????0??00??????????????????????????????????
?00?0???0?1000????1???001??????????????0??001?0????100?1?1?010
?010?00?00?011?0101???0????000?01??00?01?1110?10?00100?001??0?
0??1??1?0?0??0?0?00?????1????10101?0?0??00??11??????00100??100
1??????0????????????????0?0????0?0?????0????10111??0?101??1?00
111?11?0??0011?1?0?????1000??100??0??011?????????????????????
???01000?0?00?
?0???????0?000?????1??1?0??1??00?????0????1????0???00?????????
0?01??????0?000000????0?0000?0?0??0???0???????1???1?00??0?00??
?00?????0???????0?0???1???????????????1???0?0??10?0??10????0??
???1???????0?0?1????00?????1??1????1??0??????????????0????0??0
??????0????00?100??0????0??00?0?0???1?00????0?????00?0????0001
0?01?????0????????10?00?0????0?10?????????0???????????0?10????
??011?0??0???0??????????0?????0??0?????0?0????????1??00?0?????
?000?????0????????0???000?0???????0?0???0???????0?0??0??0000??
0???0?????0?0?00???0??????0????00?0????10??????????????0??1??0
?0???0?00??0???0?00?0?0??0?0?000?01??????????0?0??1?0?1???0???

???1??0???????????1?????1?????0??000?0???????00??????1?0?00???
0??00???????????0????000?0100????1???0?01?0???0???0??????00?0?
0??????000??????000???????000?00???0000??????????0??00???0????
??0?????0??10???0???????10?0????00?????????0?????????00??100??
??????1?0?0???????????????00??000????00?????????0?1?0?10?0?1?0
??????????????????00???????00????0?00?0?0????????0??0????010??
?0?????0?0??????0??0??????00???????00???00?0?00???????0?01?0?
10??0001?01???1????????????????0??????????????????????????000?
?0??????00??00?????????0????10???????????010?000??????????????
???1???????????????000?1?100?00?001??0????00?????????????????1
1????????00??????????0?????0??????1?0???0???0?????????????0???
?????????????????????0?????0?????0???????????????????00???????
????????????????????0??????????????????1????????????????1?0?0

Fig. S1

Additional photographs of the holotype of *Sinosauropteryx lingyuanensis* sp. nov. (IVPP V 12415). a, cervical vertebrae. b, caudal vertebrae. c, shoulder girdle and the forelimb. d, foot. e, pelvic girdle and femur. lc, left coracoid; lh, left humerus; li, left ilium; lis, left ischium; rc, right coracoid; rf, right femur; rh, right humerus ris, right ischium; u, ulna. Scale bars, 20 mm.

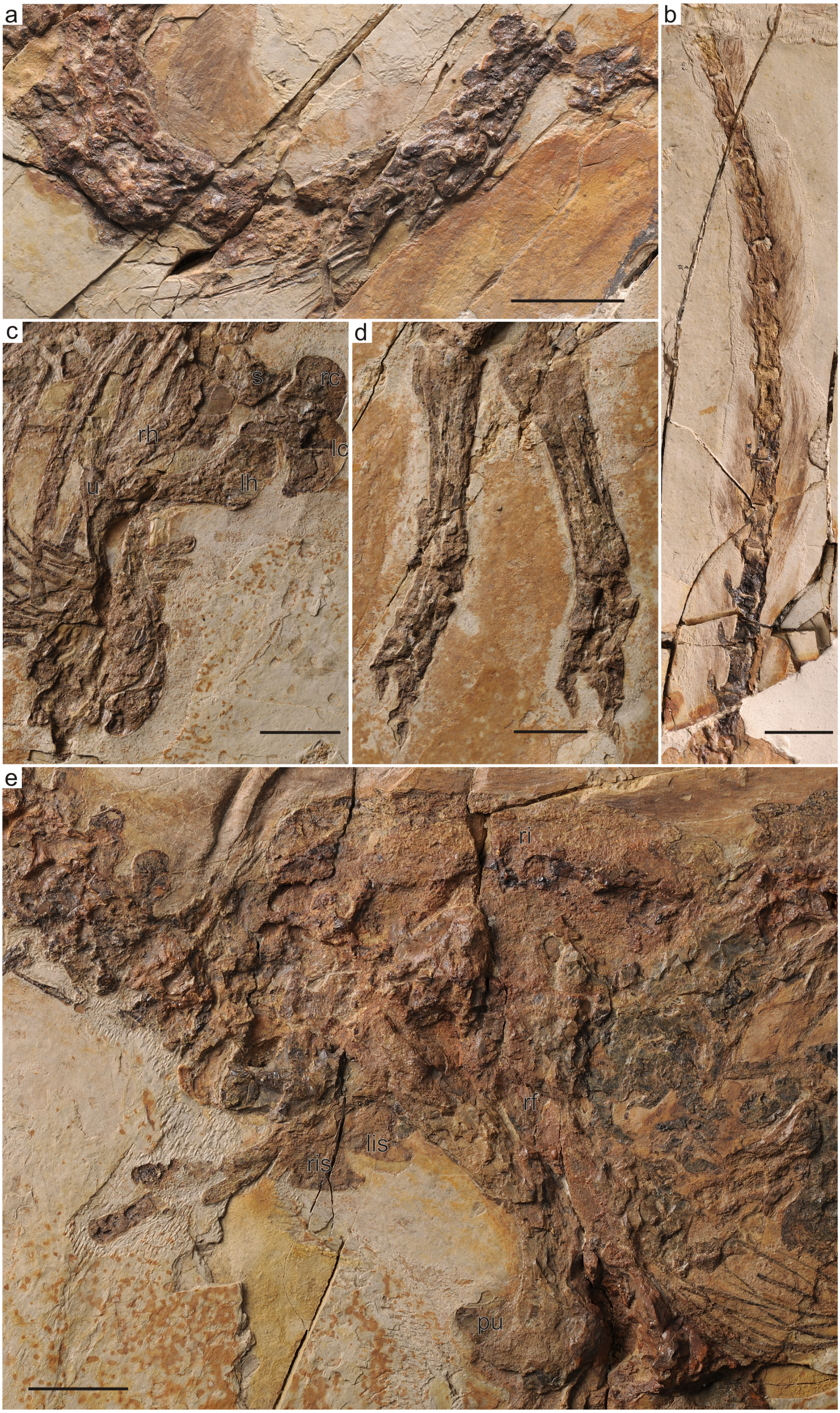
a
b
c
s
rc
rh
lc
u
lh
d
e
ri
rf
lis
ris
pu

Fig. S2

Additional photographs and line drawings of the skull of the holotype of *Huadanosaurus sinensis* gen. et sp. nov. (IVPP V14202). a, photograph of skull in lateral view. b, line drawing of the right lacrimal in lateral view in lateral view. c, line drawing of the right maxilla and premaxilla. d, maxillary tooth in labial view. The dark gray shadings on maxilla and lacrimal in c denote the antorbital fossa. apo, accessory pneumatic opening; con, concavity on the maxilla; lre, lacrimal recess; mf, maxillary fenestra; pf, promaxillary fenestra; snf, subnarial foramen. Scale bars, 10 mm in a-c, 1 mm in d.

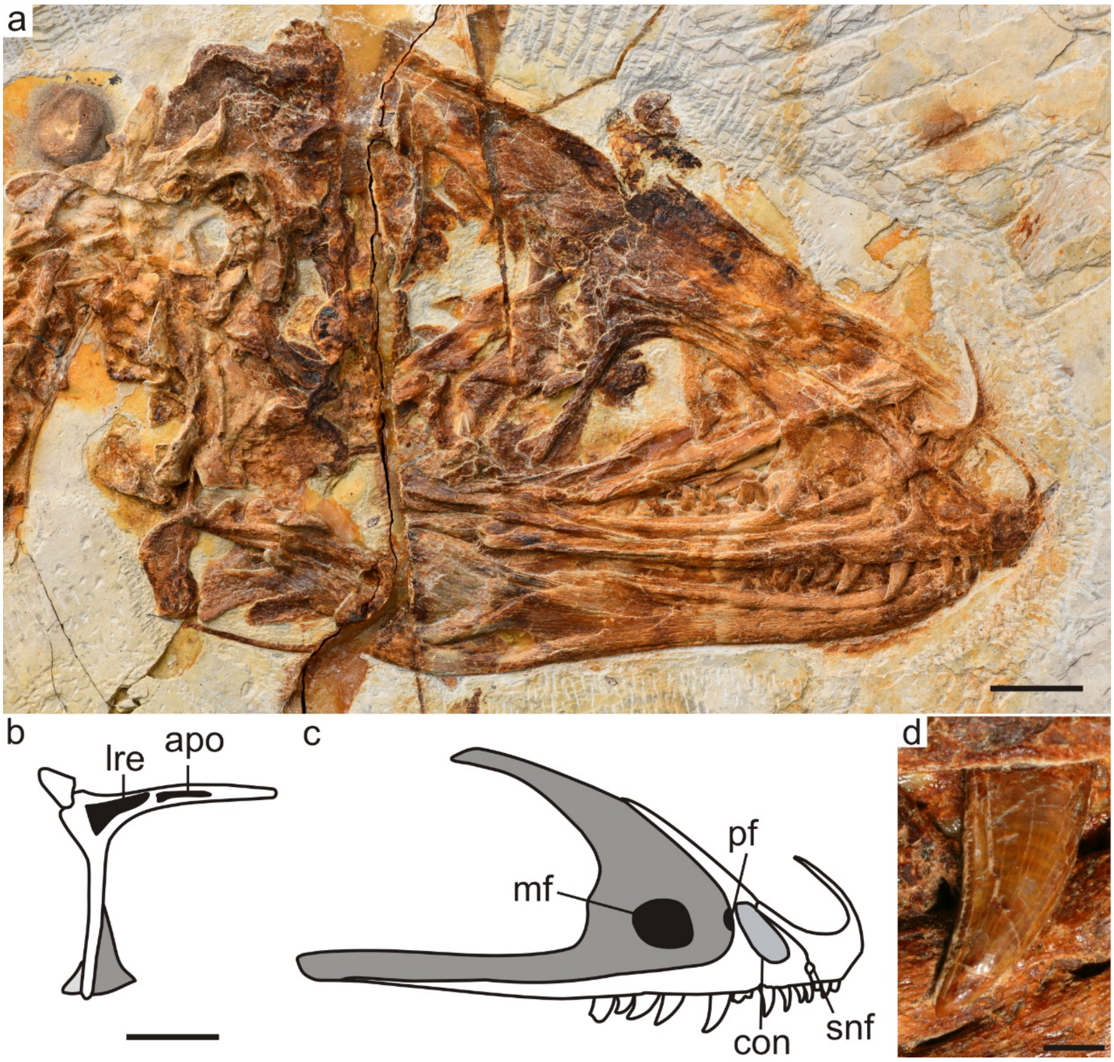

Fig. S3

Additional photographs and line drawings of the vertebrae of the holotype of *Huadanosaurus sinensis* gen. et sp. nov. (IVPP V14202). a, photograph of the cervical vertebrae in lateral view. b, line drawing of the cervical vertebrae in lateral view. c, photograph of the dorsal vertebrae in lateral view. d, photograph of the anterior dorsal vertebrae in lateral view. e, photograph of the caudal vertebrae in lateral view. acdl, anterior centrodiapophyseal lamina; idf, infradiapophyseal fossa; ipf, infrapostzygapophyseal fossa; irf, infraprezygapophyseal fossa; pcdl, posterior centrodiapophyseal lamina; pl, pleurocoel; pp, parapophysis. Scale bars are 10 mm.

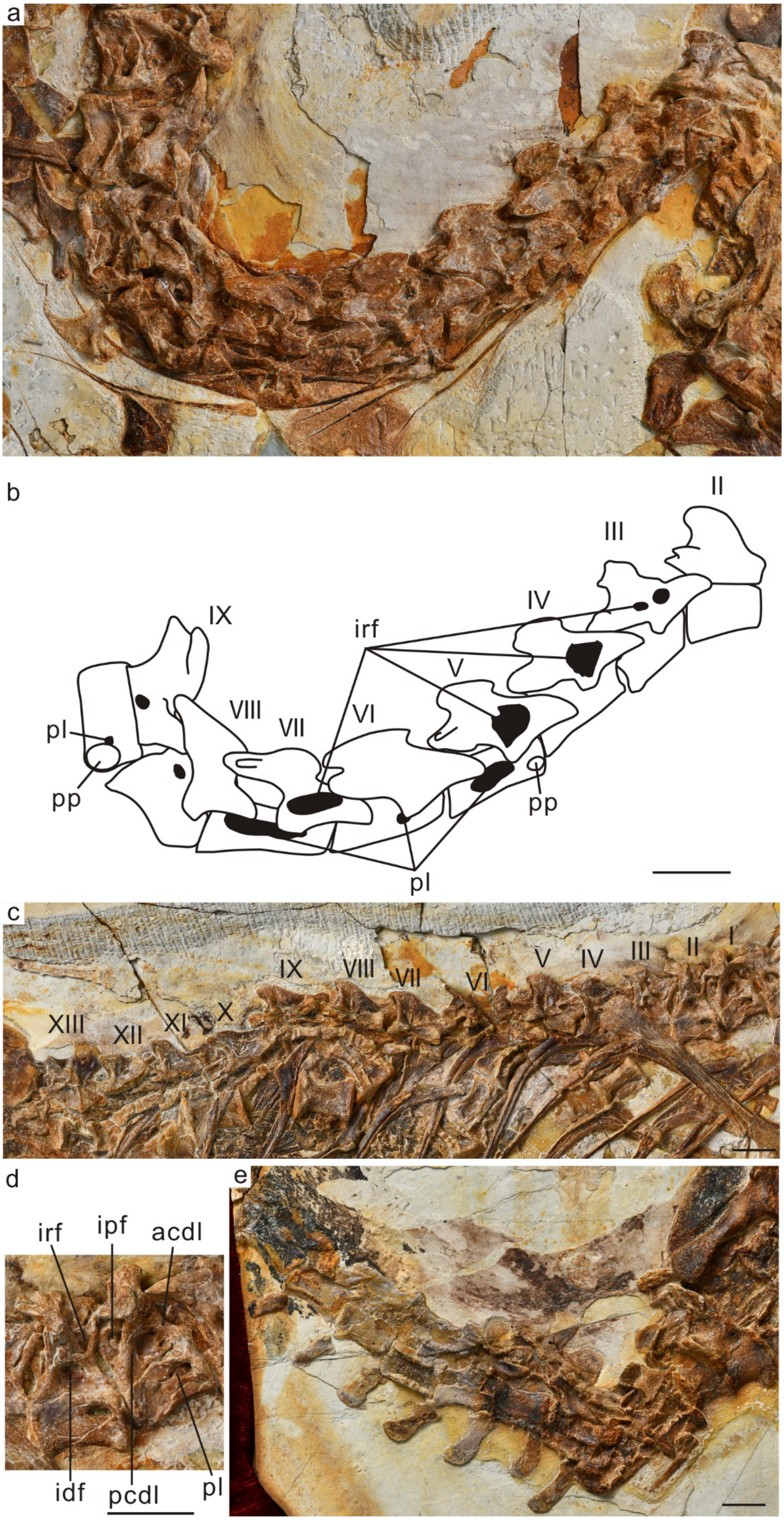
a
b
II
III
IV
IX
irf
V
VIII
VII
VI
pl
pp
pp
pl
c
IX
VIII
VII
VI
V
IV
III
II
I
XIII
XII
XI
X
d
irf
ipf
acdl
idf
pcdl
pl
e

Fig. S4

Additional photographs and line drawings of the pelvic girlde of the holotype of *Huadanosaurus sinensis* gen. et sp. nov. (IVPP V14202). a, photograph of the pelvic girlde in lateral view. b, line drawing of the pelvic girlde in lateral view. avp, anteriorventral process; bf, brevis shelf; cdf , cuppedicus fossa; mrid, medial ridge on lateral surface; op, obturator process; sac, supracetabular crest.

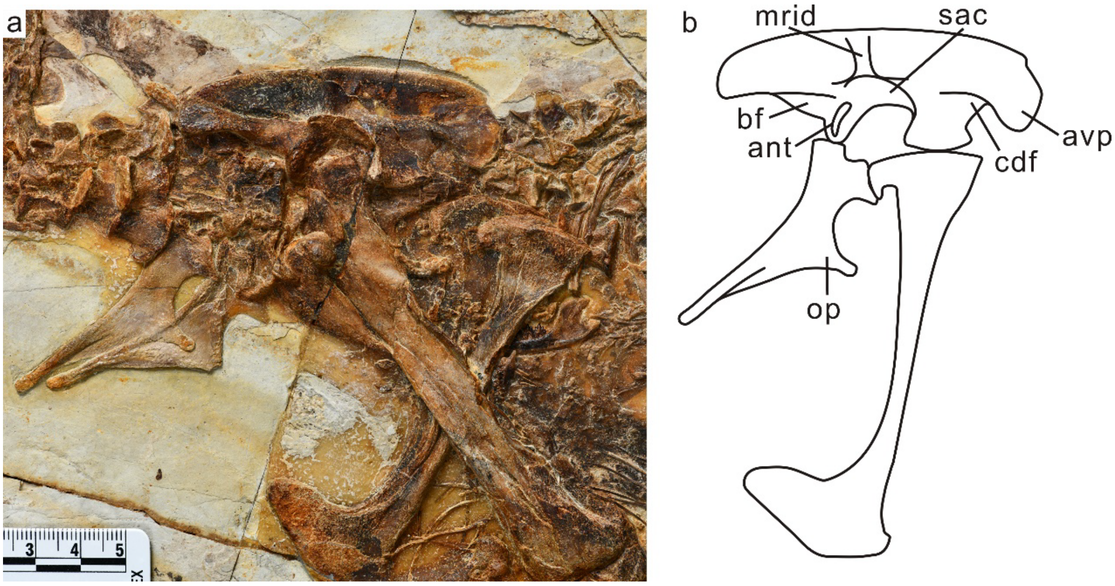

Fig. S5

The reduced strict consensus for the coelurosaurians used in Time-calibrated phylogeny of Sinosauropterygidae shown in Fig. 3.

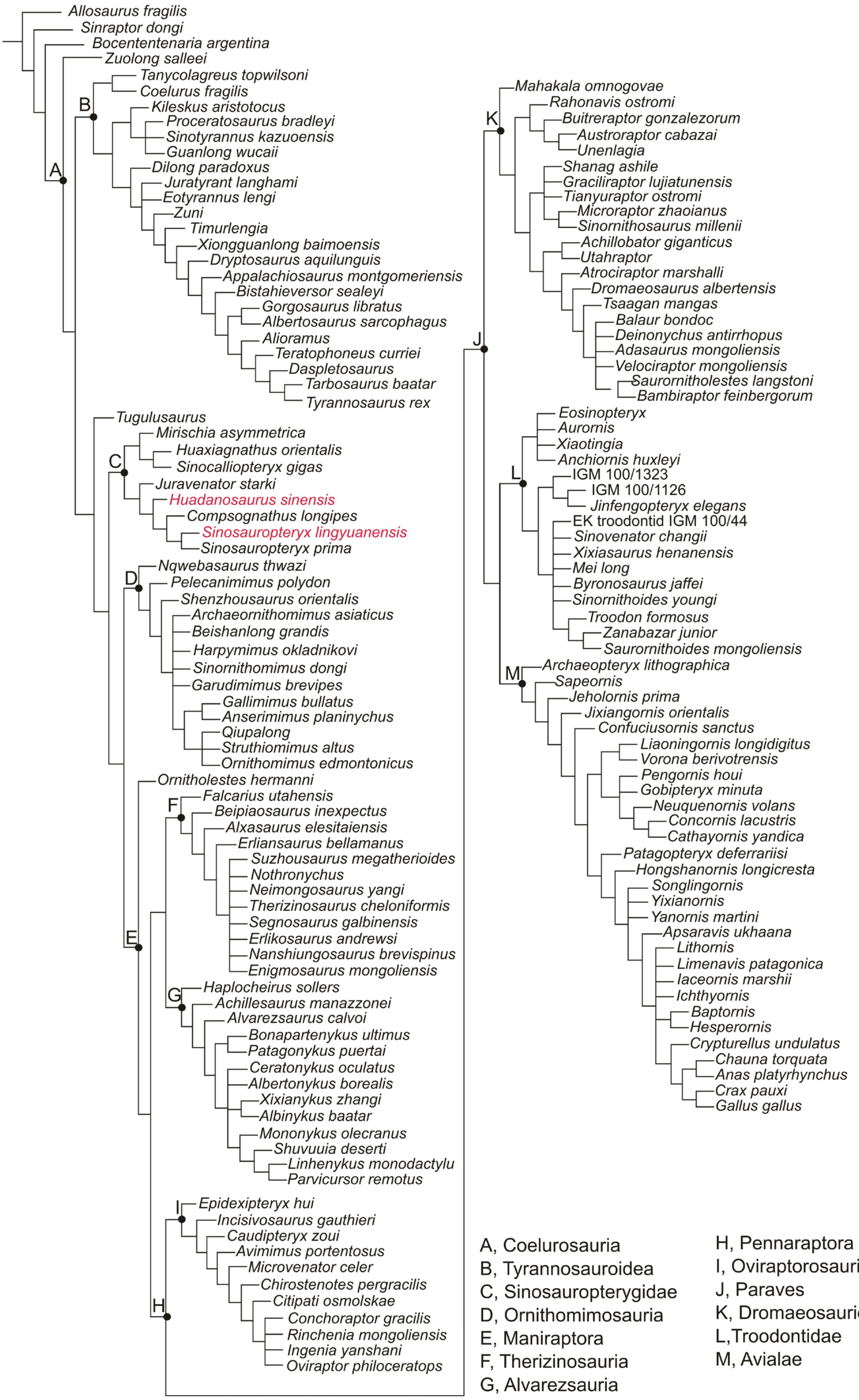

A, Coelurosauria
B, Tyrannosauroidea
C, Sinosauropterygidae
D, Ornithomimosauria
E, Maniraptora
F, Therizinosauria
G, Alvarezsauria
H, Pennaraptora
I, Oviraptorosauria
J, Paraves
K, Dromaeosauridae
L,Troodontidae
M, Avialae

Fig. S6

Complete tree for the theropods used in Time-calibrated phylogeny of Sinosauropterygidae shown in Fig. 3.

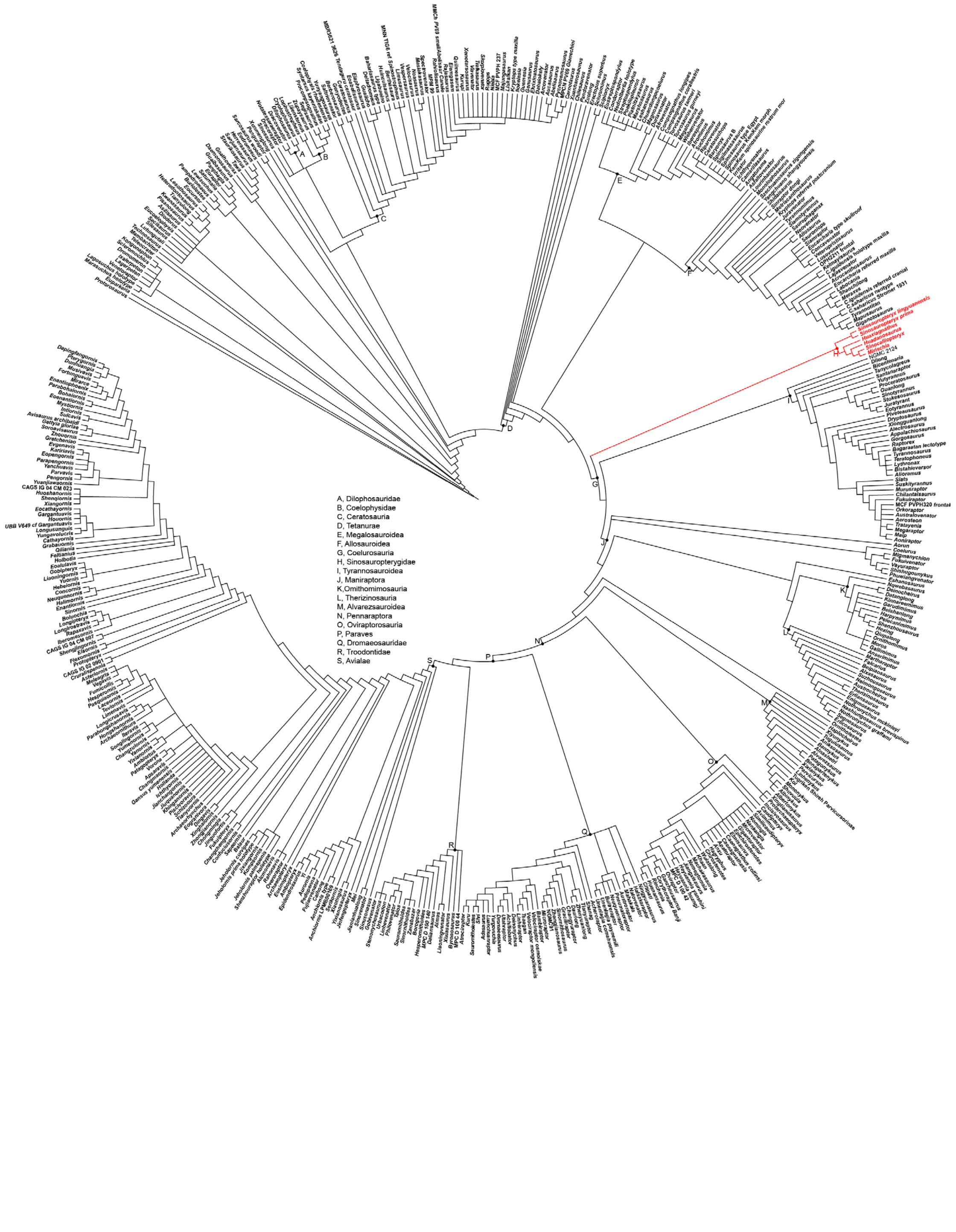

Fig. S7

Mammalian remains as the stomach content in the abdomen of *Huadanosaurus sinensis* IVPP V 14202. a, the eutherian mandibule. b, partial eutherian maxilla and lower jaw. c and d, eutriconodonts mandibules. Scale bar is 5 mm.

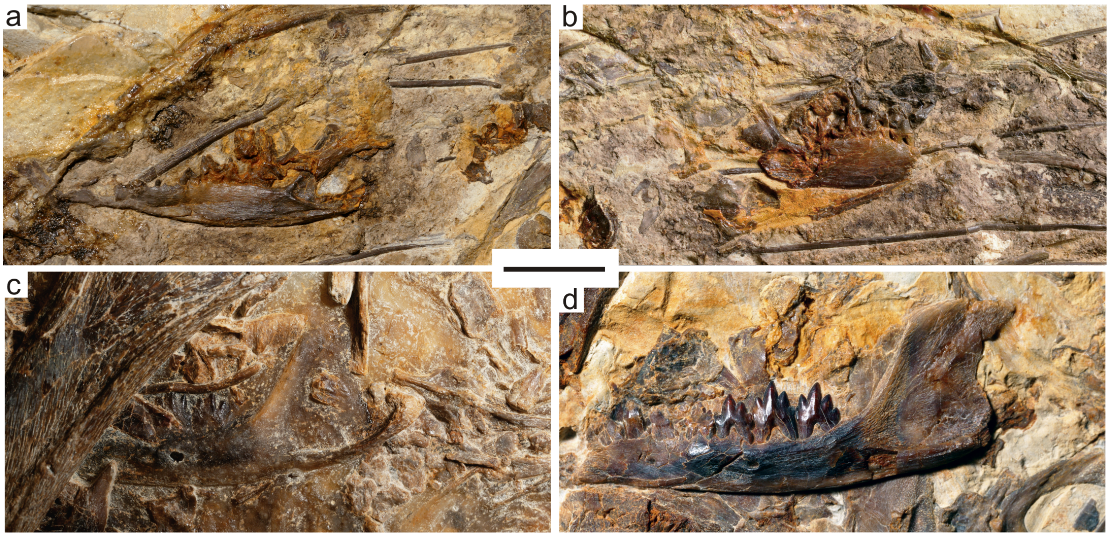

Table S1

Measurements of *Sinosauropteryx lingyuanensis* (IVPP V 12415)

Element	Length (mm)	
Skull	110.00	
Coracoid	22.54 (l)	
Humerus	39.46 (l)	38.18 (r)
Manual digit I-1		27.1 (r)
Ilium		83.83 (r)
Femur		104.67 (r)
Tibia	140.94 (l)	112.15 (r)
Metatarsus II	63.46 (l)	62.89 (r)
Metatarsus III	70.10 (l)	71.63 (r)
Metatarsus IV		62.75 (r)
Pedal digit III-1	15.94 (l)	15.64 (r)
Pedal digit III-2		12.88 (r)
Pedal digit III-3		9.19 (r)
Pedal digit IV-1		14.54 (r)
Pedal digit IV-2		8.65 (r)

Table S2

Measurements of *Huadanosaurus sinensis* (IVPP V 14202)

Element	Length (mm)	
Skull	100.59	
Scapula	56.98 (l)	55.20 (r)
Coracoid		16.06 (r)

Humerus	94.91 (l)	93.75 (r)
Ulna		36.05 (r)
Radius		33.94 (r)
Metacarpal I	9.20 (l)	8.90 (r)
Metacarpal II		20,77 (r)
Metacarpal III		16.93 (r)
Manual digit I-1		21.96(r)
Manual digit I-2		18.61 (r)
Manual digit II-1	14.61 (l)	13.19 (r)
Manual digit II-2	20.96 (l)	20.38 (r)
Manual digit II-3		16.18 (r)
Manual digit III-1		9.13 (r)
Manual digit III-2		8.58 (r)
Manual digit III-3		8.43 (r)
Ilium		70.75 (r)
Pubis		89.48 (r)
Ischium	54.01 (l)	54.26 (r)
Femur		105.69 (r)
Tibia	138.34 (l)	136.08 (r)
Fibula		131.90 (r)

Table S3

Comparsion between *S. lingyuanensis* and known *S. prima* specimens

Number	Skull	Humerus	Femur	Tibia	Metatarsus III
IVPP V 12415	110.00	38.18	104.67	112.15	65.38

NIGP 127586	62.5	20.3	53.2	61	39.9
NIGP 127587	97.2	35.5	86.4	97	64.8
DLNHM D2141	86.6	24.7	/	72.8	52.3

Table S4

Changes to the data matrix of Brusatte et al., 2014

Species	Changes		
Sinosauropteryx prima	C154 2→1<br>C159 ?→1	C630 0→1<br>C633 ?→2	C635 ?→0<br>C734 ?→0
Compsognathus longipes	C28 ?→1<br>C29 ?→1<br>C38 0→1<br>C70 0→?<br>C154 ?→1<br>C159 ?→0<br>C161 ?→1	C168 1→2<br>C255 ?→0<br>C389 ?→0<br>C395 ?→0<br>C400 ?→0<br>C631 0→1<br>C653 ?→0	C654 ?→0<br>C696 ?→1<br>C697 ?→0<br>C796 ?→1<br>C800 ?→1<br>C807 ?→0
Juravenator starki	C29 0→?<br>C38 0→1<br>C198 ?→1<br>C248 ?→0<br>C337 ?→0<br>C414 ?→1<br>C417 ?→1<br>C680 ?→1	C682 ?→1<br>C684 ?→0<br>C686 ?→1<br>C688 ?→1<br>C696 ?→1<br>C697 ?→0<br>C698 ?→1<br>C699 ?→0	C700 ?→0<br>C749 ?→1<br>C774 ?→0<br>C796 ?→1<br>C806 ?→0<br>C807 ?→0<br>C847 ?→0
Mirischia asymmetrica	C107 ?→0<br>C154 ?→0<br>C171 ?→0	C198 ?→1<br>C215 ?→1<br>C697 ?→0	C741 ?→0<br>C810 ?→0

Reference

Averianov AO, Krasnolutskii SA and Ivantsov SV. A new basal coelurosaur (Dinosauria: Theropoda) from the Middle Jurassic of Siberia. *Proc. Zool. Inst.* 2010; **314**: 42–57.

Azuma Y, Xu X and Shibata M *et al.* A bizarre theropod from the Early Cretaceous of Japan highlighting mosaic evolution among coelurosaurians.

Sci. Rep. 2016; **6**: 20478.

Bakker RT, Williams M and Currie PJ. *Nanotyrannus*, a new genus of pygmy tyrannosaur, from the latest Cretaceous of Montana. *Hunteria* 1988, **1**: 1–30.

Balanoff AM and Norell MA. Osteology of *Khaan mckennai* (Oviraptorosauria: Theropoda). *Bull. Am. Mus. Nat. Hist.* 2012; **372**: 1–77.

Brochu CA. Osteology of *Tyrannosaurus rex*: insights from a nearly complete skeleton and high-resolution computed tomographic analysis of the skull. *Soc. Vert. Paleontol. Mem.* 2003; **7**: 1–138.

Brusatte SL, Carr TD and Erickson GM *et al*. A long snouted, multihorned tyrannosaurid from the Late Cretaceous of Mongolia. *Proc. Nat. Acad. Sci. USA* 2009; **106**: 17261–17266.

Brusatte SL, Carr TD and Norell MA. The osteology of *Alioramus*, a gracile and long-snouted tyrannosaurid (Dinosauria, Theropoda) from the late Cretaceous of Mongolia. *Bull. Am. Mus. Nat. Hist.* 2012; **366**: 1–197.

Brusatte SL, Lloyd GT and Wang SC *et al*. Gradual assembly of avian body plan culminated in rapid rates of evolution across the dinosaur-bird transition. *Curr. Biol.* 2014; **24**: 2386–2392.

Burnham DA. New information on *Bambiraptor feinbergi* (Theropoda: Dromaeosauridae) from the Late Cretaceous of Montana. In: Currie PJ, Koppelhus E and Shugar M (eds.). Feathered Dragons. Bloomington and Indianapolis: Indiana University Press, 2004, 67–111.

Carr TD and Williamson TE. Diversity of late Maastrichtian Tyrannosauridae (Dinosauria: Theropoda) from western North America. *Zool. J. Linn. Soc.* 2004; **142**: 479–523.

Chen P, Dong Z and Zheng S. An exceptionally well-preserved theropod dinosaur from the Yixian Formation of China. *Nature* 1998; **391**: 147–152.

Choiniere JN, Clark JM and Forster CA *et al*. A basal coelurosaur (Dinosauria: Theropoda) from the Late Jurassic (Oxfordian) of the Shishugou Formation in Wucaiwan, People’s Republic of China. *J. Vertebr. Paleontol.* 2010; **30**: 1773–1796.

Currie PJ, Rigby JK and Sloan RE. Theropod teeth from the Judith River Formation of southern Alberta, Canada. In Carpenter K and Currie PJ (eds.). Dinosaur Systematics: Approaches and Perspective. Cambridge: Cambridge University Press, 1990, 107–125.

Currie PJ and Chen PJ. Anatomy of *Sinosauropteryx prima* from Liaoning, northeastern China. *Can. J. Earth Sci.* 2001; **38**: 1705–1727.

Currie PJ and Zhao XJ. A new carnosaur (Dinosauria, Theropoda) from the Jurassic of Xinjiang, Peoples Republic of China. *Can. J. Earth. Sci.* 1993; **30**: 2037–2081.

Chiappe LM and Göhlich UB. Anatomy of *Juravenator starki* (Theropoda: Coelurosauria) from the Late Jurassic of Germany. *Für Geol. Paläontol. - Abh.* 2010; **258**: 257–296.

Dal Sasso C and Maganuco S. *Scipionyx samniticus* (Theropoda: Compsognathidae) from the Lower Cretaceous of Italy; osteology, ontogenetic assessment, phylogeny, soft tissue anatomy taphonomy and palaeobiology. *Memorie*. 2011; **37**: 1–281.

Gold MEL, Brusatte SL and Norell MA. The cranial pneumatic sinuses of the tyrannosaurid *Alioramus* (Dinosauria, Theropoda) and the evolution of cranial pneumaticity in theropod dinosaurs. *Am. Mus. Novit.* 2013; **3790**: 1–46.

Hendrickx C, Mateus O, Araújo R and Choiniere J. The distribution of dental features in non-avian theropod dinosaurs: Taxonomic potential, degree of homoplasy, and major evolutionary trends. *Palaeontol. Electron*. 2019, **22**: 1–110.

Holtz TR. Tyrannosauroidea. In: Weishampel DB, Dodson P and Osmólska H (eds.). The Dinosauria. Berkeley: University of California Press, 2004, 1119–1136.

Hwang SH, Norell MA and Ji Q *et al*. A large compsognathid from the Early Cretaceous Yixian Formation of China. *J. Syst. Palaeontol.* 2004; **2**: 13–30.

Jasinoski SC. An integrative phylogenetic and extrapolatory approach to the reconstruction of dromaeosaur (Theropoda: Eumaniraptora) shoulder

musculature. *Zool. J. Linn. Soc.* 2006; **146**: 301–344.

Ji S, Ji Q and Lü J *et al*. A new giant compsognathid dinosaur with long filamentous integuments from Lower Cretaceous of Northeastern China. *Acata Geol. Sin.* 2007; **81**: 8–15.

Lee YN, Barsbold R and Currie PJ *et al*. Resolving the long-standing enigmas of a giant ornithomimosaur *Deinocheirus mirificus*. *Nature* 2014; **515**: 257–260.

Madsen JH. *Allosaurus fragilis*: a revised osteology. *Utah Geol. Min. Surv. Bull.* 1976; **109**: 1–163.

Madsen JH and Welles SP. Ceratosaurus (Dinosauria, Theropoda): A Revised Osteology. Utah Geological Survey: Miscellaneous Publications 00-2, 2000.

Makovicky PJ and Currie PJ. The presence of a furcula in tyrannosaurid theropods, and its phylogenetic and functional implications. *J. Vertebr. Paleontol.* 1998; **18**: 143–149.

Martill DM, Frey E and Sues HD *et al*. Skeletal remains of a small theropod dinosaur with associated soft structures form the Lower Cretaceous Santana Formation of notheastern Brazil. *Can. J. Earth Sci.* 2000; **37**: 891–900.

Naish D, Martill DM and Frey E. Ecology, Systematics and Biogeographical Relationships of Dinosaurs, Including a New Theropod, from the Santana Formation (?Albian, Early Cretaceous) of Brazil. *Hist. Biol.* 2004 **16**: 57–70.

Osborn HF. Skeletal adaptations of *Ornitholestes*, *Struthiomimus*, *Tyrannosaurus*. *Bull. Am. Mus. Nat. Hist.* 1916; **19**: 459–464.

Ostrom, J. H. Osteology of *Deinonychus antirrhopus*, an unusual theropod from the Lower Cretaceous of Montana. *Peabody Mus. Nat. Hist. Bull.* **30**, 1–165 (1969).

Ostrom, J. H. The osteology of *Compsognathus longipes*. *Zitteliana*. 1978; **4**: 73–118.

Osmólska H, Roniewicz E and Barsbold R. A new dinosaur *Gallimimus bullatus* n. gen., n. sp. (Ornithomimidae) from the Upper Cretaceous of Mongolia. *Palaeontol*. *Polonica*1972; **27**: 103–143.

Peyer K. A reconsideration of *Compsognathus* from the Upper Tithonian of Canjuers, southeastern France. *J. Vertebr. Paleontol.* 2006; **26**: 879–896.

Rauhut OWM, Milner AC and Moore-Fay S. Cranial osteology and phylogenetic position of the theropod dinosaur *Proceratosaurus bradleyi* (Woodward, 1910) from the Middle Jurassic of England. *Zool. J. Linn. Soc.* 2010; **158**: 155–195.

Reisz RR, Scott D and Sues HD. Embryos of an Early Jurassic prosauropod dinosaur and their evolutionary significance. *Science* 2005; **309**: 761–764.

Russell DA. Tyrannosaurs from the Late Cretaceous of western Canada. *NatMus. Nat. Sci. Pub. Palaeontol.* 1970; **1**: 1–34.

Sales MA, Cascon P and Schultz CL. Note on the paleobiogeography of Compsognathidae (Dinosauria: Theropoda) and its paleoecological implications. *An. Acad. Bras. Cienc.* 2014; **86**: 127–134.

Sampson SD and Witmer LM. Craniofacial anatomy of *Majungasaurus crenatissimus* (Theropoda: Abelisauridae) from the Late Cretaceous of Madagascar. *J. Vertebr. Paleontol.* 2007; **27**: 32–102.

Sanz JL, Chiappe LM and Fernádez-Jalvo Y. An early Cretaceous pellet. *Nature* 2001; **409**: 998–999.

Schmerge JD and Rothschild BM. Distribution of the dentary groove of theropod dinosaurs: Implications for theropod phylogeny and the validity of the genus *Nanotyrannus* Bakker et al., 1988. *Cret. Res*. 2016; **61**: 26–33.

Sereno PC, Tan L and Brusatte SL *et al*. Tyrannosaurid skeletal design first evolved at small body size. *Science* 2009; **326**: 418–422.

Smith JB. Heterodonty in *Tyrannosaurus re*x: implications for the taxonomic and systematic utility of theropod dentitions. *J. Vertebr. Paleontol.* 2005; **25**: 865–887.

Tsuihiji T, Watabe M and Tsogtbaatar K *et al*. Cranial osteology of a juvenile specimen of *Tarbosaurus bataar* (Theropoda, Tyrannosauridae) from the Nemegt Formation (Upper Cretaceous) of Bugin Tsav, Mongolia. *J. Vertebr. Paleontol.* 2011; **31**: 1–21.

Xu X, Norell MA and Kuang X *et al.* Basal tyrannosauroids from China and

evidence for protofeathers in tyrannosauroids. *Nature* 2004; **431**: 680–684.

Xu X, Clark JM and Forster CA *et al*. A basal tyrannosauroid dinosaur from the Late Jurassic of China. *Nature* 2006; **439**: 715–718.
